# Antiproliferative polyketides from fungus *Xylaria* cf. *Longipes* SWUF08-81 in different culture media

**DOI:** 10.1007/s13659-023-00427-7

**Published:** 2024-01-06

**Authors:** Kittiwan Sresuksai, Sasiphimol Sawadsitang, Phongphan Jantaharn, Pakin Noppawan, Audomsak Churat, Nuttika Suwannasai, Wiyada Mongkolthanaruk, Thanaset Senawong, Sarawut Tontapha, Pairot Moontragoon, Vittaya Amornkitbamrung, Sirirath McCloskey

**Affiliations:** 1 Department of Chemistry, Faculty of Science, Center of Excellence for Innovation in Chemistry (PERCH-CIC), Khon Kaen University, Khon Kaen, 40002 Thailand; 2 Department of Chemistry, Faculty of Science, Mahasarakham University, Maha Sarakham, 44150 Thailand; 3 Department of Microbiology, Faculty of Science, Srinakharinwirot University, Bangkok, 10110 Thailand; 4 Department of Microbiology, Faculty of Science, Khon Kaen University, Khon Kaen, 40002 Thailand; 5 Department of Biochemistry, Faculty of Science, Khon Kaen University, Khon Kaen, 40002 Thailand; 6 Department of Physics, Faculty of Science, Khon Kaen University, Khon Kaen, 40002 Thailand; 7 Institute of Nanomaterials Research and Innovation for Energy (IN-RIE), Khon Kaen University, Khon Kaen, 40002 Thailand

**Keywords:** *Xylaria*, Dibenzofuran, Asperentin, Pyran, Antiproliferative activity, HPLC analysis

## Abstract

Bioactive compounds from the wood-decay fungus *Xylaria* cf. *longipes* SWUF08-81, cultivated in three different culture media (GM, YM and PDB), were isolated. Their structures and stereochemistry were deduced from spectroscopic and MS data analysis, together with quantum chemical calculations of ^13^C NMR chemical shifts and electronic circular dichroism (ECD) spectra. Five undescribed polyketides including dibenzofuran (**1**), mellein (**2**), dihydroisocoumarin (**15**), and two pyrans (**16**, **17**), together with twenty-three compounds were determined. Compounds **18** and **20** were significantly toxic against cancer cell lines (HCT116, HT29, MCF-7 and HeLa) based on the MTT assay. Quantification by HPLC showed that **18** was produced three-fold higher in the broth of PDB than YM. These studies showed that the production of different compounds were primarily dependent on nutrition sources and it has given a starting point for the growth optimization conditions for the scaling up of bioactive compounds production.

**Graphical Abstract:**

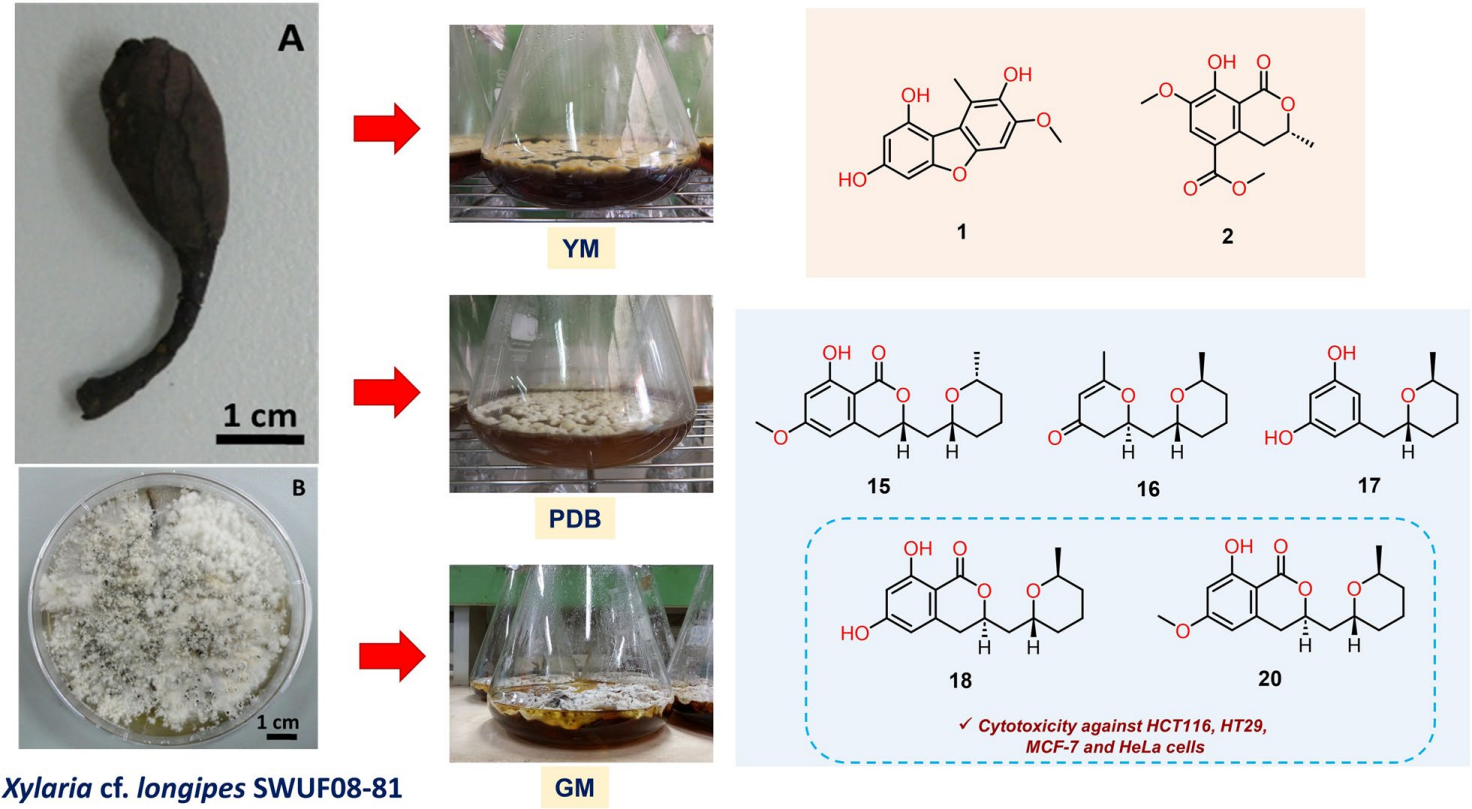

**Supplementary Information:**

The online version contains supplementary material available at 10.1007/s13659-023-00427-7.

## Introduction

Fungi are an exceptional bioactive compounds producer, many of which have been developed as drugs or drug candidates [1, 2]. The diversity of fungi metabolites is greatly influenced by the growth environment, which includes geographical location and nutrition. Examining growth conditions such as carbon and nitrogen sources, pH, temperature, stress, extreme environment and co-culture in culture media allowed for the establishment of quantity and quality profiles for compounds of interest [3–5]. The manufacture of high quantity bioactive compounds in a short period was made possible by controlling the cultivation conditions. It is for these reasons that fungi continues to be a vital source for potential drugs discovery and development [6].

The Xylariaceae family belongs to Ascomycota, known as wood-decay fungi and found throughout tropical regions [7]. *Xylaria* is one of the genera in Xylariaceae that has been studied and well documented to produce a wide range of cytotoxic compounds [8–10]. A number of previous research studies revealed that various bioactive compounds from this genus had evolved from habitat, associated climate and the complexity of the fungi species [11–14].

To continue the search for potential anticancer drugs candidates from the *Xylaria* genus, the chemical constituents from *Xylaria* cf. *longipes* SWUF08-81 in three different culture media (GM, YM and PDB) and the quantities of selected active compounds were validated. A few reports on *Xylaria longipes* showed various bioactive compounds that gave some insights into the continuing search for bioactive compounds in different environments [13–15].

Herein, we have described the results of the purification, structure elucidation, and evaluation of antiproliferative activity against cancer cells (HCT116, HT29, MCF-7 and HeLa), and normal Vero cells. Together with the quantification of cytotoxic compounds through the high-performance liquid chromatography (HPLC) method using a diode array detector (DAD).

## Results and discussion

### Purification and structure elucidation

The *X.* cf. *longipes* SWUF08-81 fungus was cultivated in three different media named GM, YM and PDB media. The compounds were extracted from broth and mycelium by organic solvents and the crude extracts were purified by chromatographic separation. Fourteen compounds (**1**–**14**) were isolated from GM medium, and fourteen compounds (**15–28**) were isolated from YM medium. Eight compounds were isolated from PDB medium, all of which were also identified from the YM medium, including compounds **17, 18–22**, **24** and **26**.

**Fig. 1:**
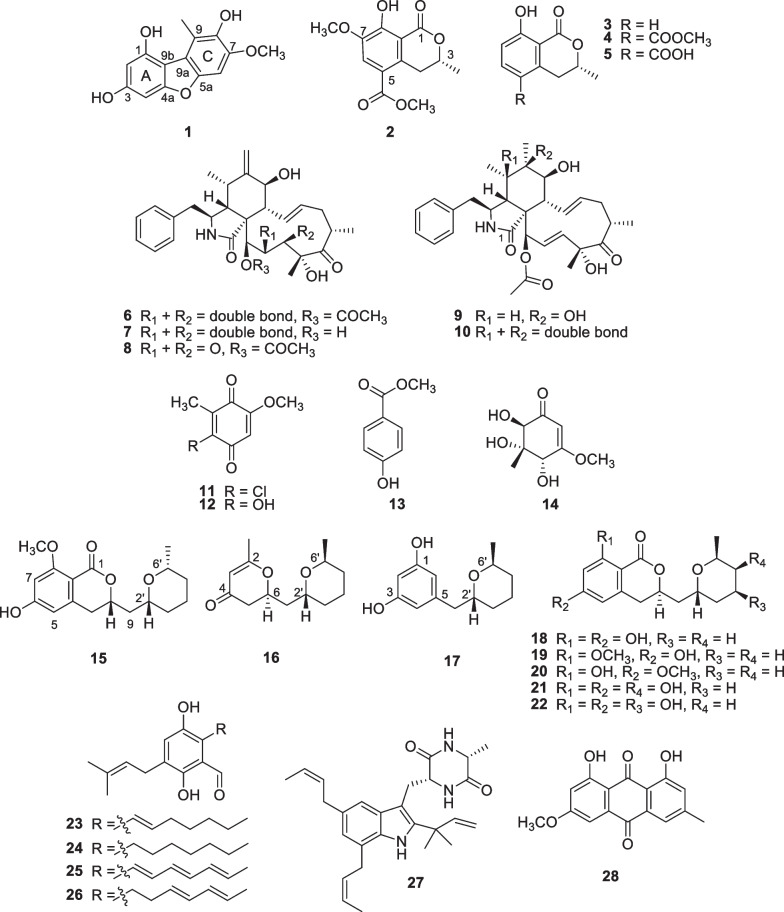
The isolated compounds from *Xylaria* cf. *longipes* SWUF08-81 in three different culture media

The analysis of spectroscopic and MS data, in combination with the ^13^C NMR chemical shift and the quantum chemical ECD calculations [16, 17], revealed two undescribed polyketides 1,3,8-trihydroxy-7-methoxy-9-methyldibenzofuran (**1**) and (3*R*)-7-methoxy-5-methoxycarbonylmellein (**2**) form GM medium, together with twelve known compounds including (−)-mellein (**3**) [18], (−)-5-methoxycarbonyl- mellein (**4**) [19], (−)-5-carboxymellein (**5**) [19], cytochalasin D (**6**) [19], zygosporin D (**7**) [20], 19,20-epoxycytochalasin D (**8**) [21], cytochalasin O_hyp_ (**9**) [22], cytochalasin C (**10**) [22], 2-chloro-5-methoxy-3-methylcyclohexa-2,5-diene-1,4-dione (**11**) [23], 2-hydroxy-5-methoxy-3-methyl cyclo-hexa-2,5-diene-1,4-dione (**12**) [24], 4-hydroxy-methylbenzoate (**13**) [25] and (4*R*,5*S*,6*R*)-4,5,6-trihydroxy-3-methoxy-5-methyl-cyclohex-2-n-1-one (**14**) [23]. There were three undescribed compounds, including dihydroisocoumarin, (3*R*,2′*R*,6′*R*)-asperentin-8-*O*-methylether (**15**) and two pyran derivatives, (6*S*,2′*R*,6′*S*)-6-methyl-2-((6-methyltetrahydro-2*H*-pyran-2-yl)methyl)-2,3-dihydro-4*H*-pyran-4-one (**16**) and (2′*R*,6′*S*)-5-((-6-methyltetrahydro-2*H*-pyran-2-yl)methyl)benzene-1,3-diol (**17**) from YM, together with eleven known compounds including five dihydroisocoumarins, asperentin (**18**) [26, 27], asperentin-8-*O*-methylether (**19**) [27, 28], asperentin-6-*O*-methylether (**20**) [26], 5′-hydroxyasperentin (**21**) [26] and 4′-hydroxyasperentin (**22**) [26], four auroglaucins, tetrahydroauroglaucin (**23**) [29], flavoglaucin (**24**) [29], auroglaucin (**25**) [29] and isodihydroauroglaucin (**26**) [29], alkaloid, echinulin (**27**) [30], and anthraquinone, physcion (**28**) derivatives [31] (Fig. 1).

**Fig. 2:**
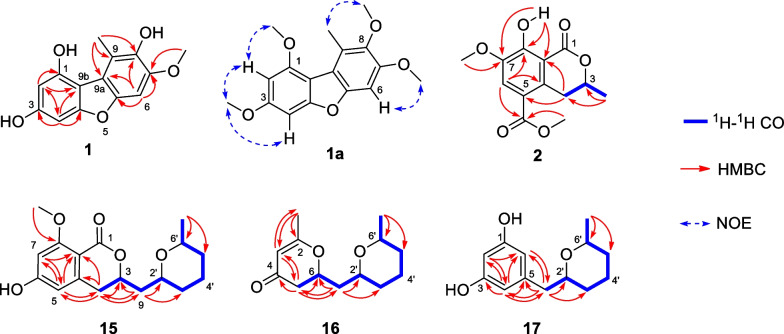
Key ^1^H-^1^H COSY, HMBC and NOE correlations of compounds **1**, **1a, 2** and** 15–17**

Compound **1** was a brown amorphous solid with a melting point of 224–225 °C. Its molecular formula of C_14_H_12_O_5_ was deduced from the [M + Na]^+^ peak at *m/z* 283.0597 in the HRESIMS (calcd. for C_14_H_12_O_5_Na^+^, 283.0577). The key IR spectrum showed absorption bands of O–H stretching at 3354 cm^−1^ and aromatic C = C stretching at 1615, 1509 and 1462 cm^−1^. The ^1^H NMR data (Table 1) displayed proton signals of an aromatic at *δ*_H_ 6.92 (1H, s, H-6), 6.40 (1H, d, *J* = 1.7 Hz, H-4) and 6.22 (1H, d, *J* = 1.7 Hz, H-2), a methoxy at *δ*_H_ 3.91 (3H, s, H-7-OCH_3_), and a methyl at *δ*_H_ 2.77 (3H, s, H-9-CH_3_). The ^13^C NMR data (Table 1) showed signals of aromatic carbons at *δ*_C_ 159.1 (C-3), 156.6 (C-4a), 152.0 (C-1), 149.1 (C-5a), 145.8 (C-7), 140.1 (C-8), 117.6 (C-9), 116.5 (C-9a), 106.1 (C-9b), 97.0 (C-2), 91.7 (C-6) and 89.4 (C-4). There was a methoxy carbon signal at *δ*_C_ 55.3 (C-7-OCH_3_) and a methyl carbon signal at 13.2 (C-9-CH_3_). There was no COSY correlation observed in **1**, therefore the structure was deduced from key HMBC correlations between H-2 to C-1, C-4 and C-9b, and H-4 to C-2, C-3, C-4a and C-9b, confirming the tetrasubstituted aromatic ring A. The aromatic ring C was indicated by the correlations between H-6 to C-5a, C-7, C-8 and C-9a, and 9-CH_3_ to C-8, C-9 and C-9a. When compared to those in literature, the NMR data of **1** suggested that **1** shared a dibenzofuran scaffold similar to the known 2,7-dihydroxy-1,6-dimethoxy-9-methyldibenzofuran [32, 33]. The complete structure was confirmed by the NOE experiment of the methylated product, **1a** (Fig. 2). The correlation between H-1-OCH_3_ and H-2, H-3-OCH_3_ and H-4, H-7-OCH_3_ and H-6, and H-8-OCH_3_ and H-9-CH_3_ confirmed the methoxy positions on both aromatic rings. Therefore, **1** was deduced as a new dibenzofuran, named 1,3,8-trihydroxy-7-methoxy-9-methyldibenzofuran.

**Table 1:** ^1^H and ^13^C NMR data of compounds **1**, **1a** and **2** (*δ* in ppm, *J* in Hz)

Position	**1**^*a*^		**1a**^*b*^		**2**^*c*^	
	*δ*_H_	*δ*_C_	*δ*_H_	*δ*_C_	*δ*_H_	*δ*_C_
1		152.0		154.3		170.4
2	6.22 (d, 1.7)	97.0	6.38 (d, 2.0)	93.8		
3		159.1		159.5	4.65 (dqd, 12.5, 6.3, 3.2)	76.1
4	6.40 (d, 1.7)	89.4	6.67 (d, 2.0)	88.4	3.82 (dd, 17.6, 3.2) 3.00 (dd, 17.6, 11.7)	32.2
4a		156.6		158.4		134.3
5						117.8
5a		149.1		152.0		
6	6.92 (s)	91.7	6.91 (s)	92.9	7.67 (s)	118.5
7		145.8		151.4		146.9
8		140.1		143.8		156.4
8a						108.9
9		117.6		126.1		
9a		116.5		116.0		
9b		106.1		112.0		
1-OCH_3_			3.95 (s)	55.2		
3-OCH_3_			3.88 (s)	55.6		
3-CH_3_					1.55 (d, 6.3)	20.7
5-CO						166.2
5-COOCH_3_					3.89 (s)	52.1
7-OCH_3_	3.91 (s)	55.3	3.92 (s)	55.8	3.94 (s)	56.3
8-OCH_3_			3.80 (s)	60.5		
9-CH_3_	2.77 (s)	13.2	2.80 (s)	14.4		
8-OH					12.10 (s)	

^a^Recorded at 500 MHz in CD_3_OD

^b^Recorded at 500 MHz in CDCl_3_

^c^Recorded at 400 MHz in CDCl_3_

**Fig. 3:**
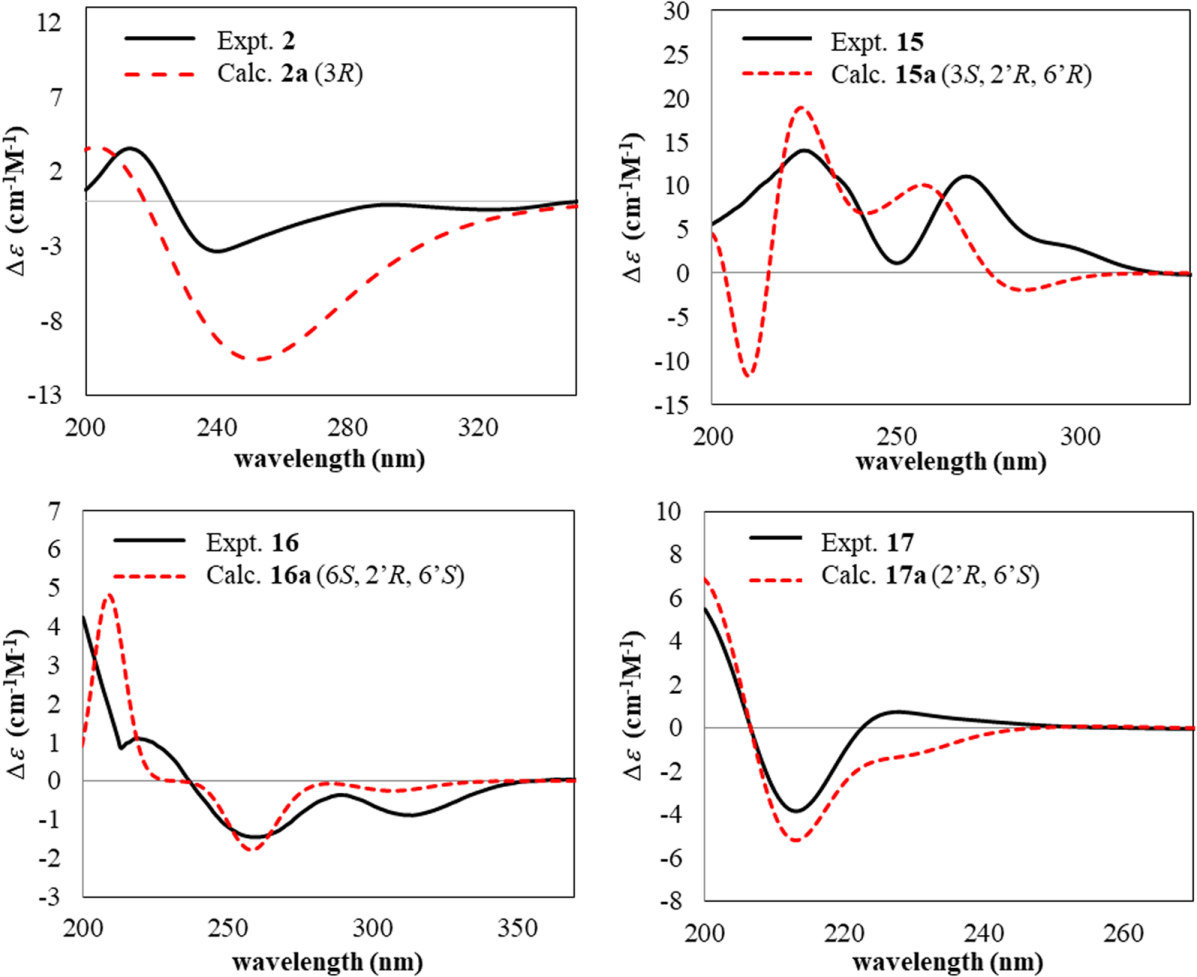
Comparison of experimental and calculated ECD spectra of **2** and** 15–17**

Compound **2** was a white amorphous solid with a melting point of 135–136 °C. The [M + Na]^+^ peak at m/z 289.0687 in the HRESIMS indicated a molecular formula of C_13_H_14_O_6_ (calcd. for C_14_H_12_O_5_Na^+^, 289.0683). The IR spectrum showed absorption bands of C = O stretching at 1713 and 1669 cm^−1^, and aromatic C = C stretching at 1589, 1477 and 1432 cm^−1^. The ^1^H NMR data (Table 1) displayed proton signals of an aromatic at *δ*_H_ 7.67 (1H, s, H-6), an oxygenated methine at *δ*_H_ 4.63 (1H, 4.65 (dqd, *J* = 12.5, 6.3, 3.2 Hz, H-3), two methylene at *δ*_H_ 3.82 (2H, dd, *J* = 17.6, 3.2 Hz, H-4) and 3.00 (dd, *J* = 17.6, 11.7 Hz, H-4), two methoxy at *δ*_H_ 3.94 (3H, s, H-7-OCH3) and 3.89 (3H, s, 5-COOCH_3_) and a methyl at *δ*_H_ 1.55 (3H, d, *J* = 6.3 Hz, H-3-CH_3_). Based on ^1^H NMR data, the signals of aromatic carbons were in the range of *δ*_C_ 108.9–156.4, oxygenated methine carbon at *δ*_C_ 76.1 (C-3) and methoxy carbons at *δ*_C_ 56.3 (C-7-OCH_3_) and 52.1 (C-5-COOCH_3_). In addition, there were carbonyl carbons signals at *δ*_C_ 170.4 (C-1) and 166.2 (C-5-CO). The COSY correlation of H-3 to H-4 and H-3-CH_3_, and the HMBC correlations of H-3-CH_3_ to C-3 and C-4, and H-4 to C-3, C-4a and C-8a, suggested a lactone ring. The tetrasubstituted aromatic ring was confirmed by the correlations of H-6 to C-4a, C-5, C-7 and C-8, H-7-OCH3 to C-7 and H-8-OH to C-7, C-8 and C-8a. The above data suggested a mellein analogue of **2**. The methoxycarbonyl group at C-5 was confirmed by correlations of H-4 to C-4a and C-5, H-6 to C-5 and C-5-CO and H-5-COOCH_3_ to C-5-CO, which, together with a hydrogen-bonded hydroxyl proton signal at *δ*_H_ 12.10 (1H, s, H-8-OH), confirmed the presence of a hydroxy group at C-8. The absolute configuration at C-3 was deduced by a comparison of the specific rotation [α]_D_^25.6^ -123.6 (*c* 1.0, CHCl_3_) of **2** with the known mellein core-structure, which were [α]_D_^24.6^ -44.8 (*c* 1.0, CHCl_3_) for *R* and [α]_D_ + 92° (*c* 1.14, MeOH) for *S* as observed in a mellein (**3**) [18]. This was proved through a comparison of experimental and calculated ECD spectra (Fig. 3). The experimental ECD spectrum showed cotton effects at 204 nm (Δ*ε* =  + 4.52) and 251 nm (Δ*ε* =—11.70), which closely matched the calculated 3*R* isomer. Therefore, it was concluded that **2** was a new mellein derivative, named (3*R*)-7-methoxy-5-methoxycarbonyl mellein.

Compound **15** was a yellow viscous liquid. The molecular formula of C_17_H_22_O_5_ was deduced from the [M + H]^+^ peak at *m/z* 307.1546 in the HRESIMS (calcd. for C_17_H_23_O_5_^+^, 307.1540). The key IR absorption bands of O–H stretching and C = O stretching of ester were at 3242 cm^−1^ and 1689 cm^−1^, respectively. The ^1^H NMR data (Table 2) showed that there were proton signals of two aromatics at *δ*_H_ 6.40 (1H, s, H-7) and 6.26 (1H, s, H-5), three oxymethines at *δ*_H_ 4.65 (1H,br t, *J* = 10.5 Hz, H-3), 3.67 (1H, t, *J* = 10.7 Hz, H-2′) and 3.43 (1H, m, H-6′), a methoxy at *δ*_H_ 3.80 (3H, s, H-8-OCH_3_), and a methyl at *δ*_H_ 1.29 (3H, d, *J* = 6.0 Hz, H-6′-CH_3_).

**Table 2:** ^1^H and ^13^C NMR data of compounds **15–17** in CDCl_3_ (500 MHz, *δ* in ppm, *J* in Hz)

Position	**15**		**16**		**17**	
	*δ*_H_	*δ*_C_	*δ*_H_	*δ*_C_	*δ*_H_	*δ*_C_
1		164.4				156.9
2				174.2	6.16 (1H, t, 2.3)	100.9
3	4.65 (1H, br t, 10.5)	74.3	5.32 (1H, s)	104.9		156.9
4	2.76 (1H, dd, 16.0, 11.4) 2.67 (1H, d, 16.0)	35.1		193.0	6.23 (1H, d, 2.3)	108.7
4a		144.2				
5	6.26 (1H, s)	106.8	2.42 (1H, d, 7.7) 2.40 (1H, d, 1.6)	41.5		141.9
6		162.9	4.57 (1H, m)	76.2	6.23 (1H, d, 2.3)	108.7
7	6.40 (1H, s)	98.7	1.98 (1H, m) 1.77 (1H, m)	38.5	2.87 (1H, dd, 13.6, 7.1) 2.64 (1H, dd, 13.6, 7.1)	39.2
8		163.5				
8a		105.4				
9	1.84 (1H, m) 1.71 (1H, m)	41.6				
2′	3.67 (1H, t, 10.7)	73.3	4.07 (1H, m)	66.5	4.06 (1H, m)	72.7
3′	1.55 (1H, m) 1.19 (1H, m)	31.7	1.69 (1H, m) 1.33 (1H, m)	30.4	1.69 (1H, m) 1.43 (1H, m)	28.7
4′	1.81 (1H, m) 1.53 (1H, m)	23.5	1.64 (1H, m) 1.69 (1H, m)	18.4	1.69 (1H, m)	18.2
5′	1.55 (1H, m) 1.19 (1H m)	33.2	1.69 (1H, m) 1.33 (1H, m)	31.4	1.69 (1H, m) 1.33 (1H, m)	31.8
6′	3.43 (1H, m)	74.0	3.87 (1H, pd, 6.4, 3.2)	67.0	4.03 (1H, m)	67.4
2-CH_3_			1.99 (3H, s)	21.0		
6′-CH_3_	1.13 (3H, d, 6.0)	22.0	1.18 (3H, d, 6.4)	19.5	1.17 (3H, d, 6.5)	20.0
8-OCH_3_	3.80 (3H, s)	55.8				

The spectrum also showed signals of methylene protons at *δ*_H_ 2.76 (1H, dd, *J* = 16.0, 11.4 Hz, H-4) and 2.67 (1H, d, *J* = 16.0 Hz, H-4) together with other methylene protons at the range of *δ*_H_ 1.1 to 1.8 (m, H-9 and H-3′ to H-5′). The ^13^C NMR data (Table 2) showed carbon signals of carbonyl ester at *δ*_C_ 164.4 (C-1), six aromatics at *δ*_C_ 163.5 (C-8), 162.9 (C-6), 144.2 (C-4a), 106.8 (C-5), 105.4 (C-8a) and 98.7 (C-7), and three oxygenated at *δ*_C_ 74.3 (C-3), 74.0 (C-6′) and 73.3 (C-2′).

The COSY correlations (Fig. 2) between H-3 to H-4 and H-9 together with the HMBC correlation of H-4 to C-3, C-4a, C-5, and C-8a, H-5 to C-4, C-7 and C-8a, H-7 to C-5, C-6 and C-8a suggested a 3,4-dihydroisocoumarin structure and a methoxy group at C-8 (*δ*_C_ 163.5). This was confirmed by the correlation of OCH_3_-8 to C-8. The correlations between H-2′ to H-3′, H-4′ to H-5′ and H-6' to H-6′-CH_3_ along with the correlation of H-6′-CH_3_ to C-5′ and C-6′suggested a tetrahydropyran ring. The connections between 3,4-dihydroisocoumarin and tetrahydropyran were confirmed by the correlations between H-9 to H-3 and H-2′. Based on the analysis of the NMR data and the comparison of those to literature, compound **15** resembled the known isocoumarins, (3*R*,2′*R*,6′*S*)-asperentin-8-*O*-methylether (**19**) [34]. However, the ^13^C NMR data of stereogenic centers at C-3 (*δ*_C_ 74.3), C-2′ (*δ*_C_ 73.3) and C-6′ (*δ*_C_ 74.0) shifted to low field, suggested a different stereochemistry. The correlations between H-2′ and H-6′ from NOE experiments, suggested that both were on the same face of the tetrahydropyran ring. The ^13^C NMR chemical shift calculation of two possible diastereomers were calculated (GIAO calculation using CPAM model in chloroform at the B3LYP/6-31G(d,p) level of theory) and the calculated ECD spectra for 3*S*,2′*R*,6′*R* **(15a)**, 3*R*,2′*S*,6′*S* **(15b)**, 3*S*,2′*S*,6′*S* **(15c)** and 3*R*,2′*R*,6′*R* **(15d)** were generated (TD-DFT calculation using CPAM model in DCM at the CAM-B3LYP/6–311 +  + G(d,p) level of theory) (See SI). The ECD spectrum generated for a 3*S*,2′*R*,6′*R* (**15a**) has similarities to the experimental spectrum of **15** (Fig. 3), together with 91.24% of DP4 + (C data) probability analysis, which indicates the absolute configuration of **15** to be 3*S*,2′*R*,6′*R*. It can be concluded that **15** was a new asperentin derivative, named (3*S*,2′*R*,6′*R*)-asperentin-8-*O*-methylether.

Compound **16** was a brown viscous liquid and the [M + Na]^+^ peak at *m/z* 247.1304 in the HRESIMS suggested the molecular formula C_13_H_20_O_3_ (calcd. for C_13_H_20_O_3_Na^+^, 247.1305). The key IR absorption bands were the C = O stretching of an unsaturated carbonyl ketone at 1666 cm^−1^ and a C = C stretching at 1610 cm^−1^. The ^1^H NMR data (Table 2) showed proton signals of an olefinic at *δ*_H_ 5.32 (1H, s, H-3), three oxymethines at *δ*_H_ 4.57 (1H, m, H-6), 4.07 (1H, m, H-2′) and 3.87 (1H, pd, *J* = 6.4, 3.2 Hz, H-6′), five methylene at the range of *δ*_H_ 1.33–2.42 (H-5, H-7, H-3′, H-4′ and H-5′), and two methyls at *δ*_H_ 1.99 (3H, s, H-2-CH_3_) and 1.18 (3H, d, *J* = 6.4 Hz, CH_3_-6). The ^13^C NMR of **16** showed carbon signals of an *α,β*-unsaturated ketone at *δ*_C_ 193.0, (C-4), two olefinics at *δ*_C_ 174.2 (C-2) and 104.9 (C-3), and three oxymethines at *δ*_C_ 76.2 (C-6), 67.0 (C-6′) and 66.5 (C-2′). The COSY correlations (Fig. 2) between H-5 to H-6 and the HMBC correlation between H-5 to C-4 and C-6, H-3 to C-2, C-5 and C-2-CH_3_, and H-2-CH_3_ to C-2 and C-3 indicated a 2,3-dihydropyran-4-one ring. The correlations between H-2′ to H-7, and H-6′ to H-5′ and H-6′-CH_3_ together with correlations of H-5′ to C-3′ and C-6′, H-6′-CH_3_ to C-5′ and C-6′ suggested a tetrahydropyran ring. The connection of both rings was confirmed by correlations between H-6, H-7 and H-2′ and between H-5 to C-7 and H-7 to C-2′. The ^13^C NMR chemical shifts of possible diastereomers were estimated (GIAO calculation using CPAM model in chloroform at the B3LYP/6-31G(d,p) level of theory), and the ECD spectra for 6*S*,2′*R*,6′*S* (**16a)**, 6*R*,2′*S*,6′*R* (**16b)**, 6*S*,2′*S*,6′*R* (**16c)** and 6*R*,2′*R*,6′*S* (**16d**) were generated (TD-DFT calculation using CPAM model in DCM at the CAM-B3LYP/6–311 +  + G(d,p) level of theory) (See SI). In combination with a DP4 + (C data) probability analysis result of 100% and the ECD spectrum of the 6*S*,2′*R*,6′*S* (**16a)** isomer, which was similar to the experimental spectrum **16,** (Fig. 3), confirmed the absolute configuration of **16** as 6*S*,2′*R*,6′*S*. Thus compound **16** was identified as the new (6*S*,2′*R*,6′*S*)-6-methyl-2-((6-methyltetrahydro-2*H*-pyran-2-yl)methyl)-2,3-dihydro-4*H*-pyran-4-one.

Compound **17** was a brown viscous liquid. The molecular formula of C_13_H_18_O_3_ was deduced from the [M + H]^+^ peak at *m/z* 223.1320 in the HRESIMS (calcd. for C_13_H_19_O_3_^+^, 223.1329). The IR spectrum showed absorption bands of O–H stretching at 3269 cm^−1^ and C = C stretching at 1601 cm^−1^. From the ^1^H NMR spectrum, there were signals suggesting the existence of a tetrahydropyran comparable to **16** (Table 2). The additional signals were three aromatic protons at *δ*_H_ 6.23 (2H, d, *J* = 2.3 Hz, H-4 and H-6) and 6.16 (1H, t, *J* = 2.3 Hz, H-2), which corresponded to six aromatic carbons signals at *δ*_C_ 156.9 (C-1 and C-3), 108.7 (C-4 and C-6) and 100.9 (C-2). These suggested the presence of a 1,3,5-trisubstituted aromatic ring, which was confirmed by the HMBC correlations (Fig. 2) between H-6 to C-1 and C-7, H-2 to C-1, C-3, C-4 and C-6, and H-4 to C-3. The correlations between H-7 to C-4, C-5, C-6, C-2′ and C-3′ confirmed a connection between tetrahydropyran and the aromatic ring. The ^13^C NMR chemical shifts of two diastereomers were calculated (GIAO calculation using CPAM model in chloroform at the B3LYP/6-31G(d,p) level of theory) and the results suggested a 2′*R,*6′*S* or 2′*S,*6′*R* configuration. The calculated ECD spectra of 2′*R,*6′*S* **(17a)**, 2′*S,*6′*R* (**17b)**, 2′*R,*6′*R* (**17c)** and 2′*S,*6′*S* (**17d)** were generated (TD-DFT calculation using CPAM model in DCM at the CAM-B3LYP/6–311 +  + G(d,p) level of theory) (See SI). The ECD spectrum generated for 2′*R*,6′*S* (**17a)** was the best fit to the experimental ECD (Fig. 3), which corresponded to a DP4 + (C data) probability of 100%, confirming the absolute configuration of **17** as 2′*R*,6′*S*. These led to the conclusion that compound **17** was a new (2′*R*,6′*S*)-5-((-6-methyltetrahydro-2*H*-pyran-2-yl)methyl)benzene-1,3-diol.

### Antiproliferative activity

The antiproliferative activity against HCT116, HT29, MCF-7, HeLa and Vero cells of compounds **1**–**5**, **15**, and **17–22** were evaluated (Table 3), based on the amount isolated, whether they were known or new compounds and if they had similar core structures. Compound **18** showed a strong broad spectrum against all cancer cells and normal cells with the IC_50_ in the range of 2.09–10.09 µg/mL. Compounds **1**, **17** and **20** showed moderate anti-proliferation against all cancer cells but were not toxic toward normal cells, while **5** and **15** were slightly toxic against HCT116, HT29, and HeLa, but not toxic toward MCF-7 and normal cells. Based on the core structure of the tested compounds, for example, asperentin derivatives (**15** and **18–22**), the phenolic and the hydroxy group on tetrahydropyran may play a role in their antiproliferative activity.

**Table 3:** MTT assay results of the antiproliferative activity of compounds **1**–**5**, **15** and **17**–**22** against four cancer lines and Vero cells

Samples	Antiproliferative activity, IC_50_ ± SD (*μ*g/mL)				
	Vero cells	HCT116	HT29	MCF-7	HeLa
**1**	25.00 ± 0.63	14.36 ± 0.87	8.99 ± 1.96	18.40 ± 0.99	16.68 ± 0.55
**2**	> 100	92.93 ± 2.70	96.42 ± 3.37	> 100	> 100
**3**	> 100	> 100	> 100	> 100	> 100
**4**	> 100	> 100	> 100	> 100	> 100
**5**	> 100	54.88 ± 3.35	72.64 ± 2.00	> 100	57.76 ± 1.26
**15**	> 100	45.09 ± 3.65	67.60 ± 6.99	> 100	92.93 ± 2.70
**17**	> 100	17.50 ± 1.29	19.49 ± 0.77	50.24 ± 3.85	13.53 ± 1.15
**18**	8.77 ± 0.91	2.42 ± 0.20	4.51 ± 0.13	10.09 ± 0.52	2.09 ± 0.16
**19**	> 100	66.49 ± 7.68	> 100	> 100	> 100
**20**	> 100	8.69 ± 0.69	33.26 ± 4.42	11.71 ± 0.82	4.98 ± 0.40
**21**	> 100	> 100	> 100	> 100	> 100
**22**	> 100	> 100	> 100	> 100	> 100
Cisplatin^*a*^	1.97 ± 0.24	1.48 ± 0.23	1.59 ± 0.07	3.13 ± 0.26	1.93 ± 0.04

IC_50_ values represent concentrations of the indicated compounds that inhibit 50% of cell proliferation in triplicate and reported as mean ± SD (μg/mL)

^a^Positive control

### HPLC analysis of crude extracts from *X.* cf. *longipes* SWUF08-81

The compounds isolated from *X.* cf*. longipes* SWUF08-81, when cultivated in PBD and YM, were the same. This corresponded to the preliminary examination of crude EtOAc extracts from broth and mycelium by HPLC compound profiling (See SI). The intensity of the cytotoxic compounds **18** and **20** in HPLC profiles were notably different in both media. The production of **18** and **20** were validated by HPLC–DAD, which revealed the considerable difference in their production. The results showed that **18** was produced and seceded into the liquid media, as it was quantified nearly ten times more in broth than mycelium in both media. In addition, it was produced approximately three times more in PDB than the YM broth. Compound **20** was produced only one times more in the YM than the PDB and roughly the same amount was found in both broth and mycelium.

In conclusion, the study of chemical constituents from *X*. cf. *longipes* SWUF08-81, cultivated in three media (GM, YM and PDB) revealed fourteen compounds (**1**–**14**) from GM, fourteen compounds (**15**–**28**) from YM and eight compounds (**18**, **19–23**, **25**, **27**) from PDB. They were structurally categorized into various groups of various bioactive natural products including dibenzofuran (**1**), melleins (**2**–**5**), cytochalasins (**6**–**10**), monocyclic polyketides (**11**–**14**), dihydroisocoumarins (**15** and **18**–**22**), tetrahydro-2*H*-pyran derivatives (**16** and **17**), auroglaucins (**23**–**26**), alkaloid (**27**) and anthraquinone (**28**), in which five of them (**1–2** and **15–17**) were undescribed compounds. It is worth noting that the cytochalasins have previously been reported from *X*. *longipes*, however there were no diterpenoids found in this work [13–15, 35]. The results showed that the production of the compounds in GM medium were entirely different from the YM and PDB media, which may be due to the different sources and carbon and nitrogen ratio [36, 37]. In the YM and PDB, the same group of compounds were identified, however when investigated, the production of **18** and **20** showed that the PDB medium is the best medium to start with to increase the quantity of **18**.

This study demonstrated that fungi remain one of the most important sources of a wide variety of bioactive compounds that can be regulated to larger production scales in the laboratory or industry. In addition, the fungus *X*. cf. *longipes* SWUF08-81 proved to be a potential source of anticancer agents that could be comprehensively explored in terms of optimizing growth conditions to increase the production of active compounds for molecular level studies.

## Materials and methods

### General experimental procedures

Melting points were measured on a Gallenkamp SANYO MPU250BM3.5 melting point apparatus (Gallenkamp, London, UK). Optical rotation was recorded on a JASCO DIP-1000 digital polarimeter (JASCO Corporation, Tokyo, Japan). The UV and ECD spectra were measured on a JASCO J-810 CD spectrometer (JASCO Corporation, Tokyo, Japan. IR spectra were obtained using a BRUKER TENSOR 27 FT-IR spectrophotometer (Bruker Optics, Kowloon Bay, Hong Kong). ^1^H and ^13^C NMR spectra were recorded on a Varian Mercury plus 400 MHz (Oxford Instruments, Abingdon, UK) and BRUKER 400 and 500 MHz NMR spectrometers (Bruker BioSpin AG, Fällanden, Switzerland). Electrospray Ionization (ESI) mass spectra were measured on a micrOTOF Bruker mass spectrometer (Bruker Daltonics, Kanagawa, Japan). High Performance Liquid Chromatography (HPLC) for separation was performed on a Shodex RI-101 chromatographer equipped with a Hitachi L 6000 pump and Shimadzu SPD-20A UV–Vis detector (Shimadzu, Kyoto, Japan) using a Cosmosil 5SL-II 4.6 ID × 250 mm normal phase column (Nacalai Tesque Inc., Kyoto, Japan. For chemical profiling, HPLC was performed on the Agilent 1220 LC system VL (Agilent, Germany), a binary pump, a manual injector with a sample loop of 10 µL and an Agilent 1260 infinity DAD detector. Column chromatography (CC) and flash column chromatography (FCC) were performed on silica gel 60 (0.063–0.200 mm and less than 0.063 mm mesh, MERCK, Germany). Sephadex LH-20 for chromatography (SIGMA-ALDRICH, Sweden) was used for size exclusion chromatography. Thin layer chromatography (TLC) analysis was carried out on silica gel 60 F_254_ aluminum sheets. The TLC sheet was visualized under UV light (254 and 366 nm) and it was further stained by anisaldehyde reagent and then heated until spots of compounds appeared (Additional file 1).

### Fungal material

The fungal stroma was collected from Phu Khieo Wildlife Sanctuary in Chaiyaphum Province and deposited at Srinakharinwirot University Fungal Herbarium (SWUF) under the access number of SWUF08-81. The fungal pure culture obtained from ascospores was grown on potato dextrose agar at 30ºC. The morphological characteristics including stromal surface, shape, size and color were carefully observed under stereomicroscope. Its morphology was similar to *Xylaria longipes* Nitschke described by Rogers except that the ascospore size was slightly smaller (11–14 µM length × 4.5–5 µM width) and the germ slit was straight and ran the full length of the spore [38]. The nucleotide sequences of internal transcribed spacers (ITS) were analyzed and resulted in 100% similarity to *X. longipes* (AF163038). Therefore, we noted the fungus name as *X*. cf. *longipes,* as morphological characteristics of the fungus were slightly different [39]. The ITS sequence was submitted to GenBank database as accession number OP082329.

### Fermentation, extraction, and isolation

The ascospores were isolated from mature fungal stroma and cultivated on potato dextrose agar (PDA). The pure cultures were individually inoculated into 59 L of Glucose-Malt extract (called GM) medium (containing glucose (60 g/L) and malt extract (20 g/L)), 14 L of Yeast extract-Malt extract (called YM) medium (containing yeast extract (3 g/L), malt extract (3 g/L), peptone (5 g/L) and dextrose (10 g/L)) and 14 L of Potato Dextrose Broth (PDB) medium (containing potatoes (200 g/L) and dextrose (20 g/L)). The cultures were kept at room temperature for 45 days and then each of the fermented broths was filtered and partitioned with EtOAc by liquid–liquid extraction. Removal of EtOAc gave crude EtOAc broth extracts from GM medium (128.15 g, 14.48%), YM medium (9.86 g, 18.58%) and PDB medium (5.15 g, 9.90%). The mycelium from the three media were separately dried in an oven at 50 °C for 3 days. The dried mycelium (885.09 g (GM), 53.06 g (YM) and 52.03 g (PDB)) were ground and successively extracted with EtOAc (500 mL × 3) and the removal of solvents gave mycelium crude extracts from GM (45.44 g, 5.13%), YM (7.09 g, 19.37%) and PDB (3.92 g, 7.53%). The purification procedures of the undescribed compounds **1**, **2**, **15**–**20** are described herein (for known compounds see SI).

The crude EtOAc extract of GM broth (128.15 g) was purified by silica gel CC eluted with a gradient system of EtOAc-hexane (0:1–1:0) and MeOH-EtOAc (0:1–1:0) to give 10 fractions, GB_1_-GB_10_. Fraction GB_3_ was purified by FCC eluted with a gradient system of EtOAc-hexane (0.5:9.5–1:0) to get 14 fractions, GB_3.1_-GB_3.14_. Fraction GB_3.7_ was separated by FCC eluted with 2:3 EtOAc-hexane to give **2** (109.3 mg, 0.012%). Fractions GB_3.12.1_-GB_3.12.4_ were obtained by the fractionation of GB_3.12_ by FCC eluted with 3:2 EtOAc-CH_2_Cl_2_. Fraction GB_3.12.1_ was purified by PLC developed with 1:7:2 MeOH-CH_2_Cl_2_-hexane to yield **1** (13.9 mg, 0.002%).

The crude EtOAc extract from YM broth (9.86 g) was purified by silica gel CC and eluted with a gradient system of EtOAc-hexane (0:1–1:0 v/v) and MeOH-EtOAc (0:1–1:0 v/v) to give 8 fractions, YB_1_-YB_8_. Fraction YB_2_ was purified by FCC eluted with 1:4 EtOAc-hexane to get a further 13 fractions, YB_2.1_-YB_2.13_. Three compounds, **17** (18.2 mg, 0.227%), **18** (194.2 mg, 0.366%) and **20** (71.6 mg, 0.135%) were obtained from fractions YB_2.13_, YB_2.10_ and YB_2.4_, respectively. Fraction YB_2.9_ was further purified by FCC eluted with 0.5:1:8.5 MeOH-EtOAc-hexane to give compound **16** (8.5 mg, 0.016%). Fraction YB_4_ was purified by CC eluted with a gradient system of EtOAc: hexane (1:4–1:0) to give 8 fractions, YB4.1-YB4.8. Fraction YB_4.5_ was separated by CC eluted with 2:3 EtOAc-hexane to yield compound **15** (21.3 mg, 0.040%).

The crude EtOAc extract from PDB broth (5.15 g) was purified by silica gel CC eluted with a gradient system of EtOAc-hexane (0:1–1:0 v/v) and MeOH-EtOAc (0:1–1:0 v/v) to give 8 fractions, PB_1_-PB_8_. Fraction PB_2_ was separated by CC eluted with 1:4 EtOAc-hexane to give compound **17** (29.2 mg, 0.015%,), **18** (518.1 mg, 0.269%,) and **20** (7.3 mg, 0.004%,) from fractions PB_2.7_, PB_2.5_ and PB_2.2_, respectively.

### Spectroscopic data of compounds

#### 1,3,8-Trihydroxy-7-methoxy-9-methyldibenzofuran (1)

Brown amorphous solid (MeOH); m.p. 244–245 °C; UV (MeOH) *λ*_max_ (log *ε*) 245 (4.23) nm; IR (ATR) *ν*_max_ 3354, 2929, 2851, 1615, 1462, 1430, 1350, 1152, 1131, 1090, 1064 cm^−1^; ^1^H and ^13^C NMR data, see Table 1; HRESIMS *m/z*: 283.0597 [M + Na]^+^ (calcd. for C_14_H_12_O_5_Na^+^, 283.0577).

#### (3*R*)-6-Methoxy-5-methoxycarbonylmellein (2)

White amorphous solid; m.p. 135–136 °C; [α]_D_^25.6^ -123.6 (c 1.0, CHCl_3_); UV (CH_2_Cl_2_) *λ*_max_ (log *ε*) 238 (4.22) nm; ECD (MeOH) *λ*(∆*ε*) 204 (+ 4.52) and 251 (-11.70) nm; IR (ATR) *ν*_max_ 2960, 2840, 1713, 1669, 1589, 1478, 1433, 1394, 1272, 1226, 1192, 1164 cm^−1^; ^1^H and ^13^C NMR data, see Table 1; HRESIMS *m/z*: 289.0687 [M + Na]^+^ (calcd. for C_13_H_14_O_6_Na^+^, 289.0683).

#### (3*S*,2′*R*,6′*R*)-Asperentin-8-*O*-methylether (15)

Yellow viscous liquid; [α]_D_^27.5^ + 11.4 (*c* 1.0, CH_2_Cl_2_); UV (MeOH) *λ*_max_ (log *ε*) 228 (4.36) nm; ECD (MeOH) *λ* (∆*ε*) 225 (+ 5.19), 250 (+ 0.42), 269 (+ 4.10) nm; IR (ATR) *ν*_max_ 3242, 2931, 2853, 1689, 1609, 1589, 1470, 1440, 1350, 1243, 1168, 1088 cm^−1^; ^1^H and ^13^C NMR data, see Table 2; HRESIMS *m/z*: 307.1546 [M + H]^+^ (calcd. for C_17_H_23_O_5_^+^, 307.1540).

#### (6*S*,2′*R*,6′*S*)-6-Methyl-2-((6-methyltetrahydro-2*H*-pyran-2-yl)methyl)-2,3-dihydro-4*H*-pyran-4-one (16)

Brown viscous liquid; [α]_D_^27.6^ -25.6 (*c* 1.0, CH_2_Cl_2_); UV (MeOH) *λ*_max_ (log *ε*) 260 (3.94) nm; ECD (MeOH) *λ* (∆*ε*) 213 (+ 0.86), 217 (+ 1.05), 259 (-1.46), 289 (-0.36), 314 (-0.89) nm; IR (ATR) *ν*_max_ 2929, 2867, 1666, 1610, 1462, 1438, 1400, 1337, 1114, 1086, 1034 cm^−1^; ^1^H and ^13^C NMR data, see Table 2; HRESIMS *m/z*: 247.1304 [M + Na]^+^ (calcd. for C_13_H_20_O_3_Na^+^, 247.1305).

#### (2′*R*,6′*S*)-5-((-6-Methyltetrahydro-2*H*-pyran-2-yl)methyl)benzene-1,3-diol (17)

Brown viscous liquid; [α]_D_^27.8^ -38.4 (*c* 0.1, EtOH); UV (MeOH) *λ*_max_ (log *ε*) 226 (3.06) nm; ECD (MeOH) *λ* (∆*ε*) 213 (-3.82), 228 (+ 0.76) nm; IR (ATR) *ν*_max_ 3269, 2971, 2937, 2869, 1601, 1453, 1481, 1339, 1147, 1037, 1004 cm^−1^; ^1^H and ^13^C NMR data, see Table 2; HRESIMS *m/z*: 223.1320 [M + H]^+^ (calcd. for C_13_H_19_O_3_^+^, 223.1329).

### Methylation of compound 1

Compound **1** was methylated using TMS-diazomethane (TCI, Japan) to yield the methylated product **1a**. Compound **1** (1.0 mg, 3.84 *µ*M) was dissolved in MeOH. TMS-diazomethane (200 μL) was then added to the solution and kept at room temperature overnight. The complete structure of **1** was deduced by the analysis of NMR spectroscopic data and the NOE experiment of the methylated product (**1a**).

### Quantitative high-performance liquid chromatography analysis (HPLC analysis)

The chemical profiling by HPLC was performed using Agilent ZORBAX Eclipse C18 (4.6 × 250 mm, 5 µm) column. Data processing was performed using OpenLAB CDS Chemstation software. Gradient elution was achieved with a mobile phase consisting of methanol and DI water, starting at a ratio of 60:40 v/v and increasing up to 100:0 v/v in 50 min at a flow rate of 1.0 mL/min. The injection volume was 10 μL and the mobile phase was filtrated and degassed by sonication before being used. The UV spectra were monitored over a range of 200 to 400 nm, while the chromatograms were recorded at 220 nm to detect all analyzed compounds. All crude extracts and pure compound solutions were prepared by adding HPLC grade methanol and then filtered through a 0.45 µm Nylon membrane. The concentration of crude extract solutions was 5 mg/mL and the chromatograms were identified by comparing their retention times and UV spectra with compounds **18** and **20**. Seven different concentrations of **18** and **20** were prepared to generate the calibration curves, which were performed by a two-fold dilution with an initial concentration of 1000 µg*/*mL to 15.6 µg*/*mL in triplicates. The validation of analytical methods was determined by linearity range, precision, detection and quantification limits. The linearity was determined by the correlation coefficients of the calibration curves. The limits of detection (LOD) and quantification (LOQ) were calculated based on the standard deviation and slope of the calibration curve. The precision was determined by repeatability (intraday assay) and intermediate precision (inter-day assay). The repeatability was measured by injecting three selected different concentrations at low (125 µg*/*mL), middle (250 µg*/*mL) and high (500 µg*/*mL) concentration of **18** and **20** in triplicate on the same day. Intermediate precision was estimated by analyzing the same solutions in triplicate over a period of three consecutive days. The accuracy of the method was expressed as % recovery. The recoveries of standard solutions at 125, 250 and 500 µg/mL were calculated from the corresponding calibration curve [40].

### Quantum chemical calculations

The quantum chemical calculations including ^13^C NMR chemical shift and Electronic Circular Dichroism (ECD) calculations were used to determine the absolute configurations. Conformational searches were carried out by Monte Carlo protocol under MM + molecular mechanics force field by HyperChem *(*HyperChem™ Professional 8.0.4, Hypercube, Inc., Gainesville, FL, USA). Re-optimization of the stable conformers with a Boltzmann distribution over 5% were performed using the density functional theory (DFT) with the B3LYP hybrid functional and 6-31G(d) basis sets [17, 41]. GaussView 6.0 was used to view the optimized structures and modify computation input files for calculation. For ^13^C NMR calculation, the magnetic shielding tensors of each optimized conformer were calculated using GIAO method at B3LYP functional and 6-31g(d,p) basis set. The solvent effects were analyzed using the Conductor–like Polarisable Continuum Model (CPCM) in chloroform. GaussView 6.0 was used to display the calculated shielding tensors of each nuclei in each conformer. The Boltzmann averaged shielding tensors of each carbon atom were calculated and then input to an Excel file (downloaded from sarotti-nmr.weebly.com) for the DP4 + (C data) probability analysis*.* For ECD calculation, the transition energies for the valence excited–states and absorption ECD spectra were computed with the time-dependent density functional theory (TD–DFT) method with the CAM–B3LYP long–range corrected functional at the 6–311 +  + G(d,p) basis set. The analysis of solvent effects was estimated using C–PCM in DCM. The ECD calculations were performed using the Gaussian 09 program, and calculated ECD spectra were generated using the GaussSum program [42].

### Antiproliferative activity against cancer cell lines

The antiproliferative activity was evaluated by MTT (3-(4,5-dimethylthiazol-2-yl)-2,5-diphenyl tetrazolium bromide) assay [43]. Four cancer cell lines; human cervical carcinoma (HeLa), human colon carcinoma (HT29 and HCT116) and human breast carcinoma (MCF-7), along with African green monkey kidney cells (Vero cells) were seeded in 96-well plates (8000 cells/well) and cultured for 24 h, followed by being treated with the selected compounds at 100 µg/mL for 72 h. Following this, 10 *μ*L of MTT reagent (5 mg/mL in phosphate buffer solution (PBS)) was added to each well and further incubated at 37 °C for 2 h. Then, 100 *μ*L of DMSO (dimethyl sulfoxide) was used to dissolve the formazan dye. Finally, the absorbance of the solution was measured using a microplate reader (Bio-Rad Laboratories, Hercules, CA) at wavelengths of 550 and 655 nm. The absorbance at 655 nm was used as a reference wavelength. Compounds that showed strong anti-proliferative activity in the preliminary test, with less than 10% viability, were further determined for the half maximum inhibitory concentration (IC_50_) values using the same procedures. Cisplatin was used as the positive control drug.

### Supplementary Information

**Additional file 1. **Separation of known compounds (3–14 and 18–28). Spectroscopic data of all isolated compounds. The ^13^C NMR chemical shift calculations and ECD spectra of 15–17. HPLC identification of asperentin (18) and (3*R*,2'*R*,6'*S*)-asperentin-6-*O*-methylether (20) and method validation.
