# Supplementary material for: Antiproliferative polyketides from fungus Xylaria cf. Longipes SWUF08-81 in different culture media
Source: Nat Prod Bioprospect. 2024 Jan 6;14(1):6. doi: 10.1007/s13659-023-00427-7 (PMC10770013; doi:10.1007/s13659-023-00427-7)
Supplement: Supplementary file 1 — Additional file 1. Separation of known compounds (3–14 and 18–28). Spectroscopic data of all isolated compounds. The 13C NMR chemical shift calculations and ECD spectra of 15–17. HPLC identification of asperentin (18) and (3R,2'R,6'S)-asperentin-6-O-methylether (20) and method validation. [file 13659_2023_427_MOESM1_ESM.pdf]

SUPPLEMENTARY INFORMATION

Antiproliferative Polyketides from Fungus *Xylaria* cf. *Longipes* SWUF08-81 in Different Culture Media

Kittiwan Sresuksai[a], Sasiphimol Sawadsitang[a], Phongphan Jantaharn[a], Pakin Noppawan[b], Audomsak Churat[a], Nuttika Suwannasai[c], Wiyada Mongkolthanaruk[d], Thanaset Senawong[e], Sarawut Tontapha[f], Pairot Moontragoon[fg], Vittaya Amornkitbamrung[f,g], Sirirath McCloskey[a,*]

Affiliation

[a] *Center of Excellence for Innovation in Chemistry (PERCH-CIC), Department of Chemistry, Faculty of Science, Khon Kaen University, Khon Kaen 40002, Thailand.*

[b] *Department of Chemistry, Faculty of Science, Mahasarakham University, Maha Sarakham 44150, Thailand.*

[c] *Department of Microbiology, Faculty of Science, Srinakharinwirot University, Bangkok 10110, Thailand.*

[d] *Department of Microbiology, Faculty of Science, Khon Kaen University, Khon Kaen 40002, Thailand.*

[e] *Department of Biochemistry, Faculty of Science, Khon Kaen University, Khon Kaen 40002, Thailand.*

[f] *Department of Physics, Faculty of Science, Khon Kaen University, Khon Kaen 40002, Thailand.*

[g] *Institute of Nanomaterials Research and Innovation for Energy (IN-RIE), Khon Kaen University, Khon Kaen 40002, Thailand.*

Corresponding authors:

[*] Sirirath McCloskey

Center of Excellence for Innovation in Chemistry (PERCH-CIC), Department of Chemistry, Faculty of Science, Khon Kaen University, Khon Kaen 40002, Thailand,

E-mail: sirsod@kku.ac.th

Contents

Page

Isolation process of crude extracts from *X*. cf. *longipes* SWUF08-81 10

1D and 2D NMR, IR, ECD and mass spectroscopic data of isolated compounds............13

HPLC identification and quantification of asperentin (18) and (3*R*,2′*R*,6′*S*)-asperentin-6-*O*-methylether (20) 99

1. Identification of compounds 18 and 20 99

2. Method validation 100

2.1. Calibration curve and linearity...100

2.2. LOD and LOQ 101

2.3. Intra and inter-day assay precisions and accuracy...................................101

References...103

List of Tables

Page

Table S1 ^{1}H, ^{13}C, 2D NMR and NOE data of compound **1** (500 MHz, CD_3OD)....13
Table S2 ^{1}H and ^{13}C NMR data of compound **1** (500 MHz, CD_3OD) and methylated **1a** (500 MHz, $CDCl_3$) together with 2D NMR and NOE data of **1a**....14
Table S3 ^{1}H, ^{13}C, 2D NMR and NOE data of compound **2** (400 MHz, $CDCl_3$)....24
Table S4 ^{1}H and ^{13}C NMR data of compound **3** (500 MHz, $CDCl_3$), (-)-mellein (600 MHz, $CDCl_3$) and (+)-mellein (400 MHz, $CDCl_3$)....31
Table S5 ^{1}H and ^{13}C NMR spectral data of compound **4** (500 MHz, $CDCl_3$) and (-)-5-methoxycarbonylmellein (300 MHz, $CDCl_3$) 32
Table S6 ^{1}H and ^{13}C NMR data of compound **5** (400 MHz, $CDCl_3$ and CD_3OD, 2/1 v/v) and (-)-5-carboxymellein (300 MHz, $CDCl_3$ and CD_3OD)....33
Table S7 ^{1}H and ^{13}C NMR data of compound **6** (400 MHz, $CDCl_3$) and cytochalasin D (300 MHz, $CDCl_3$) 34
Table S8 ^{1}H and ^{13}C NMR data of compound **7** (400 MHz, $CDCl_3$) and zygosporin D (400 MHz, $CDCl_3$) 35
Table S9 ^{1}H and ^{13}C NMR data of compound **8** (400 MHz, $CDCl_3$) and 19,20-epoxycytochalasin D (400 MHz, $CDCl_3$) 36
Table S10 ^{1}H and ^{13}C NMR data of compound **9** (400 MHz, CDCl3) and cytochalasin Ohyp (^{1}H NMR 500 MHz, $CDCl_3$ and ^{13}C NMR 400 MHz, pyridine-d_5) 37
Table S11 ^{1}H and ^{13}C NMR data of compound **10** (400 MHz, pyridine-d_5) and cytochalasin C (400 MHz, pyridine-d_5) 38
Table S12 ^{1}H and ^{13}C NMR data of compound **11** (400 MHz, $CDCl_3$) and 2-chloro-5-methoxy-3-methylcyclohexa-2,5-diene-1,4-dione (400 MHz, $CDCl_3$)....39
Table S13 ^{1}H and ^{13}C NMR data of compound **12** (500 MHz, $CDCl_3$) and 2-hydroxy-5-methoxy-3-methylcyclohexa-2,5-diene-1,4-dione (400 MHz, $CDCl_3$) 39
Table S14 ^{1}H and ^{13}C NMR spectral data of compound **13** (400 MHz, CD3OD) and 4-hydroxymethylbenzoate (500 MHz, DMSO-d_6)....40

Table S15 ^{1}H and ^{13}C NMR data of compound **14** (400 MHz, acetone-d_6) and (4R,5S,6R)-4,5,6-trihydroxy-3-methoxy-5-methyl-cyclohex-2-en-1-one (400 MHz, acetone-d_6) 40

Table S16 ^{1}H, ^{13}C, 2D NMR and NOE data of compound **15** (500 MHz, $CDCl_3$)....................41

Table S17 Experimental ^{13}C NMR chemical shifts of **15** and the calculated Boltzmann averaged shielding values of **15a**, **15c**, **15e**, and **15g** diastereomers 50

Table S 18 The results of energy analysis for conformers of **15aa-ac** 52

Table S19 Cartesian coordinates for the low-energy optimized conformers of **15aa-ac**....................53

Table S20 ^{1}H, ^{13}C, 2D NMR and NOE data of compound **16** (500 MHz, $CDCl_3$)....................58

Table S21 Experimental ^{13}C NMR chemical shifts of **16** and Boltzmann averaged shielding values of **16a**, **16c**, **16e**, and **16g** diastereomers....................65

Table S22 The results of Energy analysis for conformers of **16aa-ab** 67

Table S23 Cartesian coordinates for the low-energy optimized conformers of **16a**....................68

Table S24 ^{1}H, ^{13}C, 2D NMR and NOE data of compound **17** (500 MHz, $CDCl_3$), and ^{1}H and ^{13}C NMR data of aspyran (500 MHz, $CDCl_3$)[11]....................72

Table S 25 Experimental ^{13}C NMR chemical shifts of **17** and Boltzmann averaged shielding values of **17a** and **17c** diastereomers....................80

Table S26 The results of energy analysis for conformers of **17aa-ae** 81

Table S27 Cartesian coordinates for the low-energy optimized conformers of **17a**....................82

Table S28 ^{1}H and ^{13}C NMR data of compound **18** (500 MHz, CD_3OD) and asperentin (500 MHz, CD_3OD), isocladosporin (400 MHz, $CDCl_3$) and 3-epi-isocladosporin (400 MHz, CD_3OD) 87

Table S29 ^{1}H and ^{13}C NMR data of compound **19** (500 MHz, DMSO-d_6) and asperentin-8-O-methylether (DMSO-d_6) 88

Table S30 ^{1}H, ^{13}C and NOE NMR data of compound **20** (500 MHz, $CDCl_3$) and asperentin-6-O-methylether (500 MHz, $CDCl_3$)....................89

Table S31 ^{1}H and ^{13}C NMR data of compound **21** (500 MHz, CD_3OD) and 5′-hydroxyasperentin (500 MHz, CD_3OD) 90

Table S32 ^{1}H and ^{13}C NMR data of compound **22** (500 MHz, CD_3OD) and 4′-hydroxyasperentin (500 MHz, CD_3OD) 91

Table S33 ^{1}H and ^{13}C NMR data of compound **23** (400 MHz, $CDCl_3$) and tetrahydroauroglaucin (500 MHz, $CDCl_3$)........92
Table S34 ^{1}H and ^{13}C NMR data of compound **24** (400 MHz, $CDCl_3$) and flavoglaucin (500 MHz, $CDCl_3$)........93
Table S35 ^{1}H and ^{13}C NMR data of compound **25** (400 MHz, $CDCl_3$) and auroglaucin (500 MHz, Acetone-d_6)........94
Table S36 ^{1}H and ^{13}C NMR data of compound **26** (400 MHz, $CDCl_3$) and isodihydroauroglaucin (500 MHz, $CDCl_3$)........95
Table S37 ^{1}H and ^{13}C NMR data of compound **27** (400 MHz, $CDCl_3$ and CD_3OD, 4/1 v/v), and ^{13}C NMR data of echinulin (500 MHz, $CDCl_3$)........96
Table S38 ^{1}H and ^{13}C NMR data of compound **28** (500 MHz, $CDCl_3$ and CD_3OD, 4/1 v/v) and physcion (400 MHz, $CDCl_3$)........97
Table S39 The melting point and specific rotation of all isolated compounds compared with their literatures........98
Table S40 Repeatability and intermediate precision data of compounds **18** and **20**........101
Table S41 Weight of crude extracts from X. longipes SWUF08-81........102

List of Figures

Page

Figure S1 Xylaria cf. longipes SWUF08-81 (**A**) stroma, (**B**) fungal culture on potato dextrose agar (PDA)........10
Figure S2 Structure of 1,3,8-trihydroxy-7-methoxy-9-methyldibenzofuran (**1**)........13
Figure S3 Methylation of compound **1** to **1a**........14
Figure S4 NOE correlation of compound **1** and methylated **1a**........14
Figure S5 ^{1}H NMR spectrum of **1** (500 MHz, CD_3OD)........15
Figure S6 ^{13}C NMR spectrum of **1** (125 MHz, CD_3OD)........15
Figure S7 DEPT135 spectrum of **1**........16
Figure S8 HSQC spectrum of **1**........16
Figure S9 HMBC spectrum of **1**........17
Figure S10 NOE (C-7-OCH_3) spectrum of **1**........17
Figure S11 NOE (H-6) spectrum of **1**........18
Figure S12 IR spectrum of **1**........18
Figure S13 HRESIMS spectrum of **1**........19
Figure S14 ^{1}H NMR spectrum of **1a** (500 MHz, $CDCl_3$)........19
Figure S15 ^{13}C NMR spectrum of **1a** (125 MHz, $CDCl_3$)........20
Figure S16 HSQC spectrum of **1a**........20
Figure S17 HMBC spectrum of **1a**........21
Figure S18 NOE (C-1-OCH_3) spectrum of **1a**........21
Figure S19 NOE (C-3-OCH_3) spectrum of **1a**........22
Figure S20 NOE (C-7-OCH_3) spectrum of **1a**........22
Figure S21 NOE (C-8-OCH_3) spectrum of **1a**........23
Figure S22 IR spectrum of compound **1a**........23
Figure S23 HRESIMS spectrum of **1a**........24
Figure S24 Structure of (3R)-7-methoxy-5-methoxycarbonylmellein (**2**)........24
Figure S25 ^{1}H NMR spectrum of **2** (400 MHz, $CDCl_3$)........25
Figure S26 ^{13}C NMR spectrum of **2** (100 MHz, $CDCl_3$)........25
Figure S27 DEPT spectrum of **2**........26
Figure S28 HMQC spectrum of **2**........26
Figure S29 COSY spectrum of **2**........27
Figure S30 HMBC spectrum of **2**........27

Figure S31 NOE (C-3-CH_3) spectrum of **2** 28
Figure S32 NOE (C-7-OCH_3) spectrum of **2** 28
Figure S33 IR spectrum of **2** 29
Figure S34 HRESIMS spectrum of **2** 29
Figure S35 Experimental and calculated ECD spectra of **2** 30
Figure S36 Structures of 3R and 3S isomers of **2** 30
Figure S37. Structures of (-)-mellein (**3**) and (+)-mellein 31
Figure S38. Structure of (-)-5-methoxycarbonylmellein (**4**) 32
Figure S39. Structure of (-)-5-carboxymellein (**5**) 33
Figure S40. Structure of cytochalasin D (**6**) 34
Figure S41 Structure of zygosporin D (**7**) 35
Figure S42 Structure of 19,20-epoxycytochalasin D (**8**) 36
Figure S43 Structure of cytochalasin O_{hyp} (**9**) 37
Figure S44 Structure of cytochalasin C (**10**) 38
Figure S45 Structure of 2-chloro-5-methoxy-3-methylcyclohexa-2,5-diene-1,4-dione (**11**) 39
Figure S46 Structure of 2-hydroxy-5-methoxy-3-methylcyclohexa-2,5-diene-1,4-dione (**12**) 39
Figure S47. Structure of 4-hydroxymethylbenzoate (**13**) 40
Figure S48 Structure of (4R,5S,6R)-4,5,6-trihydroxy-3-methoxy-5-methyl-cyclohex-2-n-1-one (**14**) 40
Figure S49 Structure of (3R,2′R,6′R)-asperentin-8-O-methylether (**15**) 41
Figure S50 ^{1}H NMR spectrum of **15** (500 MHz, $CDCl_3$) 42
Figure S51 ^{13}C NMR spectrum of **15** (125 MHz, $CDCl_3$) 43
Figure S52 DEPT135 spectrum of **15** 44
Figure S53 HSQC spectrum of **15** 45
Figure S54 COSY spectrum of **15** 46
Figure S55 HMBC spectrum of **15** 47
Figure S56 NOE (H-2′) spectrum of **15** 48
Figure S57 NOE (H-6′) spectrum of **15** 48
Figure S58 NOE (C-6′-CH_3) spectrum of **15** 49
Figure S59 NOE correlations of compounds **15** 49

Figure S60 Structures of **15a**, **15c**, **15e** and **15g** diastereomers used for ^{13}C NMR chemical shift calculations........49
Figure S61 Linear correlations between unscaled and experimental ^{13}C NMR chemical shifts of **15a**, **15c**, **15e**, and **15g** diastereomers........51
Figure S62 The results of DP4+ analysis of **15a**, **15c**, **15e**, and **15g** diastereomers........52
Figure S63 Comparison of experimental and calculated ECD spectra of **15**........56
Figure S64 IR spectrum of **15**........57
Figure S65 HRESIMS spectrum of **15**........57
Figure S66 Structure of (6*S*,2′*R*,6′*S*)-6-methyl-2-((6-methyltetrahydro-2*H*-pyran-2-yl)methyl)-2,3-dihydro-4*H*-pyran-4-one (**16**)........58
Figure S67 ^{1}H NMR spectrum of **16** (500 MHz, $CDCl_3$)........59
Figure S68 ^{13}C NMR spectrum of **16** (125 MHz, $CDCl_3$)........60
Figure S69 DEPT135 spectrum of **16**........61
Figure S70 HSQC spectrum of **16**........62
Figure S71 COSY spectrum of **16**........63
Figure S72 HMBC spectrum of **16**........64
Figure S73 Structures of **16a**, **16c**, **16e** and **16g** diastereomers used for ^{13}C NMR chemical shift calculations........65
Figure S74 Linear correlations between unscaled and experimental ^{13}C NMR chemical shifts of **16a**, **16c**, **16e**, and **16g** diastereomers........66
Figure S75 The results of DP4+ analysis of **16a**, **16c**, **16e**, and **16g** diastereomers........67
Figure S76 Comparison of experimental and calculated ECD spectra of **16**........70
Figure S77 IR spectrum of **16**........71
Figure S78 HRESIMS spectrum of **16**........71
Figure S79 Structures of (2′*R*,6′*S*)-5-((-6-methyltetrahydro-2*H*-pyran-2-yl)methyl)benzene-1,3-diol (**17**) and its analog, aspyran........72
Figure S80 ^{1}H NMR spectrum of **17** (500 MHz, $CDCl_3$)........73
Figure S81 ^{13}C NMR spectrum of **17** (125 MHz, $CDCl_3$)........74
Figure S82 DEPT135 spectrum of **17**........75
Figure S83 HSQC spectrum of **17**........76
Figure S84 COSY spectrum of **17**........77

Figure S85 HMBC spectrum of **17** 78
Figure S86 IR spectrum of **17** 79
Figure S87 HRESIMS spectrum of **17** 79
Figure S88 Structures of **17a** and **17c** diastereomers used for ^{13}C NMR chemical shift calculations 79
Figure S89 Linear correlations between unscaled and experimental ^{13}C NMR chemical shifts of **17a** and **17c** diastereomers 80
Figure S90 The results of DP4+ analysis of **17a** and **17c** diastereomers 81
Figure S91 Comparison of experimental and calculated ECD spectra of **17** 86
Figure S92 Structures of asperentin (**18**) and its diastereomers, isocladosporin and 3-*epi*-isocladosporin 87
Figure S93 Structure of asperentin-8-*O*-methylether (**19**) 88
Figure S94 Structure of asperentin-6-*O*-methylether (**20**) 89
Figure S95 Structure of 5′-hydroxyasperentin (**21**) 90
Figure S96 Structure of 4′-hydroxyasperentin (**22**) 91
Figure S97 Structure of tetrahydroauroglaucin (**23**) 92
Figure S98 Structure of flavoglaucin (**24**) 93
Figure S99 Structure of auroglaucin (**25**) 94
Figure S100 Structure of isodihydroauroglaucin (**26**) 95
Figure S101 Structure of echinulin (**27**) 96
Figure S102 Structure of physcion (**28**) 97
Figure S 103 (A) HPLC chromatogram and (**B**) UV spectrum of all crude extracts and compound **18** 99
Figure S104 (**A**) HPLC chromatogram and (**B**) UV spectrum of all crude extracts and compound **20** 100
Figure S105 Calibration curves of compounds **18** and **20** 101

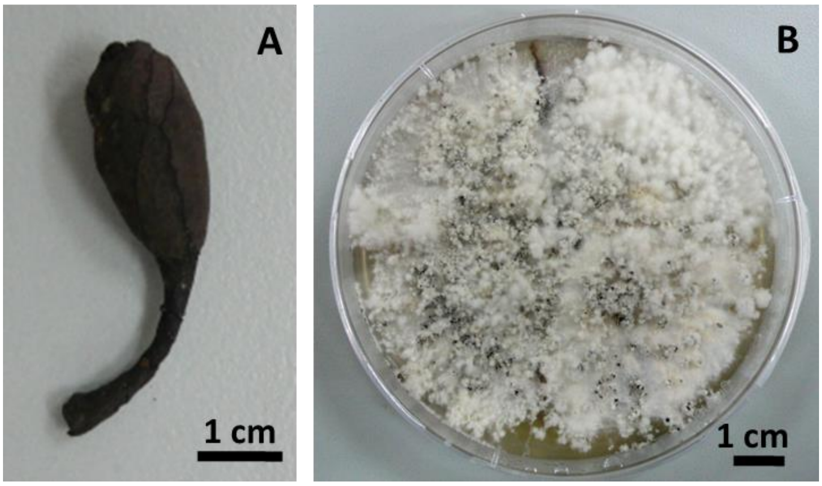

Figure S1 *Xylaria* cf. *longipes* SWUF08-81 (**A**) stroma, (**B**) fungal culture on potato dextrose agar (PDA)

Isolation process of crude extracts from *X*. cf. *longipes* SWUF08-81

The crude EtOAc extract of GM broth (128.15 g) was purified by silica gel CC eluted with a gradient system of EtOAc-hexane (0:1-1:0 v/v) and MeOH-EtOAc (0:1-1:0 v/v) to give 10 fractions, GB_1-GB_{10}. Fraction GB_3 was purified by FCC eluted with a gradient system of EtOAc-hexane (0.5:9.5-1:0 v/v) to get 14 fractions, $GB_{3.1}$-$GB_{3.14}$. Fraction $GB_{3.3}$ was purified on PLC eluted with CH_2Cl_2-hexane (7:3 v/v) to yield **3** (31.8 mg, 0.004%) and **4** (23.3 mg, 0.003%). Five fractions $GB_{3.5.1}$-$GB_{3.5.5}$ were obtained when $GB_{3.5}$ was subjected to FCC eluted with CH_2Cl_2-hexane (7:3 v/v). $GB_{3.5.1}$ was further purified on PLC eluted with MeOH-CH_2Cl-hexane (1:7:2 v/v) to obtain **11** (45.5 mg, 0.005%). The purification of $GB_{3.5.5}$ on FCC eluted with MeOH-CH_2Cl_2-hexane (0.5:5:4.5 v/v) afforded **12** (61.9 mg, 0.007%). Fraction $GB_{3.7}$ was separated by FCC eluted with EtOAc-hexane (2:3 v/v) to give **2** (109.3 mg, 0.012%) in $GB_{3.7.4}$. Further separation of $GB_{3.11}$ by FCC eluted with EtOAc-CH_2Cl_2 (3:2 v/v) gave **5** (119.2 mg, 0.014%). Fractions $GB_{3.12.1}$-$EBF_{3.12.4}$ were obtained when fractionation of $GB_{3.12}$ by FCC eluted with EtOAc-CH_2Cl_2 (3:2 v/v). Fraction $GB_{3.12.1}$ was purified by PLC developed with MeOH-CH_2Cl_2-hexane (1:7:2 v/v) to yield **1** (13.9 mg, 0.002%). Fraction GB_5 was subjected to FCC eluted with a system of EtOAc-CH_2Cl_2-hexane (4:1:5 v/v) to get 8 fractions, $GB_{5.1}$-$GB_{5.8}$. Fraction $GB_{5.7}$ was separated by FCC eluted with MeOH-CH_2Cl_2-hexane (1:5:4 v/v) to get 8 fractions, $GB_{5.7.1}$-$GB_{5.7.8}$. The further separation of $GB_{5.7.1}$ by FCC eluted with acetone-hexane (3:7 v/v) afforded **6** (457.6 mg, 0.052%). Fraction $GB_{5.7.3}$ was purified by FCC eluted with acetone-CH_2Cl_2-hexane (3:5:2 v/v) to yield **7** (73.8

mg, 0.009%) in $GB_{5.7.3.2}$. Fraction $GB_{5.7.6}$ was purified by FCC eluted with MeOH-CH_2Cl_2-hexane (2:7:1 v/v) to afford **13** (5.8 mg, 0.001%). Fraction GB_7 was separated by FCC eluted with EtOAc-CH_2Cl_2-hexane (4:1:5 v/v) to afford 7 fractions, $GB_{7.1}$-$GB_{7.7}$. Fraction $GB_{7.5}$ was then subjected to FCC eluted with MeOH-EtOAc-hexane (0.5:0.5:9 v/v) to get 5 fractions, $GB_{7.5.1}$-$GB_{7.5.5}$. The further purification of $GB_{7.5.2}$ by FCC eluted with EtOAc-CH_2Cl_2-hexane (7:1:2 v/v) yielded fractions $GB_{7.5.2.1}$-$GB_{7.5.2.5}$. Compound **8** (36.7 mg, 0.004%) was obtained from $GB_{7.5.2.3.4}$, when separation of $GB_{7.5.2.3}$ on FCC eluted with MeOH-CH_2Cl_2-hexane (0.5:6:3.5 v/v). Fraction $GB_{7.6}$ was further subjected to FCC eluted with acetone-hexane (2:3 v/v) to afford 6 fractions, $GB_{7.6.1}$-$GB_{7.6.6}$. Fraction $GB_{7.6.1}$ was then separated on FCC eluted with acetone-hexane (2:3 v/v) to get 5 fractions, $GB_{7.6.1.1}$-$GB_{7.6.1.5}$. The further purification of $GB_{7.6.1.4}$ by FCC eluted with acetone-CH_2Cl_2-hexane (3:5:2 v/v) gave **9** (14.4 mg, 0.002%).

The crude EtOAc extract of GM mycelium (45.44 g) was purified by silica gel CC eluted with a gradient system of EtOAc-hexane (0:1-1:0 v/v) and MeOH-EtOAc (0:1-1:0 v/v) to give 7 fractions, GE_1-GE_7. Fraction GE_3 was purified on FCC eluted with CH_2Cl_2-hexane (3:2 v/v) to give 8 fractions, $GE_{3.1}$-$GE_{3.8}$. Fraction $GE_{3.4}$ was re-purified by FCC eluted with CH_2Cl_2-hexane (3:2 v/v) to give additional of **2** (47.5 mg, 0.005%). Fraction $GE_{3.6}$ appeared as a mixture of white solid and yellow liquid, which was recrystallized by EtOAc-hexane (1:1 v/v) to yield **10** (30.2 mg, 0.004%). Fraction GE_5 was subjected to FCC eluted with EtOAc-hexane (5.5:4.5 v/v) to give 5 fractions, $GE_{5.1}$-$GE_{5.5}$. Fraction $GE_{5.5}$ was further subjected to FCC eluted with EtOAc-CH_2Cl_2-hexane (7:2:1 v/v) to give 4 fractions, $GE_{5.5.1}$-$GE_{5.5.4}$. Fraction $GE_{5.5.4}$ was fractionated on FCC eluted with EtOAc-CH_2Cl_2-hexane (5:4:1 v/v) to afford 3 fractions, $GE_{5.5.4.1}$-$GE_{5.5.4.3}$. Fraction $GE_{5.5.4.2}$ was purified on PLC developed with EtOAc-CH_2Cl_2-hexane (6:3:1 v/v) to give **14** (123.6 mg, 0.014%).

The crude EtOAc extract from YM broth (9.86 g) was purified by silica gel CC and eluted with a gradient system of EtOAc-hexane (0:1-1:0 v/v) and MeOH-EtOAc (0:1-1:0 v/v) to give 8 fractions, YB_1-YB_8. Fraction YB_2 was purified by FCC eluted with EtOAc-hexane (1:4 v/v) to get 13 subfractions, $YB_{2.1}$-$YB_{2.13}$. Three compounds **17** (18.2 mg, 0.227%), **18** (194.2 mg, 0.366%) and **20** (71.6 mg, 0.135%), were obtained from fractions $YB_{2.13}$, $YB_{2.10}$ and $YB_{2.4}$, respectively. Fraction $YB_{2.9}$ was further purified by FCC eluted with MeOH-EtOAc-hexane (0.5:1:8.5 v/v) to give **16** (8.5 mg, 0.016%). Fraction $YB_{4.5}$ was separated by CC eluted with EtOAc-hexane (2:3 v/v) to yield **15** (21.3 mg, 0.040%). The purification of YB_5 on FCC eluted with a gradient system of EtOAc-hexane (2:3-1:0 v/v) and MeOH-EtOAc (0:1-1:4 v/v) yielded 5 fractions, $YB_{5.1}$-$YB_{5.5}$. Fraction $YB_{5.1}$ was purified by FCC eluted with MeOH-EtOAc-hexane (1:2:7 v/v) to give **19** (1.3072 g, 2.464%). Compounds **21** (381.7

mg, 0.719%) and **22** (87.4 mg, 0.165%) were obtained from the fractionation of $YB_{5.2}$ by FCC eluted with EtOAc-hexane (2:3 v/v).

The crude EtOAc extract of YM mycelium (7.09 g) was purified by silica gel CC eluted with a gradient system of EtOAc-hexane (0:1-1:0 v/v) and MeOH-EtOAc (0:1-1:0 v/v) to give 7 fractions, YE_1-YE_7. The purification of fraction YE_2 was carried out by FCC eluted with EtOAc-CH_2Cl_2-hexane (0.2:0.8:9 v/v) to yield 8 fractions, $YE_{2.1}$-$YE_{2.8}$. Fraction $YE_{2.3}$ was purified by FCC eluted with CH_2Cl_2-hexane (1:9 v/v) to give **23** (375.8 mg, 0.708%) and **24** (257.4 mg, 0.485%). Compounds **25** (19.0 mg, 0.036%) and **26** (114.4 mg, 0.216%) were obtained when $YE_{2.5}$ was purified by FCC eluted with EtOAc-hexane (0.5:9.5 v/v). Fraction YE_5 was separated on FCC eluted with EtOAc-CH_2Cl_2 (1:4 v/v) to yield **27** (24.2 mg, 0.046%). The purification of YE_6 was carried out by Sephadex LH-20 CC eluted with MeOH to yield **28** (10.7 mg, 0.020%).

The crude EtOAc extract from PDB broth (5.15 g) was purified by silica gel CC eluted with a gradient system of EtOAc-hexane (0:1-1:0 v/v) and MeOH-EtOAc (0:1-1:0 v/v) to give 8 fractions, PB_1-PB_8. Fraction PB_2 was separate by CC eluted with EtOAc-hexane (2:8 v/v) to give compounds **17** (29.2 mg, 0.015%,) **18** (518.1 mg, 0.269%,) and **20** (7.3 mg, 0.004%,) from fractions $PB_{2.7}$, $PB_{2.5}$ and $PB_{2.2}$ respectively. Fraction PB_5 was separated by CC eluted with EtOAc-hexane (2:3 v/v) to give compound **19** (574.4 mg, 0.299%,) **22** (16.3 mg, 0.008%,) and **21** (179.3 mg, 0.093%,).

The crude EtOAc extract of PDB mycelium (3.93 g) was subject on silica gel CC using gradient elution with EtOAc-hexane (0:1-1:0 v/v) and MeOH-EtOAc (0:1-1:0 v/v) to afford 9 fractions, PE_1-PE_9. Fraction PE_3 was purified by FCC eluted with EtOAc-CH_2Cl_2-hexane (0.5:0.5:9 v/v) to afford compounds **24** (66.2 mg, 0.034%,) and **26** (2.7 mg, 0.001%,).

1D and 2D NMR, IR, ECD and mass spectroscopic data of isolated compounds

Table S1 ^{1}H, ^{13}C, 2D NMR and NOE data of compound **1** (500 MHz, CD_3OD)

**Position /DEPT**	δ_H	δ_C	**HMBC**	**NOE**
1 C		152.0		
2 CH	6.22 (d, 1.7)	97.0	C-1, 4, 4a, 9b	
3 C		159.1		
4 CH	6.40 (d, 1.7)	89.4	C-2, 3, 4a, 9b	
4a C		156.6		
5a C		149.1		
6 CH	6.92 (s)	91.7	C-5a, 7, 8, 9a	7-OCH_3
7 C		145.8		
8 C		140.1		
9 C		117.6		
9a C		116.5		
9b C		106.1		
7-OCH_3	3.91 (s)	55.3	C-7	H-6
9-CH_3	2.78 (s)	13.2	C-8, 9	

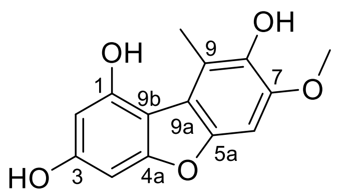

Figure S2 Structure of 1,3,8-trihydroxy-7-methoxy-9-methyldibenzofuran (**1**)

Table S2 ^{1}H and ^{13}C NMR data of compound **1** (500 MHz, CD_3OD) and methylated **1a** (500 MHz, $CDCl_3$) together with 2D NMR and NOE data of **1a**

**Position**	**1**		**Methylated 1a**			
	δ_H	δ_C	δ_H	δ_C	**HMBC**	**NOE**
1		152.0		154.3		
2	6.22 (d, 1.7)	97.0	6.38 (d, 2.0)	93.9		
3		159.1		159.6		
4	6.40 (d, 1.7)	89.4	6.67 (d, 2.0)	88.5		
4a		156.6		158.5		
5 O						
5a		149.1		152.1		
6	6.92 (s)	91.7	6.91 (s)	93.0	7, 8, 9a	
7		145.8		151.5		
8		140.1		143.9		
9		117.6		126.2		
9a		116.5		116.0		
9b		106.1		112.0		
1-OCH_3			3.95 (s)	55.3	1	2
3-OCH_3			3.88 (s)	55.7	3	4
7-OCH_3	3.91 (s)	55.3	3.92 (s)	56.0	7	6
8-OCH_3			3.80 (s)	60.7	8	9-CH_3
9-CH_3	2.78 (s)	13.2	2.80 (s)	14.6	8, 9, 9a	

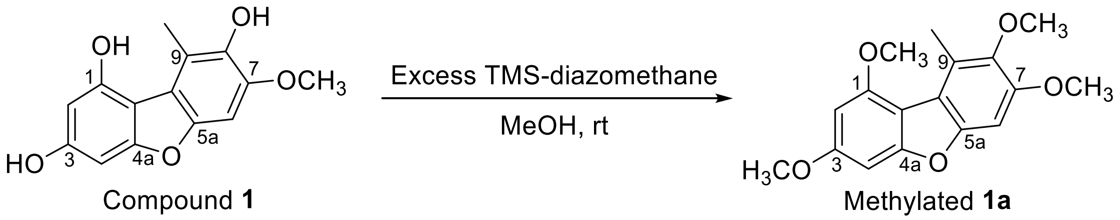

Figure S3 Methylation of compound **1** to **1a**

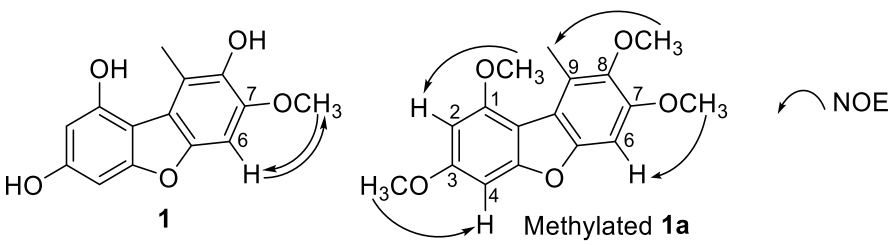

Figure S4 NOE correlation of compound **1** and methylated **1a**

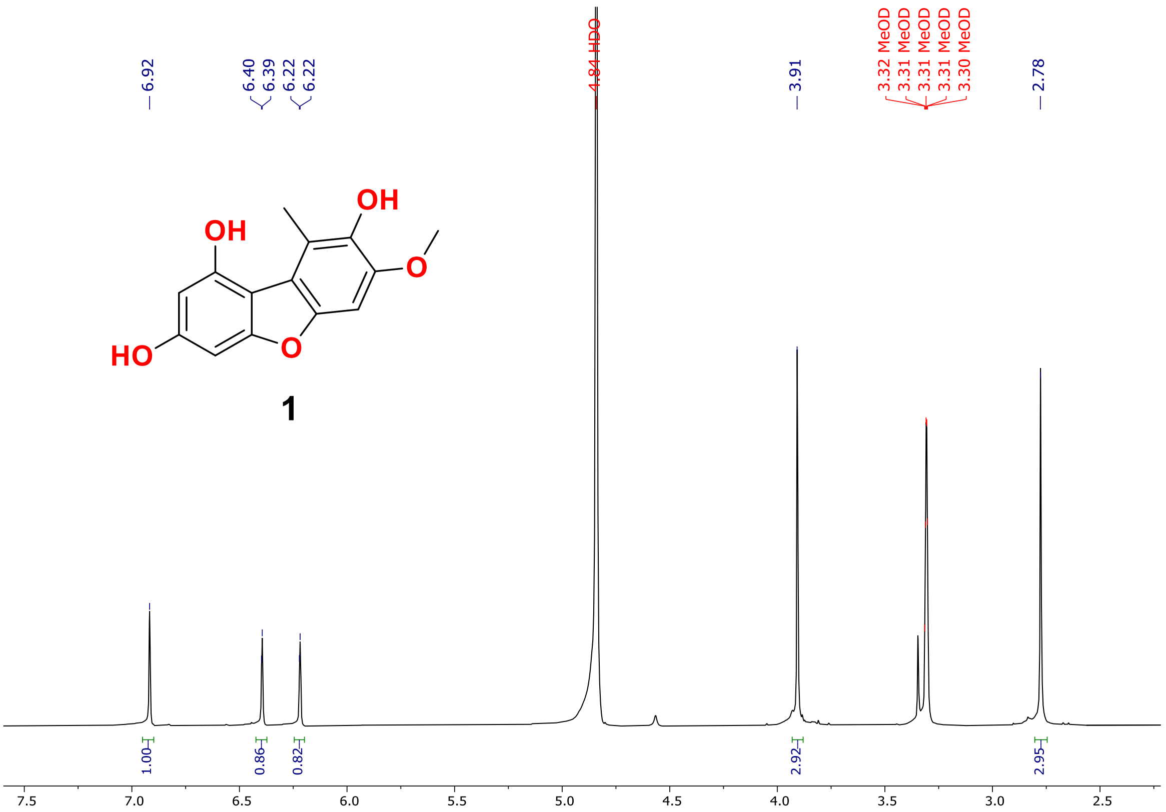

Figure S5 ^{1}H NMR spectrum of **1** (500 MHz, CD_3OD)

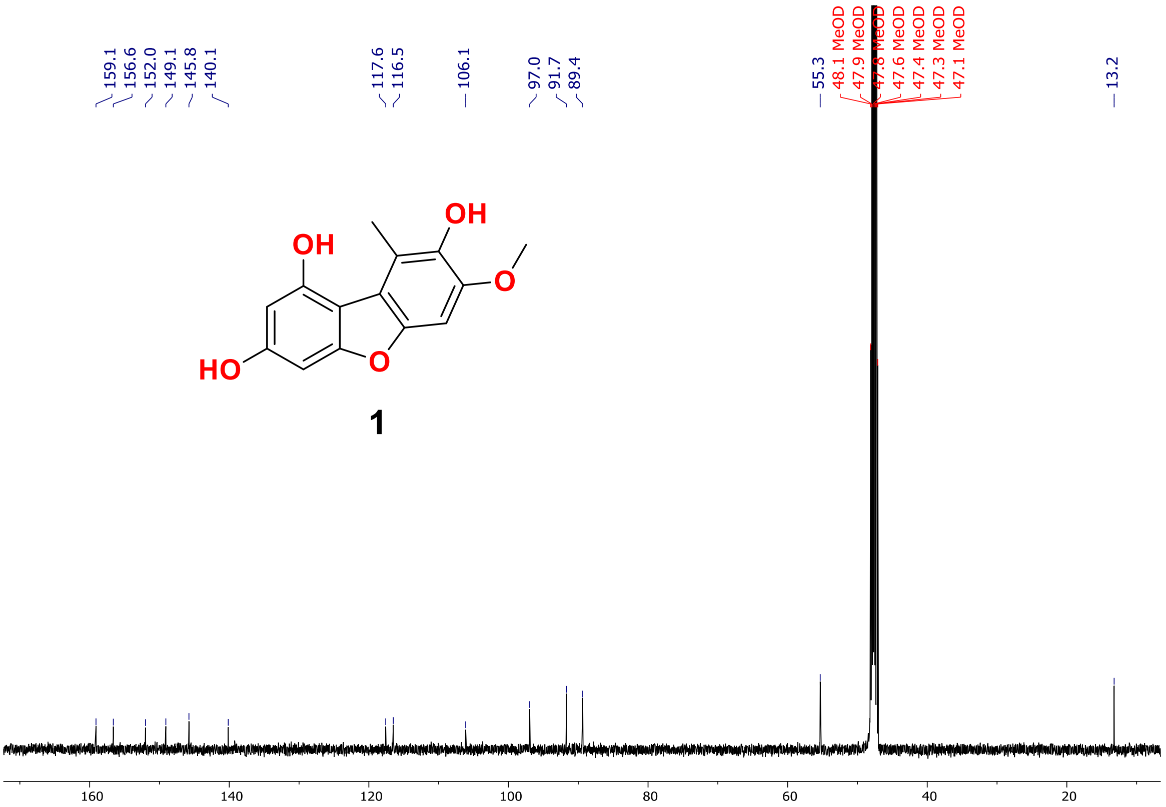

Figure S6 ^{13}C NMR spectrum of **1** (125 MHz, CD_3OD)

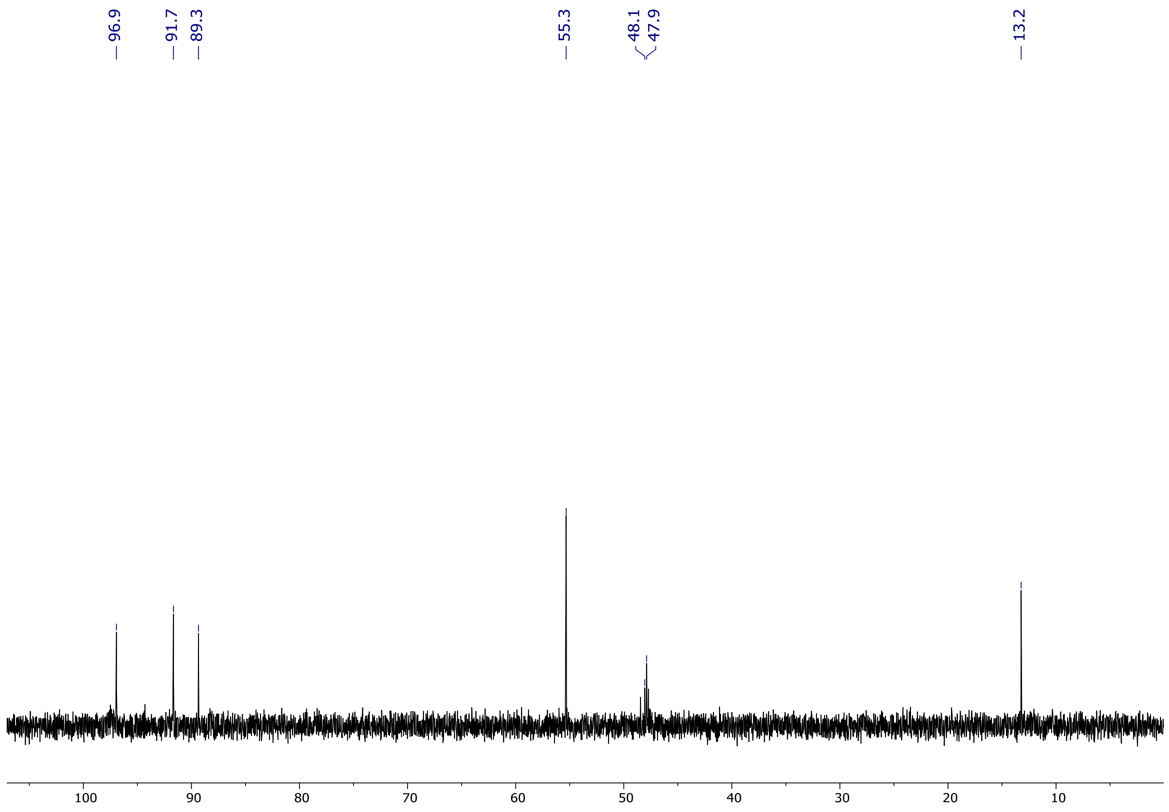

Figure S7 DEPT135 spectrum of **1**

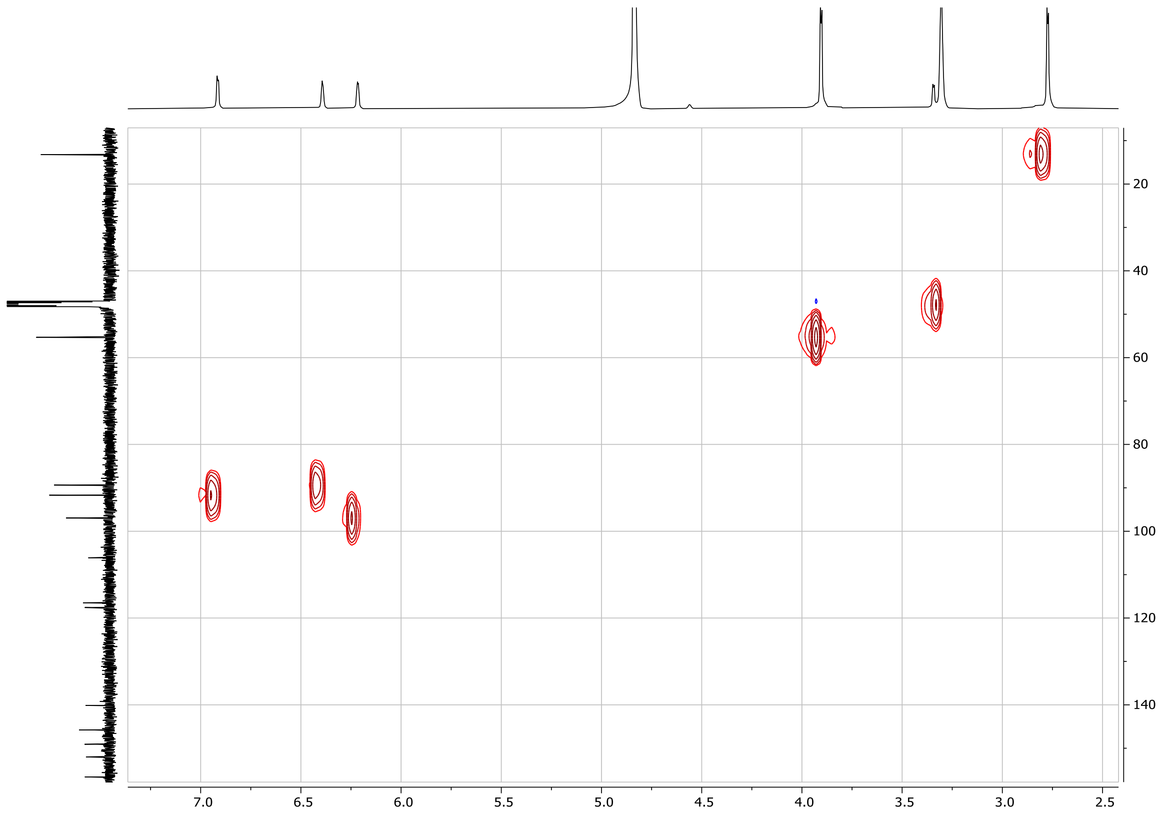

Figure S8 HSQC spectrum of **1**

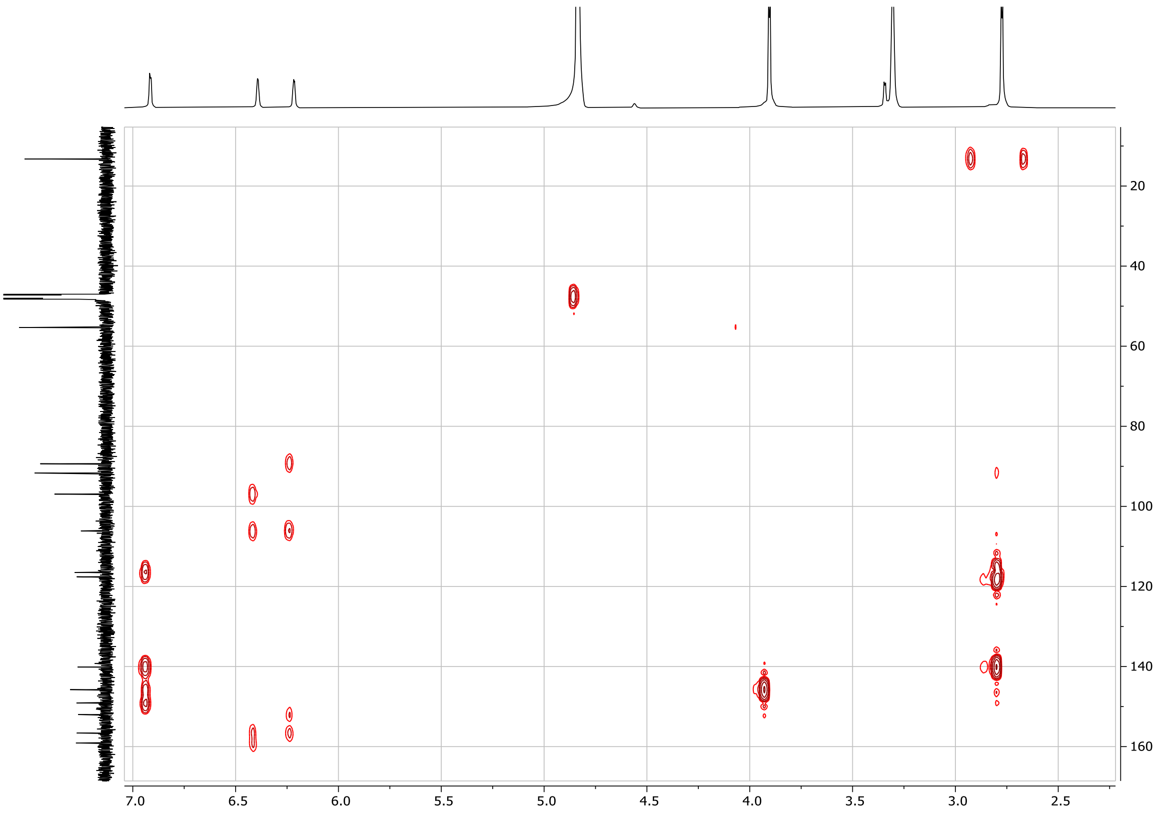

Figure S9 HMBC spectrum of **1**

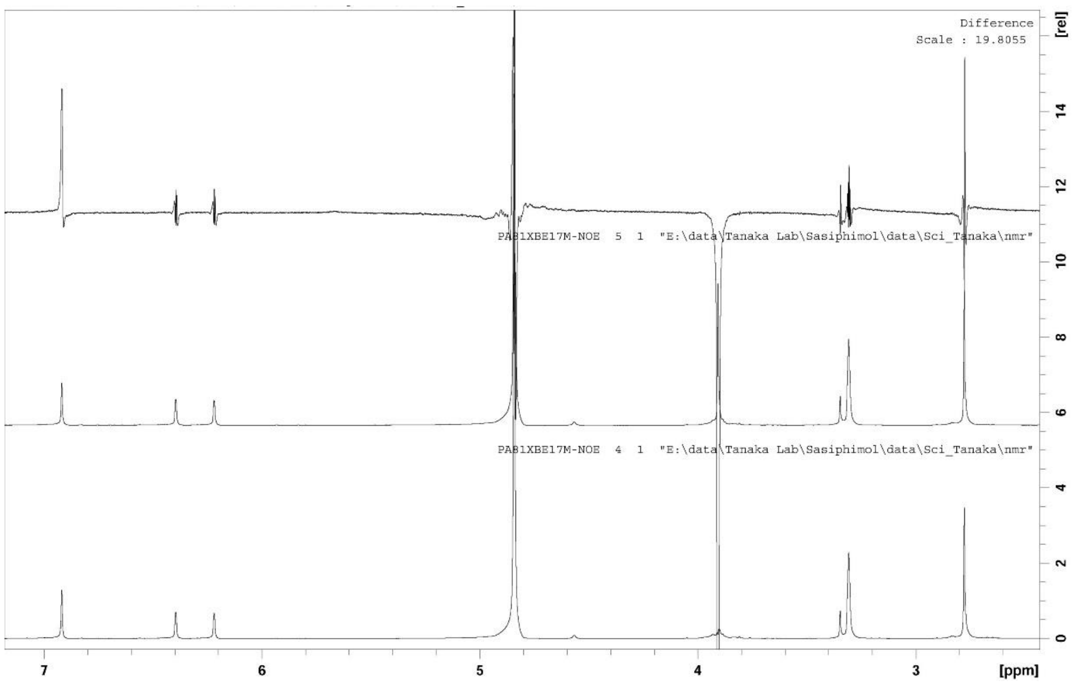

Figure S10 NOE (C-7-OCH_3) spectrum of **1**

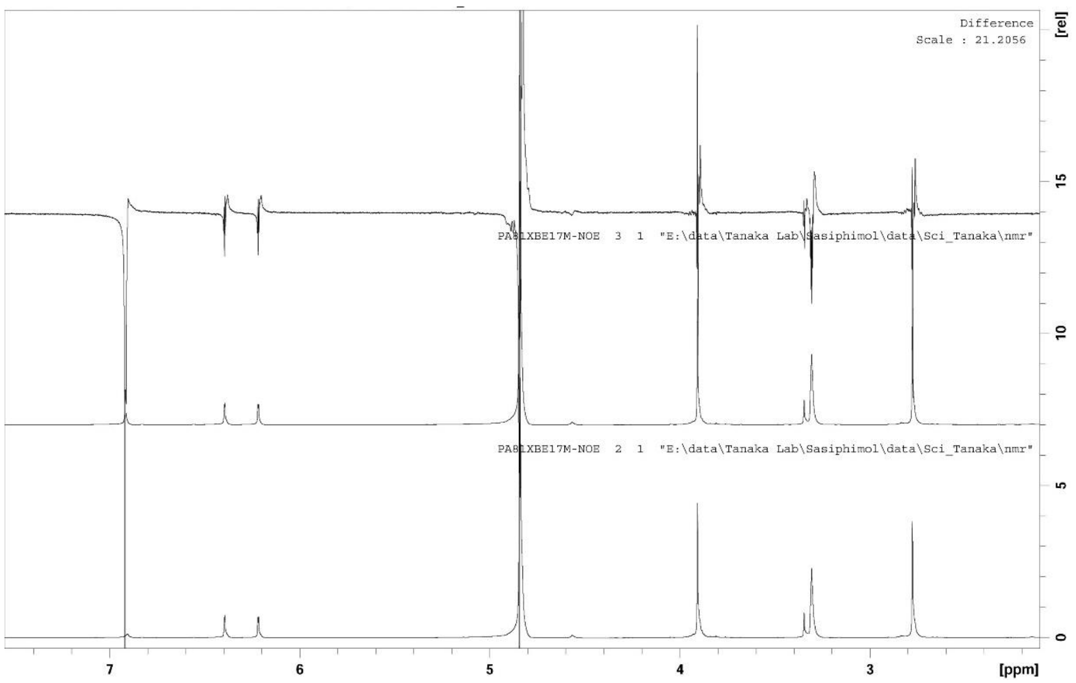

Figure S11 NOE (H-6) spectrum of **1**

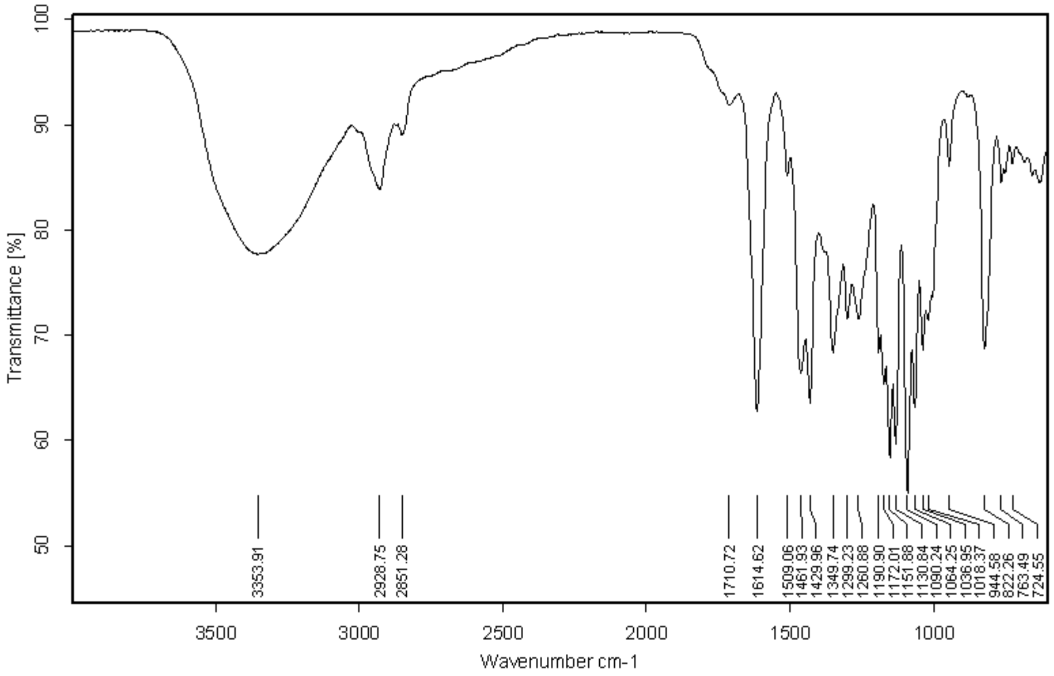

Figure S12 IR spectrum of **1**

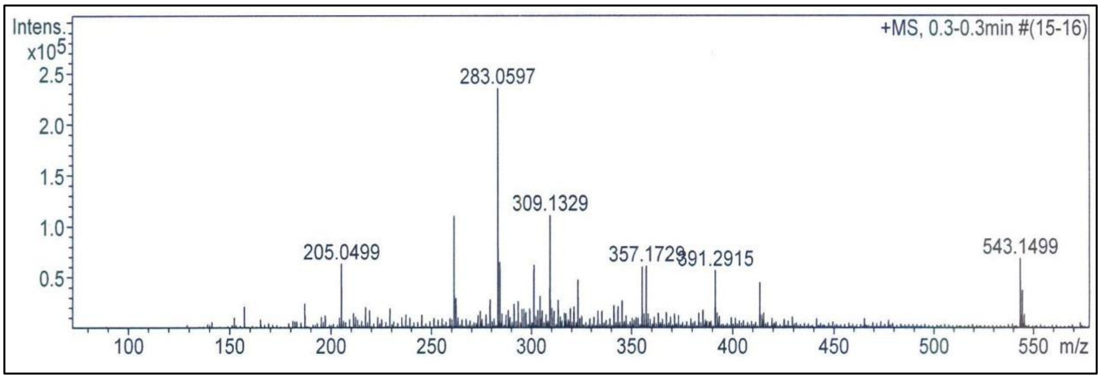

Figure S13 HRESIMS spectrum of **1**

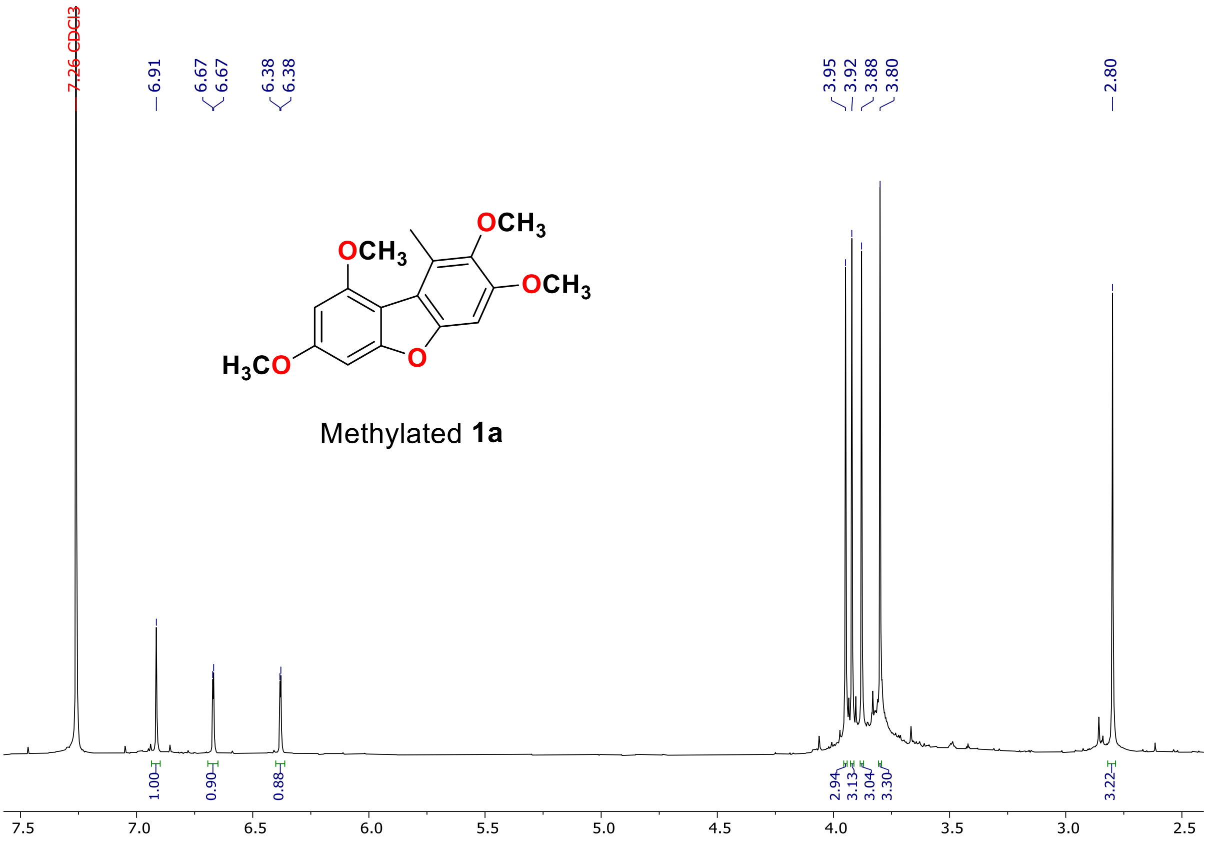

Figure S14 ^1H NMR spectrum of **1a** (500 MHz, $CDCl_3$)

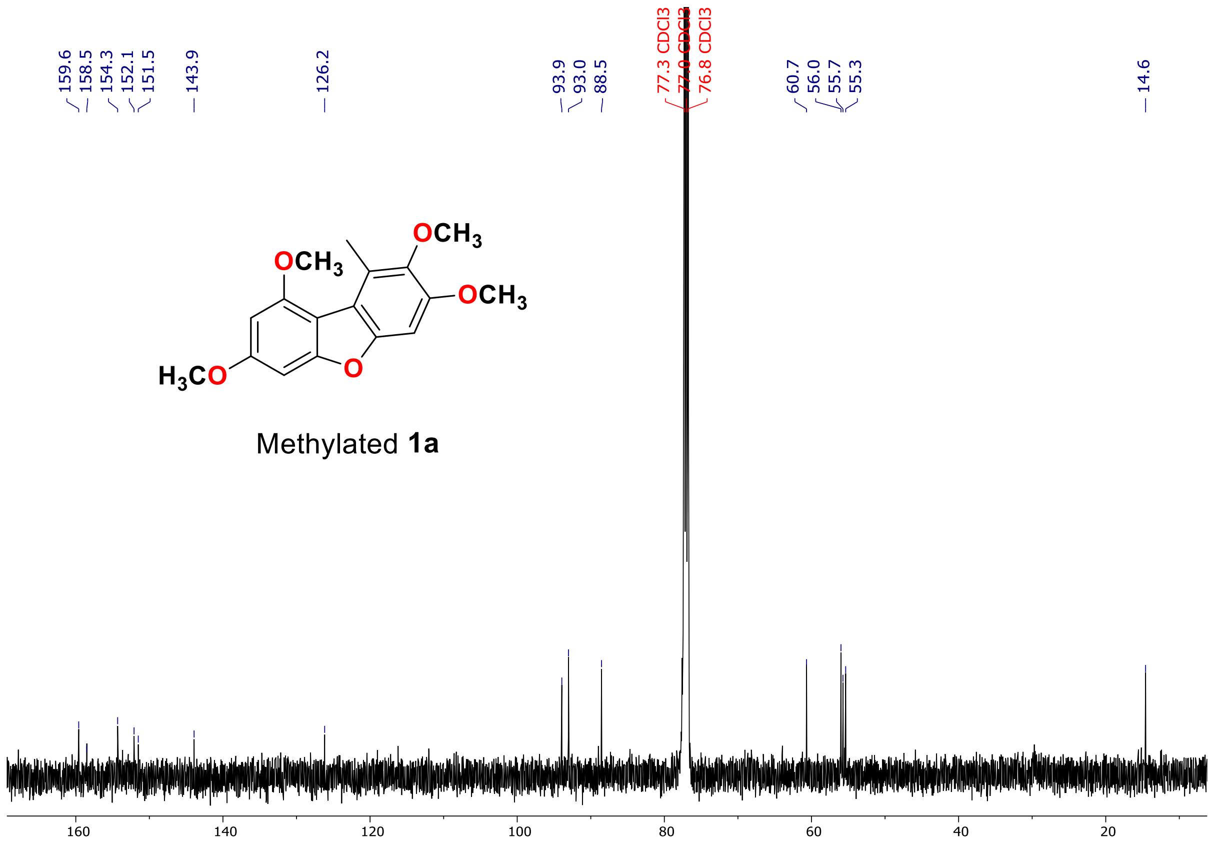

Figure S15 ^{13}C NMR spectrum of **1a** (125 MHz, $CDCl_3$)

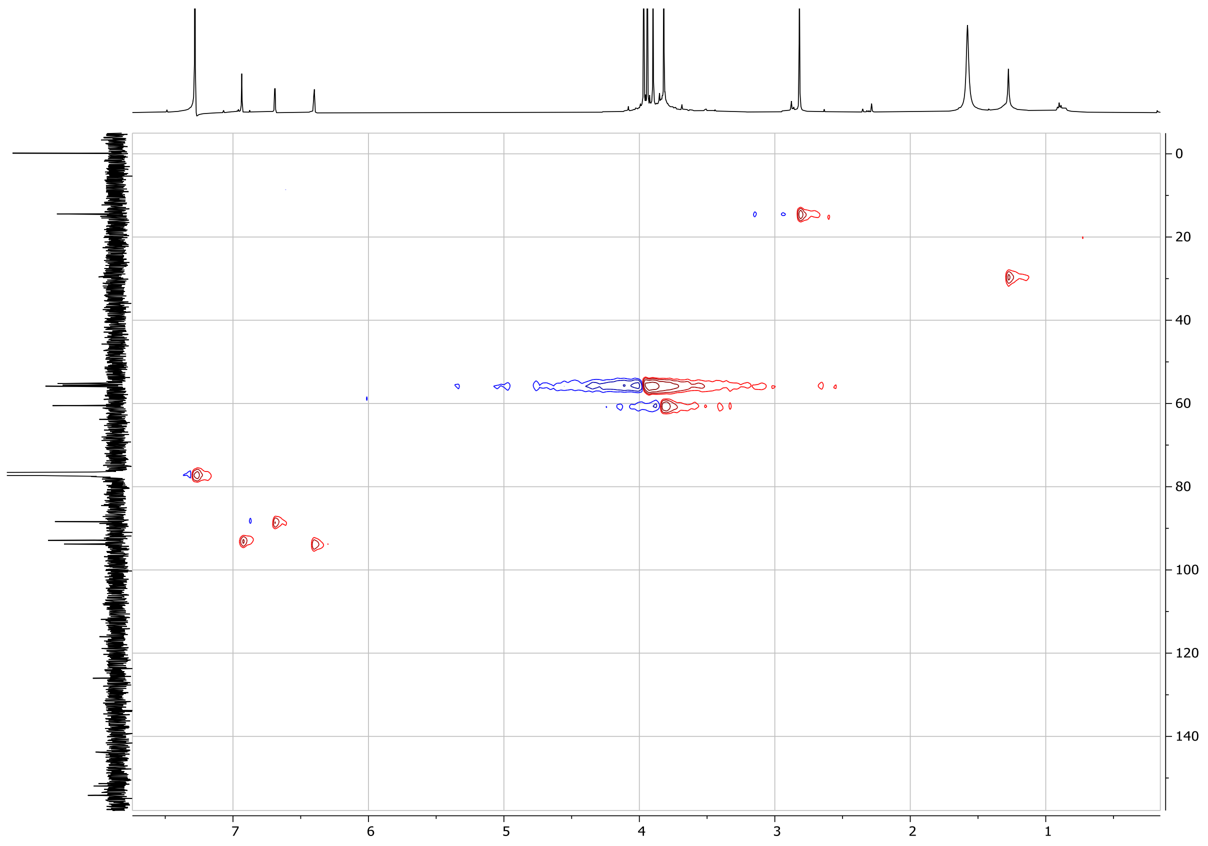

Figure S16 HSQC spectrum of **1a**

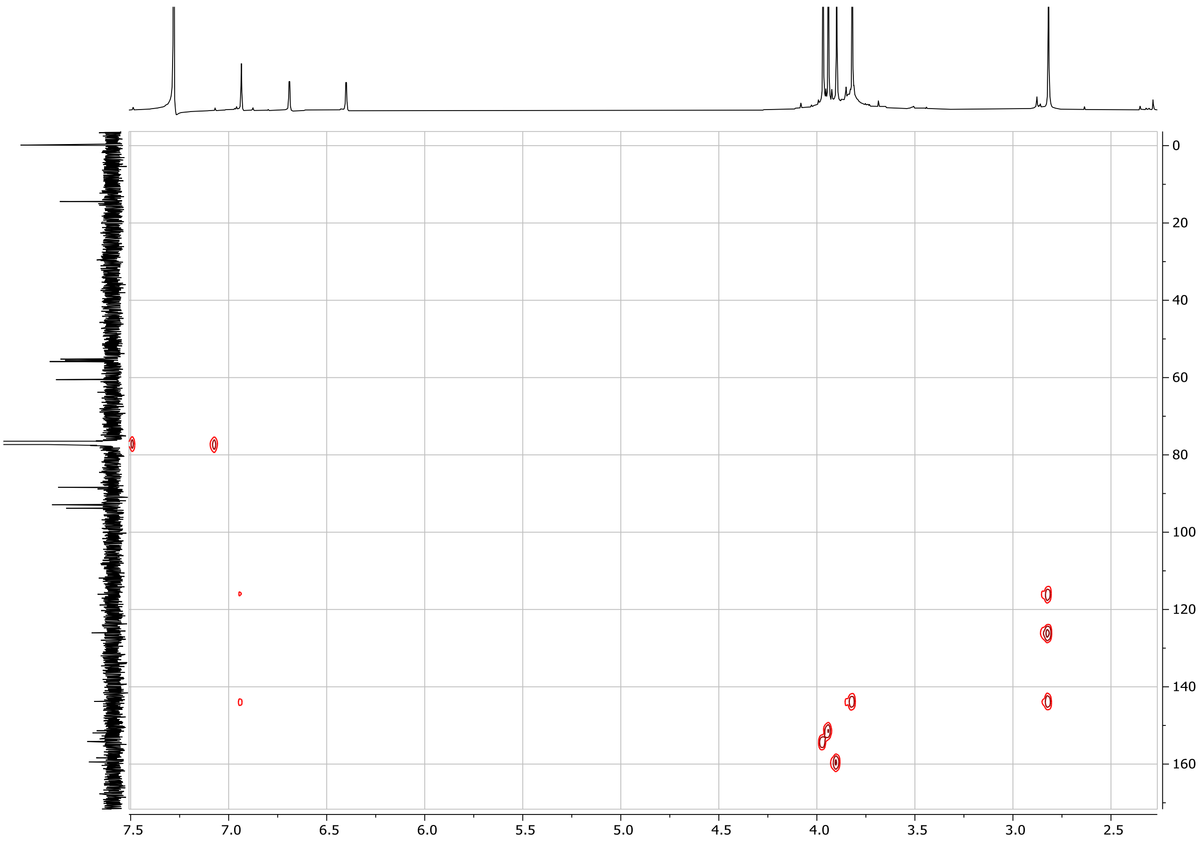

Figure S17 HMBC spectrum of **1a**

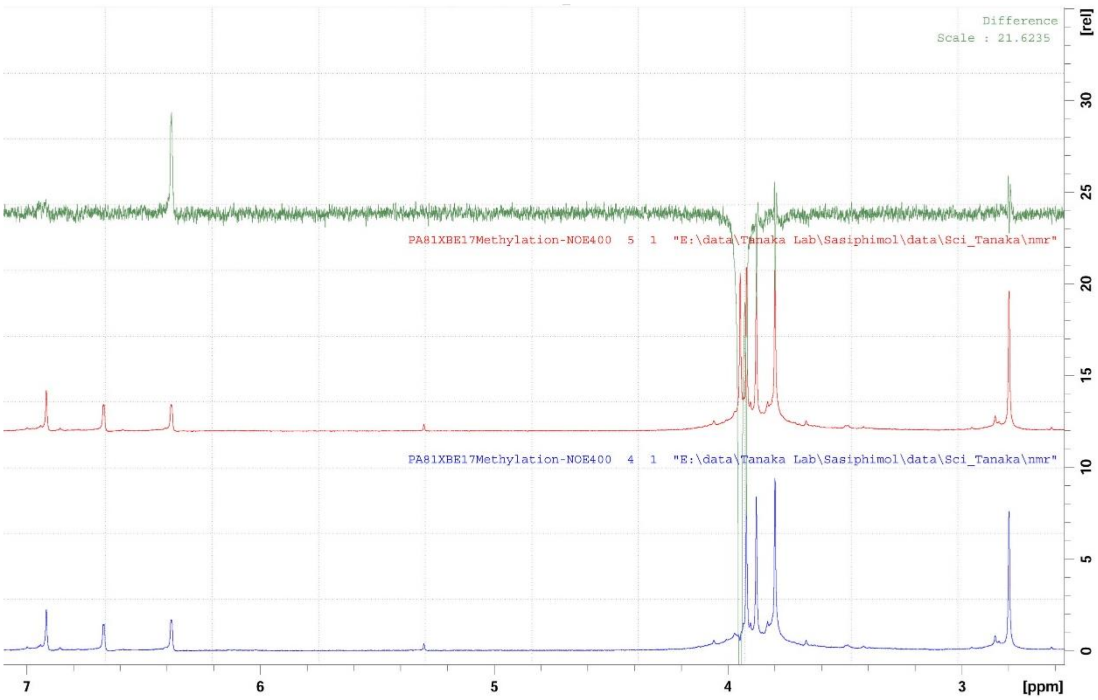

Figure S18 NOE (C-1-OCH_3) spectrum of **1a**

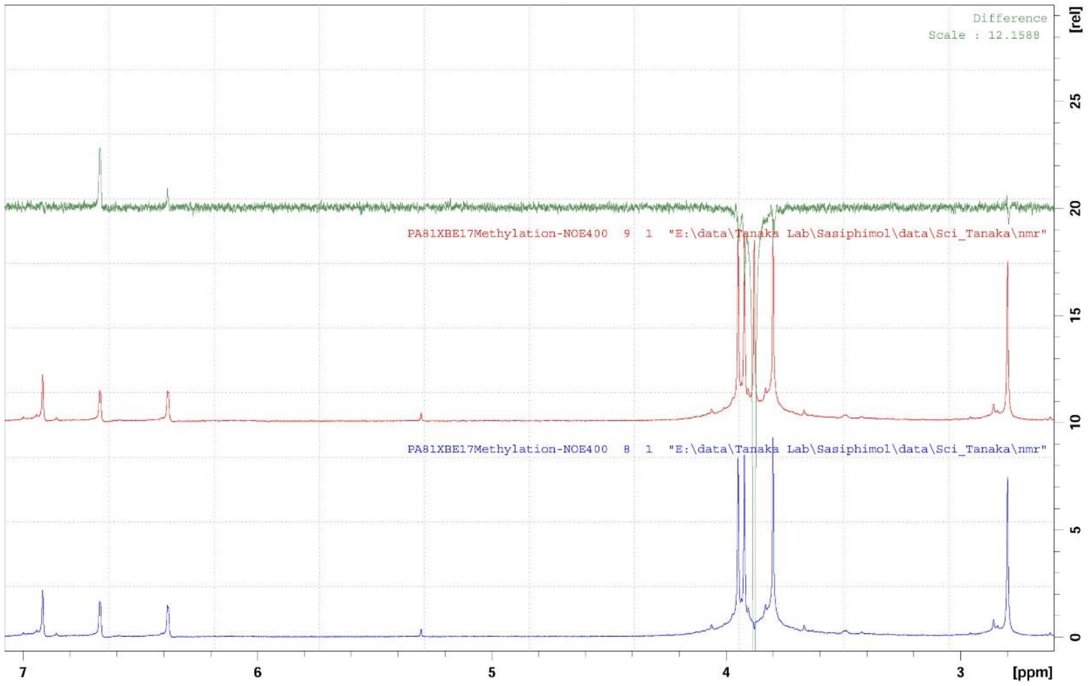

Figure S19 NOE (C-3-OCH$_3$) spectrum of **1a**

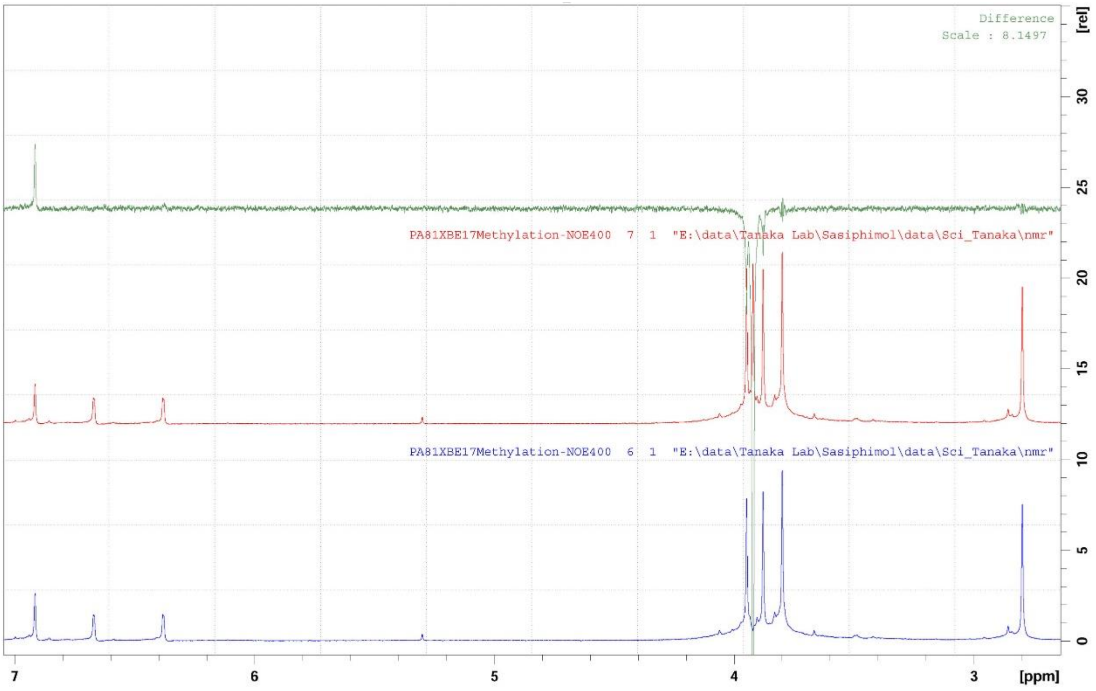

Figure S20 NOE (C-7-OCH$_3$) spectrum of **1a**

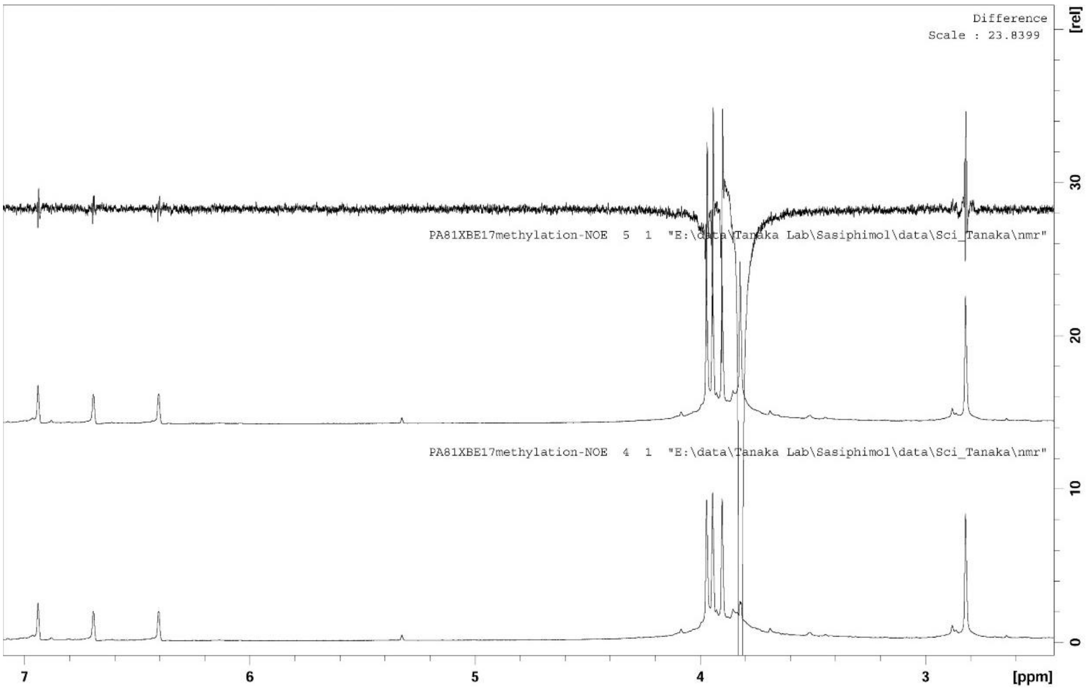

Figure S21 NOE (C-8-OCH_3) spectrum of **1a**

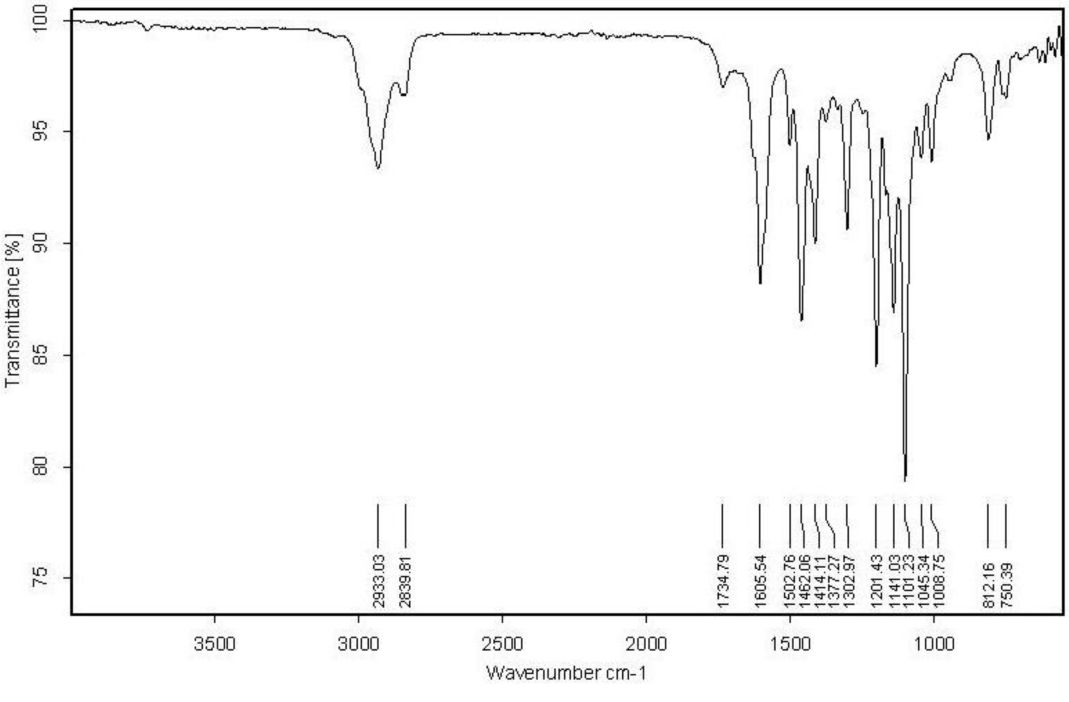

Figure S22 IR spectrum of compound **1a**

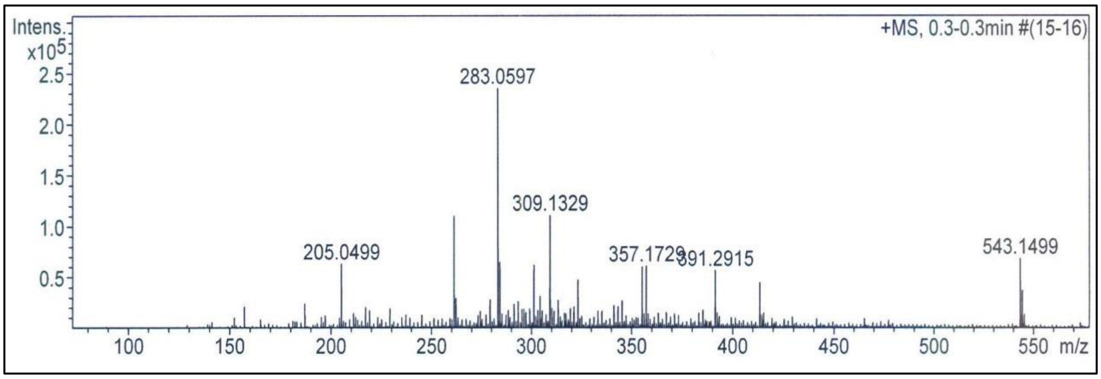

Figure S23 HRESIMS spectrum of **1a**

Table S3 ^{1}H, ^{13}C, 2D NMR and NOE data of compound **2** (400 MHz, $CDCl_3$)

Position /DEPT	δ_H	δ_C	COSY	HMBC	NOE
1 C		170.4			
2 O					
3 CH	4.65 (dqd, 12.5, 6.3, 3.2)	76.1	H-4, 9		
4 CH_2	3.82 (dd, 17.6, 3.2)	32.2	H-3	C-3, 4a, 5, 8a	
	3.00 (dd, 17.6, 11.7)			C-9	
4a C		134.4			
5 C		117.8			
6 CH	7.67 (s)	118.5		C-4a, 5, 7, 8, 10	
7 C		146.9			
8 C		156.4			
8a C		108.9			
3-CH_3	1.55 (d, 6.3)	20.7	H-3	C-3, 4	H-3, 4
5-CO		166.2			
5-$COOCH_3$	3.89 (s)	52.1		C-10	
7-OCH_3	3.94 (s)	56.3		C-7	H-6
OH	12.10 (s)			C-7, 8, 8a	

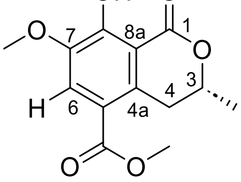

Figure S24 Structure of (3*R*)-7-methoxy-5-methoxycarbonylmellein (**2**)

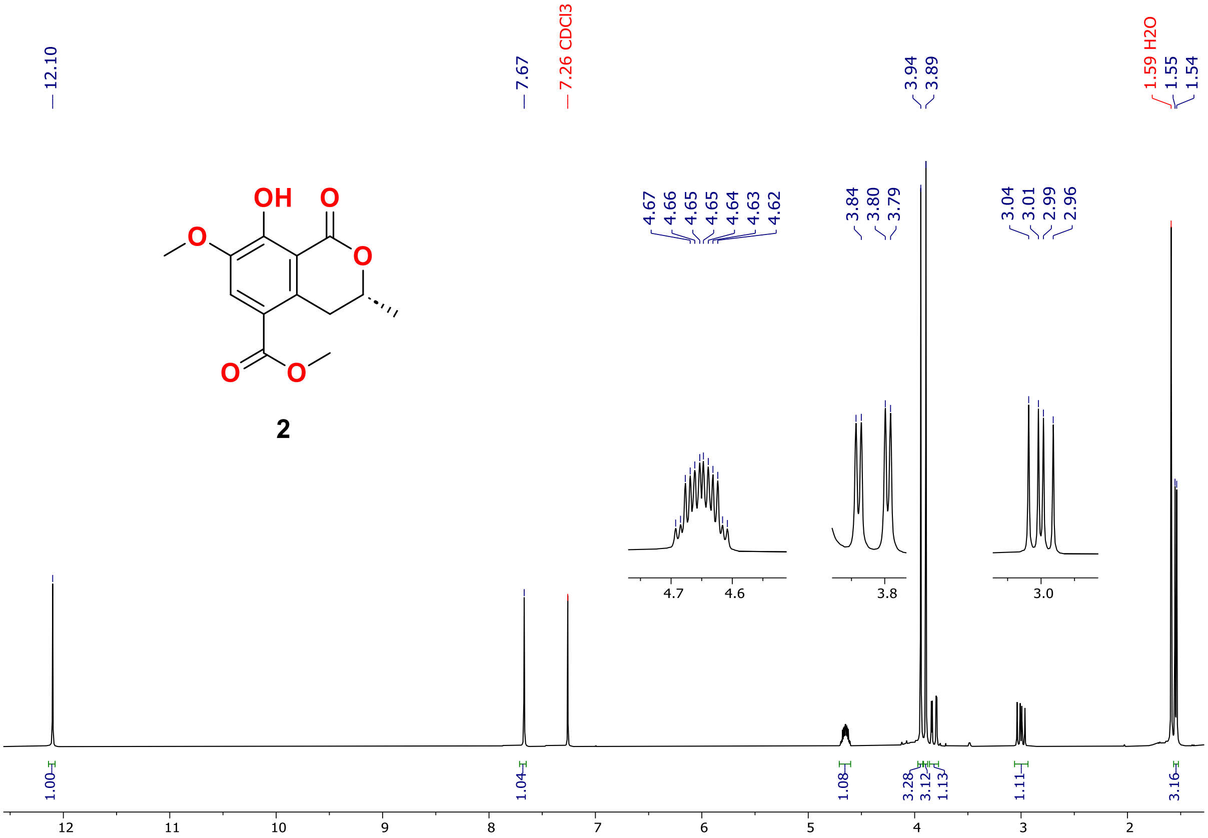

Figure S25 ^{1}H NMR spectrum of **2** (400 MHz, $CDCl_3$)

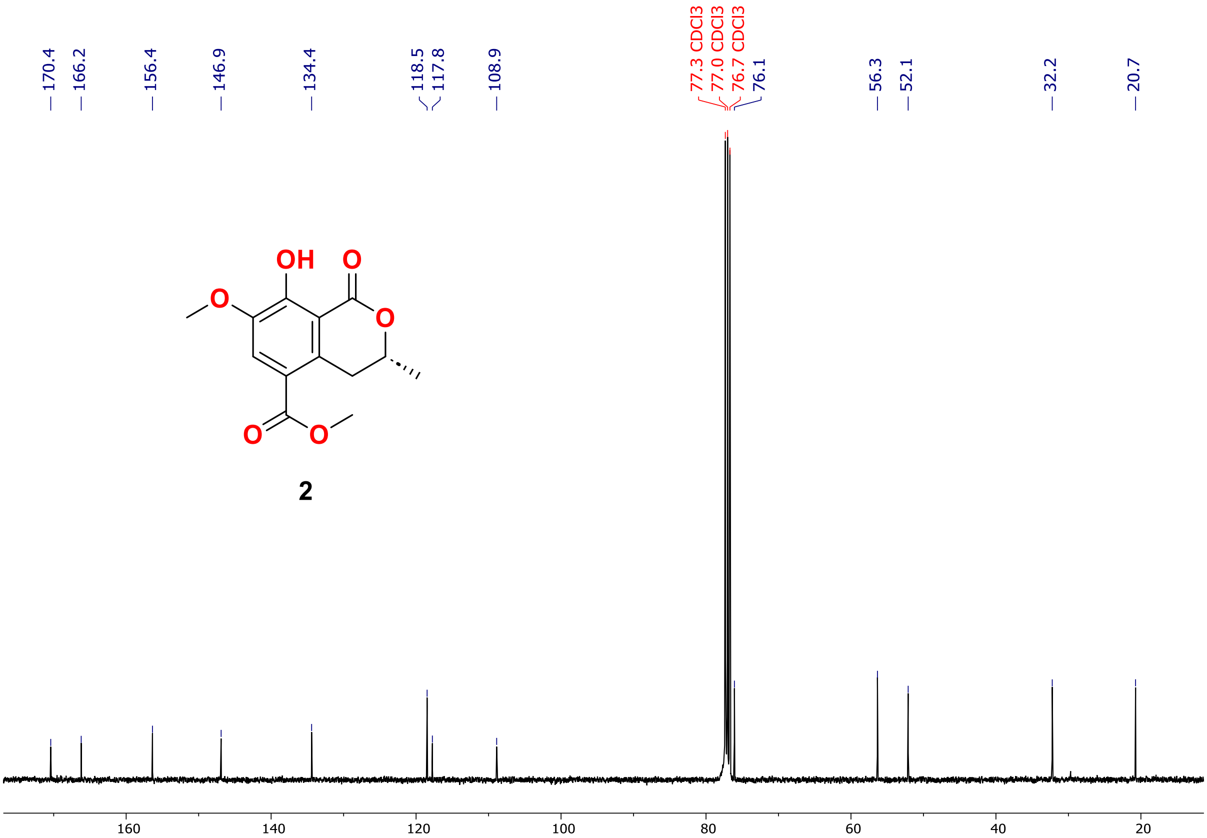

Figure S26 ^{13}C NMR spectrum of **2** (100 MHz, $CDCl_3$)

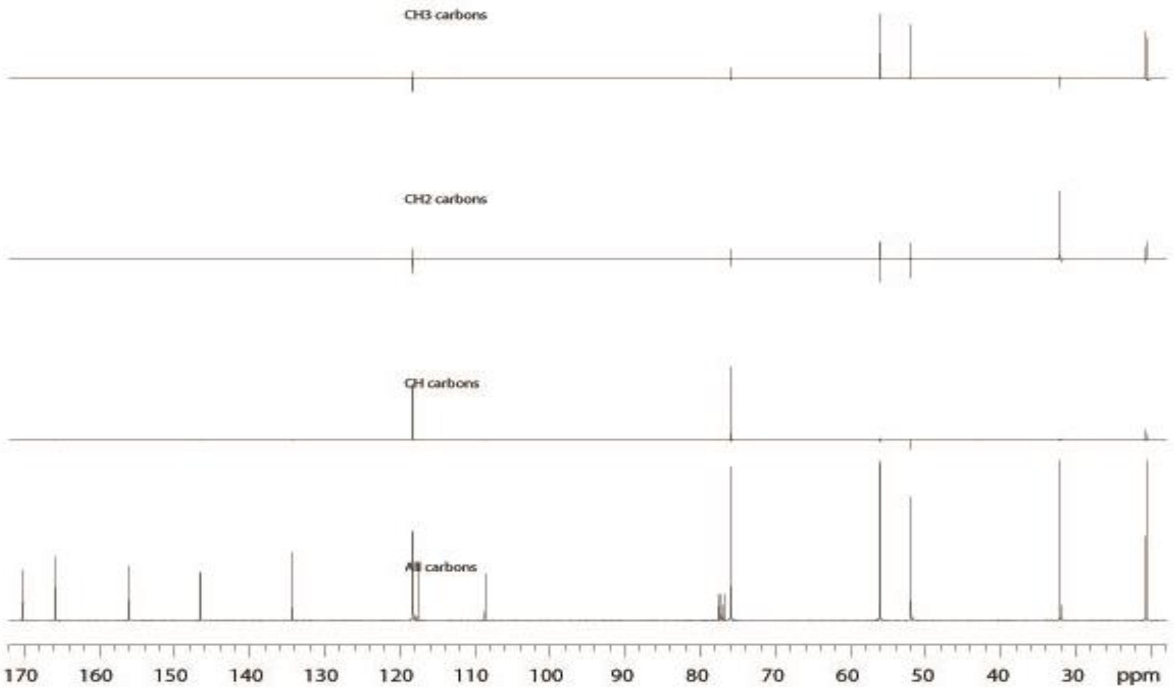

Figure S27 DEPT spectrum of **2**

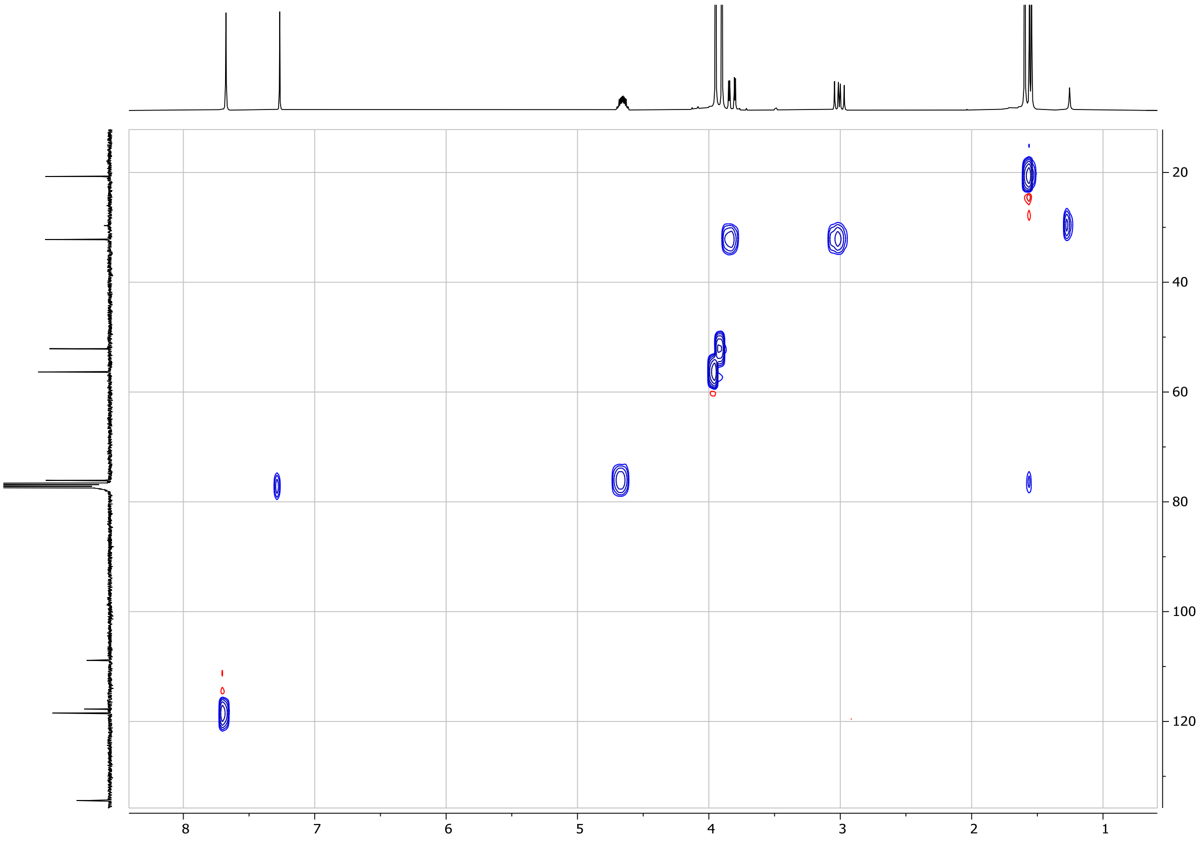

Figure S28 HMQC spectrum of **2**

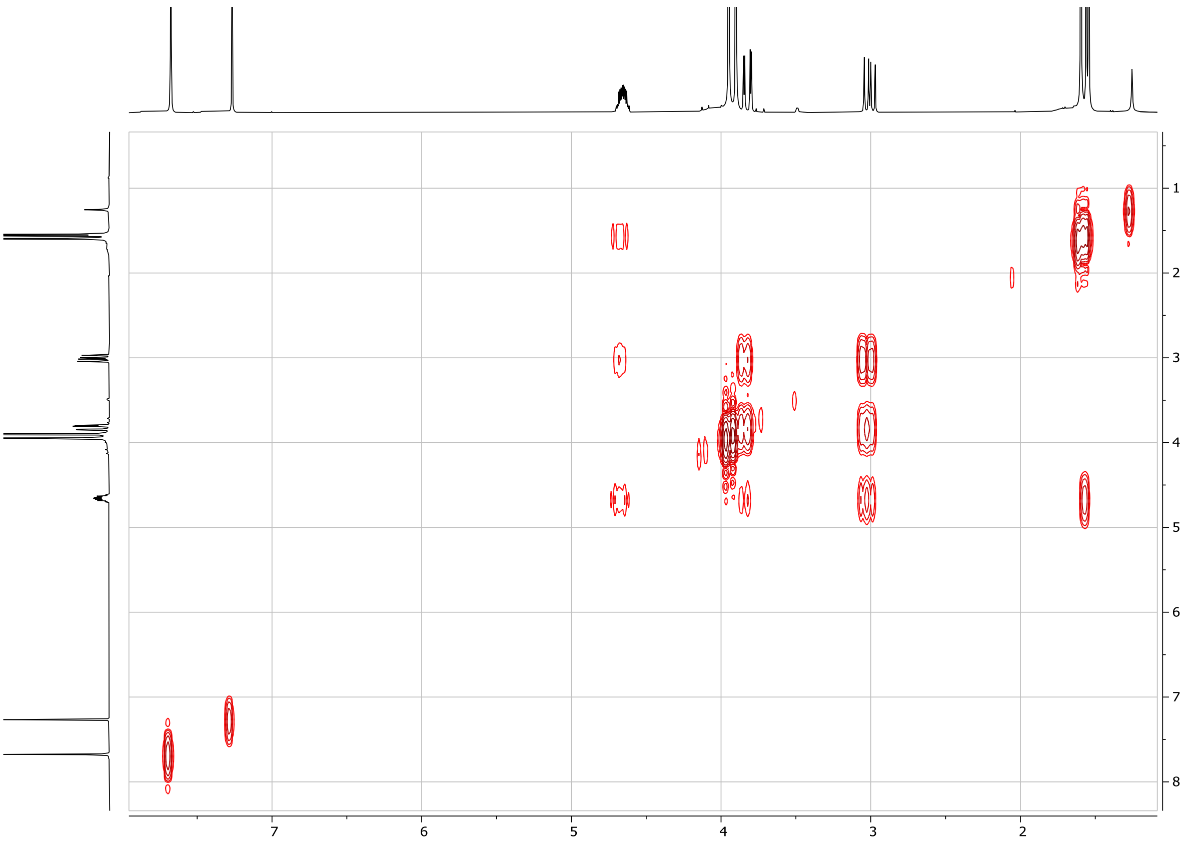

Figure S29 COSY spectrum of **2**

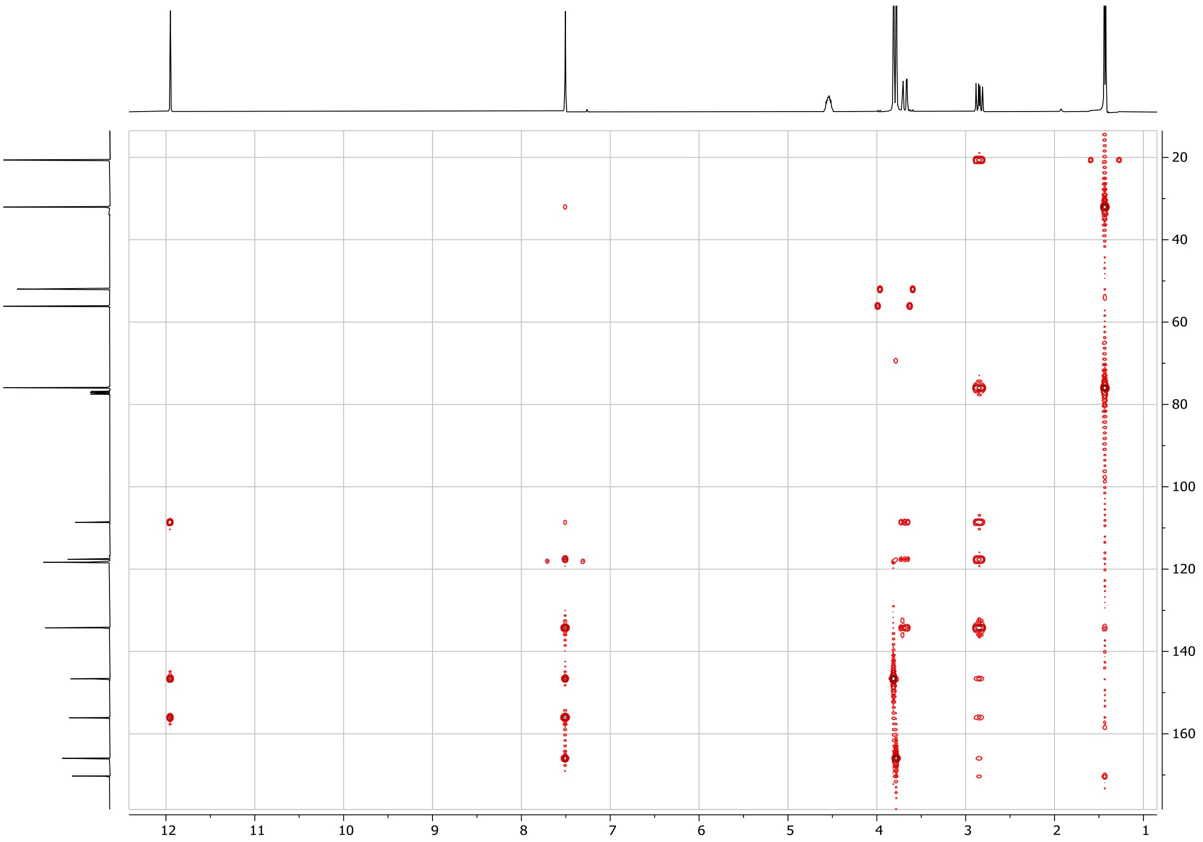

Figure S30 HMBC spectrum of **2**

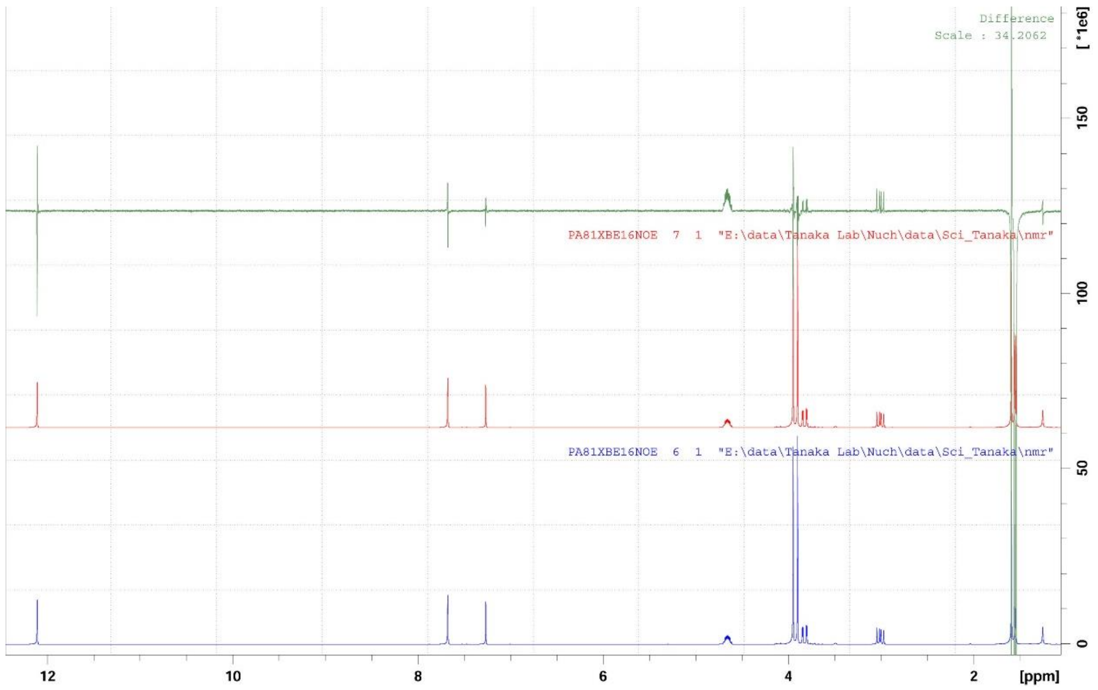

Figure S31 NOE (C-3-CH_3) spectrum of **2**

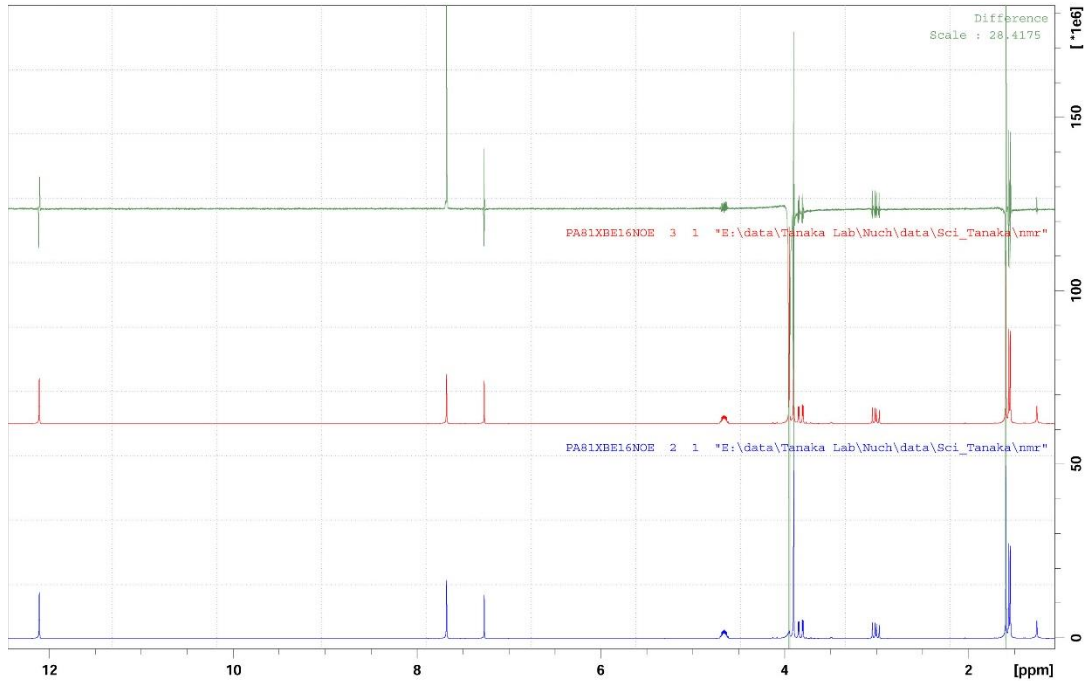

Figure S32 NOE (C-7-OCH_3) spectrum of **2**

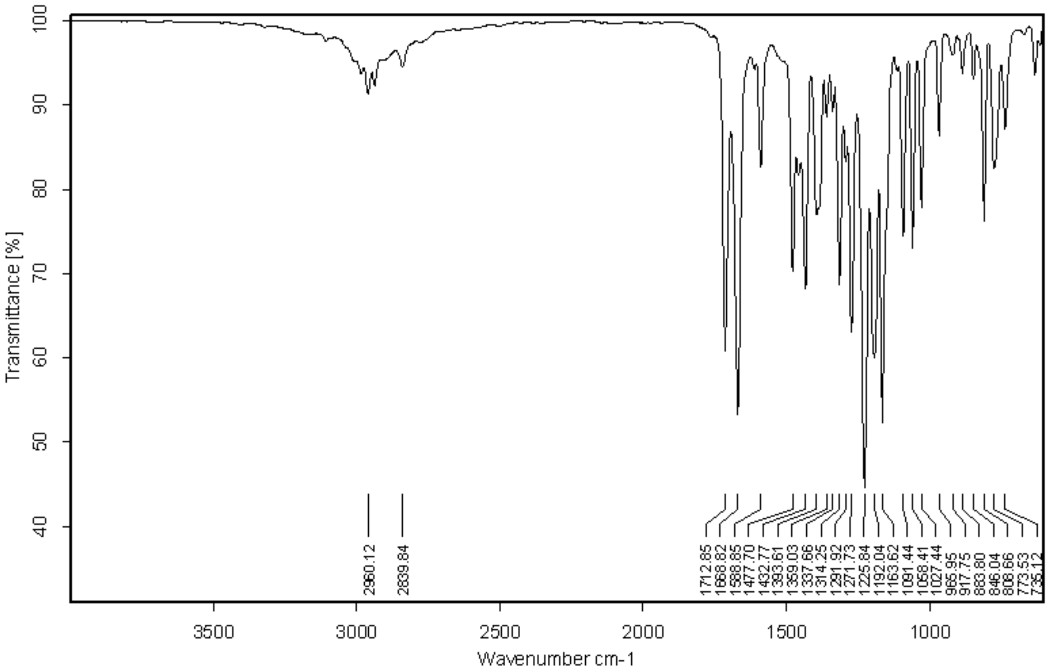

Figure S33 IR spectrum of **2**

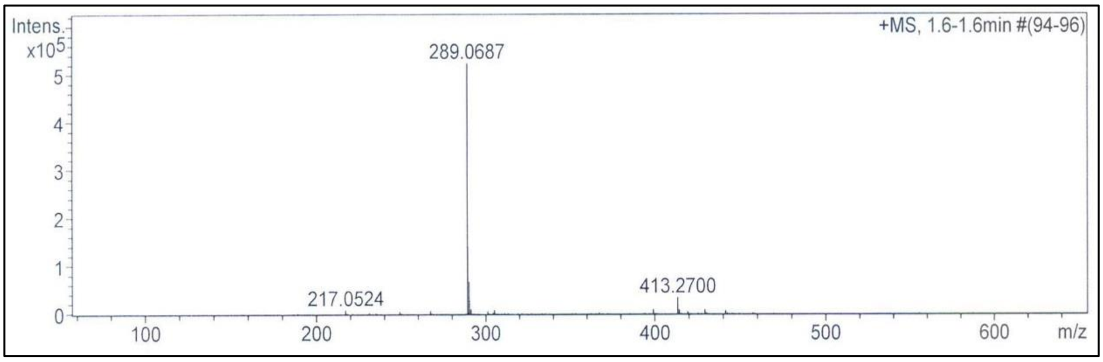

Figure S34 HRESIMS spectrum of **2**

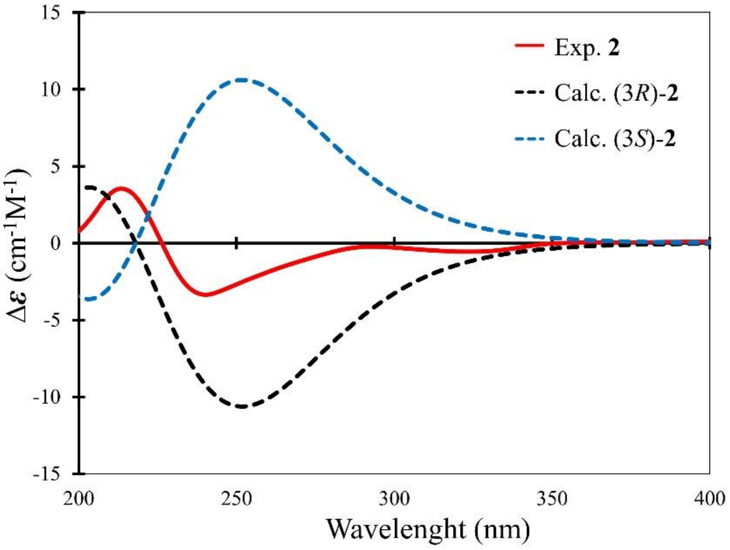

Figure S35 Experimental and calculated ECD spectra of **2**

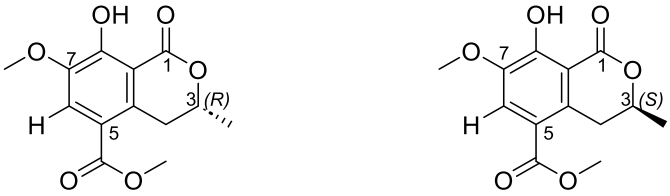

3*R*-isomer of **2** 3*S*-isomer of **2**

Figure S36 Structures of 3*R* and 3*S* isomers of **2**

Table S4 ^{1}H and ^{13}C NMR data of compound **3** (500 MHz, $CDCl_3$), (-)-mellein (600 MHz, $CDCl_3$) and (+)-mellein (400 MHz, $CDCl_3$)

Position /DEPT		**3**		**(–)-mellein** [1]		**(+)-mellein** [2]	
		δ_H	δ_C	δ_H	δ_C	δ_H [a]	δ_C
1	C		169.9		169.9		170.0
2	O						
3	CH	4.74 (sextet, 6.8)	76.1	4.74 (tq, 6.9, 6.3)	76.1	4.73 (sextet, 7.2)	76.2
4	CH_2	2.93 (d, 7.2)	34.6	2.93 (ddd, 6.9, 1.0, 0.7)	34.6	2.93 (d, 7.2)	34.7
4a	C		139.4		139.4		139.5
5	CH	6.69 (d, 7.4)	117.9	6.69 (dq, 7.4, 1.0)	117.9	6.89 (d, 8.4)	118.0
6	CH	7.41 (t, 8.0)	136.1	7.41 (dd, 8.4, 7.4)	136.1	7.40 (dd, 8.4, 7.6)	136.2
7	CH	6.89 (d, 8.4)	116.3	6.89 (ddd, 8.4, 1.0, 0.7)	116.2	6.69 (dd, 7.6, 1.2)	116.4
8	C		162.2		162.1		162.3
8a	C		108.3		108.3		108.4
3-CH_3		1.53 (d, 6.3)	20.7	1.53 (d, 6.3)	20.8	1.53 (d, 7.2)	20.9
OH		11.03 (brs)		11.03 (d, 0.5)		11.03 (s)	

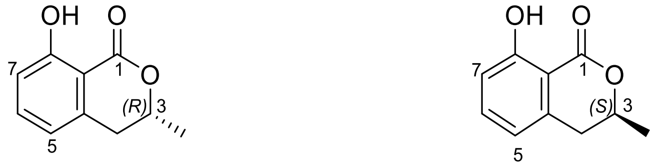

(-)-mellein (**3**)
$[\alpha]_D^{24.6}$ -144.8 (*c* 1.0, $CHCl_3$)
(lit. $[\alpha]_D^{22.0}$ -100.0 (*c* 1.0, $CHCl_3$))

(+)-mellein
$[\alpha]_D$ +92.0 (*c* 1.14, MeOH)

Figure S37. Structures of (-)-mellein (**3**) and (+)-mellein

Table S5 ^{1}H and ^{13}C NMR spectral data of compound **4** (500 MHz, $CDCl_3$) and (-)-5-methoxycarbonylmellein (300 MHz, $CDCl_3$)

Position	**4**		**(–)-5-methoxycarbonylmellein** [3]	
	δ_H	δ_C	δ_H	δ_C
1		170.0		170.1
2 O				
3	4.70 (dqd, 12.0, 6.2, 3.0)	75.6	4.68 (m)	75.6
4	3.91 (dd, 18.0, 3.0),<br>3.08 (dd, 17.8, 12.0)	32.6	3.88 (dd, 17.7, 3.0),<br>3.05 (dd, 17.7, 12.0)	32.6
4a		143.5		143.5
5		118.6		118.6
6	8.16 (d, 9.0)	138.5	8.13 (d, 9.0)	138.5
7	6.97 (d, 9.0)	116.2	6.94 (d, 9.0)	116.2
8		165.5		165.5
8a		108.9		108.9
3-CH_3	1.59 (d, 6.5)	20.8	1.57 (d, 6.3)	20.8
5-CO		166.2		166.2
5-$COOCH_3$	3.91 (s)	52.0	3.86 (s)	52.0
OH	11.86 (s)		11.83 (s)	

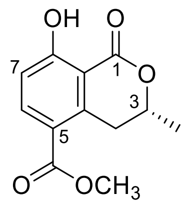

Figure S38. Structure of (-)-5-methoxycarbonylmellein (**4**)

Table S6 ^{1}H and ^{13}C NMR data of compound **5** (400 MHz, $CDCl_3$ and CD_3OD, 2/1 v/v) and (-)-5-carboxymellein (300 MHz, $CDCl_3$ and CD_3OD)

Position	**5**		**(–)-5-carboxymellein** [3]	
	δ_H	δ_C	δ_H	δ_C
1		170.2		170.2
2 O				
3	4.65 (m)	75.7	4.68 (m)	75.8
4	3.89 (dd, 18.0, 2.8)	32.6	3.92 (dd, 18.0, 3.5)	32.8
	3.00 (dd, 17.6, 11.6)		3.04 (dd, 18.0, 11.5)	
4a		143.4		143.7
5		119.4		119.2
6	8.14 (d, 8.8)	139.0	8.18 (d, 9.0)	139.2
7	6.89 (d, 8.8)	115.8	6.94 (d, 9.0)	116.2
8		165.0		165.4
8a		108.7		109.0
3-CH_3	1.51 (d, 6.0)	20.4	1.56 (d, 7.0)	20.8
5-COOH		167.9		168.0

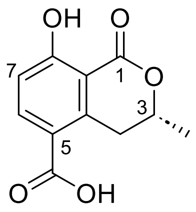

Figure S39. Structure of (-)-5-carboxymellein (**5**)

Table S7 ^{1}H and ^{13}C NMR data of compound **6** (400 MHz, $CDCl_3$) and cytochalasin D (300 MHz, $CDCl_3$)

**Position/ DEPT**	**6**		**Cytochalasin D** [3]	
	δ_H	δ_C	δ_H	δ_C
1 C		173.7		173.7
2 NH	5.62 (s)		5.59 (brs)	
3 CH	3.22 (m)	53.3	3.24 (d, 4.5)	53.3
4 CH	2.82 (m)	47.0		47.0
5 CH	2.14 (t, 4.4)	49.9	2.15 (dd, 5.1, 3.6)	50.0
6 C		147.6		147.6
7 CH	3.80 (d, 10.8)	69.8	3.81 (d, 10.2)	69.8
8 CH	2.82 (m)	32.6	2.76 (m)	32.7
9 C		53.5		53.5
10 CH_2	2.71 (m)	45.3	2.76 (m)	45.3
11 CH_3	0.93 (d, 6.4)	13.6	0.94 (d, 6.6)	13.7
12 CH_2	5.28 (brs), 5.07 (brs)	114.4	5.30 (brs), 5.09 (brs)	114.4
13 CH	5.33 (ddd, 15.6, 10.6, 5.0)	134.1	5.34 (ddd, 15.6, 10.5, 5.1)	134.7
14 CH	5.68 (dd, 15.8, 9.6)	130.6	5.69 (dd, 15.6, 9.9)	130.6
15 CH_2	2.50 (q, 12.8), 2.01 (dd, 13.0, 5.0)	37.7	2.51 (dd, 12.6, 11.1), 2.03 (m)	37.7
16 CH	2.71 (m)	42.3	2.76 (m)	42.3
17 C		210.2		210.2
18 C		77.1		77.1
19 CH	5.13 (dd, 15.6, 1.6)	127.6	5.14 (dd, 15.6, 2.4)	127.6
20 CH	6.10 (dd, 15.8, 2.8)	132.3	6.12 (dd, 15.6, 2.7)	132.3
21 CH	5.62 (d, 1.6)	77.7	5.63 (t, 2.4)	77.7
23 C		169.7		169.7
24 CH_3	2.25 (s)	20.8	2.27 (s)	20.8
25 CH_3	1.19 (d, 6.4)	19.4	1.20 (d, 6.9)	19.4
26 CH_3	1.50 (s)	24.2	1.51 (s)	24.2
1′ C		137.2		137.3
2′, 6′ CH	7.13 (d, 6.8)	129.1	7.14 (dd, 8.1, 1.5)	129.1
3′, 5′ CH	7.31 (t, 6.8)	128.9	7.28 (m)	128.9
4′ CH	7.22 (t, 8.0)	127.1	7.32 (m)	127.1

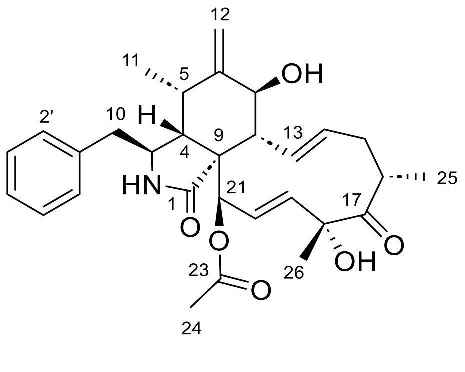

Figure S40. Structure of cytochalasin D (**6**)

Table S8 ^{1}H and ^{13}C NMR data of compound **7** (400 MHz, $CDCl_3$) and zygosporin D (400 MHz, $CDCl_3$)

Position/ DEPT	7		Zygosporin D [4]	
	δ_H	δ_C	δ_H	δ_C
1 C		175.1		175.0
2 NH	5.85 (s)		5.53 (s)	
3 CH	3.27 (dd, 8.8, 4.0)	53.5	3.29 (ddd, 8.3, 4.3, 3.8)	53.5
4 CH	2.55 (m)	50.0	2.57 (m)	50.0
5 CH	2.86 (m)	32.9	2.90-2.80 (m)	32.9
6 C		148.2		148.1
7 CH	3.77 (d, 11.2)	69.7	3.79 (d, 10.7)	69.7
8 CH	2.88 (m)	45.6	2.88 (dd, 10.7, 10.2)	45.6
9 C		54.3		54.2
10 CH_2	2.88 (m), 2.56 (m)	45.3	2.88 (dd, 13.6, 3.5), 2.55 (m)	45.3
11 CH_3	1.08 (d, 6.4)	13.8	1.11 (d, 6.8)	13.9
12 CH_2	5.30 (d, 1.6), 5.10 (s)	114.0	5.31 (s), 5.11 (s)	114.0
13 CH	5.64 (dd, 15.8, 10.2)	127.0	5.65 (dd, 15.9, 10.2)	127.0
14 CH	5.27 (ddd, 15.6, 10.4, 5.2)	130.9	5.27 (ddd, 15.9, 10.8, 5.4)	130.9
15 CH_2	2.47 (d, 12.4), 1.99 (dd, 12.6, 5.0)	37.7	2.48 (ddd, 13.0, 11.0, 10.8), 2.00 (ddd, 13.0, 5.4, 1.6)	37.7
16 CH	2.72 (dd, 10.8, 6.8)	42.3	2.72 (ddq, 11.0, 1.6, 6.5)	42.3
17 C		210.3		210.2
18 C		77.7		77.7
19 CH	5.41 (dd, 15.8, 2.0)	133.8	5.41 (dd, 16.2, 2.6)	133.7
20 CH	6.19 (dd, 16.0, 2.4)	137.2	6.21 (dd, 16.2, 2.4)	137.1
21 CH	4.03 (s)	76.4	4.05 (dd, 2.6, 2.4)	76.4
22 CH_3	1.18 (d, 7.2)	19.4	1.19 (d, 6.8)	19.4
23 CH_3	1.54 (s)	24.2	1.55 (s)	24.2
1′ C		137.3		137.3
2′, 6′ CH	7.12 (d, 7.2)	129.2	7.12 (m)	129.2
3′, 5′ CH	7.31 (t, 7.2)	128.9	7.32 (m)	128.9
4′ CH	7.24 (t, 7.2)	127.1	7.26 (m)	127.1

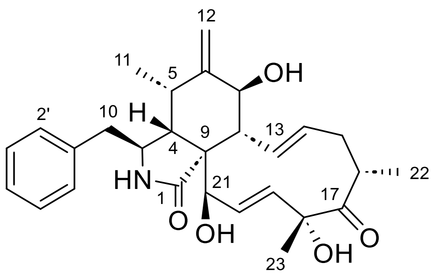

Figure S41 Structure of zygosporin D (**7**)

Table S9 ^{1}H and ^{13}C NMR data of compound **8** (400 MHz, $CDCl_3$) and 19,20-epoxycytochalasin D (400 MHz, $CDCl_3$)

Position/	**8**		**19,20-Epoxycytochalasin D** [5]	
**DEPT**	δ_H	δ_C	δ_H	δ_C
1 C		173.6		173.4
2 NH	5.76 (s)		5.46 (brs)	
3 CH	3.24 (m)	53.9	3.24 (m)	53.9
4 CH	2.24 (t, 3.8)	50.5	2.25 (dd, 5.2, 3.3)	50.7
5 CH	2.62 (m)	32.5	2.62 (m)	32.6
6 C		147.5		147.4
7 CH	3.81 (d, 10.4)	69.9	3.81 (brd, 10.1)	70.0
8 CH	2.62 (m)	46.5	2.62 (m)	46.5
9 C		52.5		52.4
10 CH_2	2.85 (dd, 13.6, 5.6),<br>2.74 (dd, 13.2, 8.8)	45.0	2.85 (dd, 13.4, 5.0),<br>2.73 (dd, 13.4, 9.1)	45.2
11 CH_3	0.86 (d, 6.4)	13.5	0.89 (d, 6.9)	13.5
12 CH_2	5.26 (s),<br>5.05 (s)	114.3	5.27 (brs),<br>5.06 (brs)	114.4
13 CH	5.88 (dd, 15.6, 9.6)	131.2	5.89 (dd, 15.5, 9.8)	131.2
14 CH	5.69 (ddd, 15.4, 9.6, 5.6)	133.3	5.69 (ddd, 15.5, 9.9, 5.8)	133.5
15 CH_2	2.62 (m),<br>2.10 (dd, 12.8, 5.6)	37.4	2.62 (m),<br>2.09 (m)	37.4
16 CH	3.20 (m)	41.9	3.22 (m)	41.9
17 C		215.3		215.3
18 C		76.4		76.3
19 CH	3.15 (s)	59.6	3.14 (d, 1.9)	59.6
20 CH	3.52 (s)	52.7	3.53 (dd, 1.9, 0.8)	52.7
21 CH	5.50 (s)	74.1	5.51 (brs)	74.1
23 C		169.8		169.8
24 CH_3	2.14 (s)	20.6	2.14 (s)	20.7
25 CH_3	1.19 (d, 6.8)	19.1	1.18 (d, 6.6)	19.2
26 CH_3	1.52 (s)	21.8	1.53 (s)	21.9
1′ C		137.1		137.1
2′, 6′ CH	7.16 (d, 7.6)	129.2	7.32 (m)	129.1
3′, 5′ CH	7.31 (d, 7.6)	128.9	7.15 (m)	129.0
4′ CH	7.23 (t, 7.6)	127.1	7.24 (m)	127.1

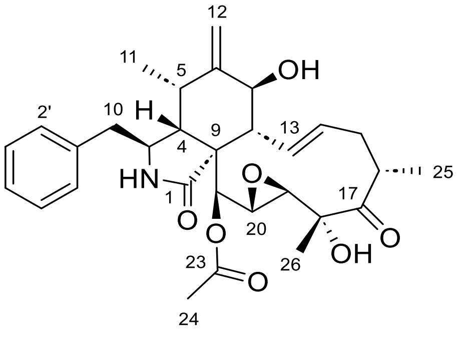

Figure S42 Structure of 19,20-epoxycytochalasin D (**8**)

Table S10 ^{1}H and ^{13}C NMR data of compound **9** (400 MHz, CDCl3) and cytochalasin Ohyp (^{1}H NMR 500 MHz, $CDCl_3$ and ^{13}C NMR 400 MHz, pyridine-d_5)

Position/ DEPT	**9**		**Cytochalasin O$_{hyp}$**	
	δ_H ($CDCl_3$)	δ_C ($CDCl_3$)	δ_H ($CDCl_3$) [6]	δ_C (Pyridine-d_5) [7]
1 C		174.4		175.7
2 NH	5.29 (s)		5.74 (s)	
3 CH	3.90 (m)	54.2	3.54 (m)	54.2
4 CH	1.93 (t, 5.0)	50.9	1.97-2.19 (m)	50.0
5 CH	1.74 (m)	38.1	1.97-2.19 (m)	39.8
6 C		76.2		72.6
7 CH	3.28 (d, 11.2)	74.8	2.88-3.07 (m)	73.4
8 CH	2.45 (m)	45.9	2.88-3.07 (m)	44.7
9 C		54.9		55.5
10 CH_2	2.70 (m), 2.55 (m)	45.5	2.44-2.59 (m), 2.88-3.07 (m)	46.6
11 CH_3	1.01 (d, 7.6)	12.4	1.05 (d, 7.5)	13.5
12 CH_3	1.15 (s)	22.6	1.22 (s)	24.6
13 CH	5.49 (dd, 15.6, 9.6)	128.9	5.59 (dd, 15.0, 9.0)	131.0
14 CH	5.18 (m)	134.9	5.32 (ddd, 15.0, 11.0, 5.0)	134.6
15 CH_2	2.48 (m), 2.02 (dd, 12.8, 4.4)	37.9	2.44-2.59 (m), 1.97-2.19 (m)	38.8
16 CH	2.92 (dd, 13.6, 4.0)	42.4	2.74 (m)	42.6
17 C		210.8		211.4
18 C		77.6		78.8
19 CH	5.09 (dd, 15.8, 2.2)	126.8	5.15 (dd, 15.0, 2.0)	127.1
20 CH	6.12 (dd, 15.8, 2.6)	133.3	6.15 (dd, 16.0, 3.0)	133.5
21 CH	5.44 (s)	77.8	5.49 (m)	78.4
23 C		169.4		170.4
24 CH_3	2.19 (s)	20.7	2.23 (s)	20.5
25 CH_3	1.16 (d, 7.6)	19.3	1.19 (d, 7.5)	19.4
26 CH_3	1.48 (s)	24.0	1.50 (s)	25.3
1′ C		137.8		138.4
2′, 6′ CH	7.16 (d, 7.2)	129.3	7.10-7.36 (m)	130.9
3′, 5′ CH	7.32 (t, 7.4)	128.8	7.10-7.36 (m)	129.0
4′ CH	7.24 (t, 7.6)	126.9	7.10-7.36 (m)	127.5

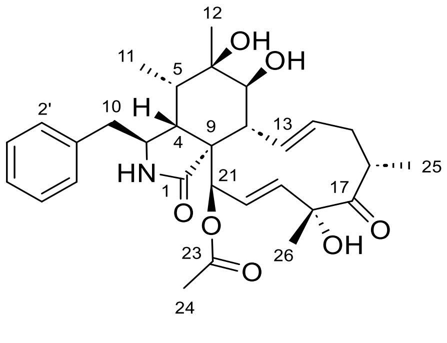

Figure S43 Structure of cytochalasin O$_{hyp}$ (**9**)

Table S11 ^{1}H and ^{13}C NMR data of compound **10** (400 MHz, pyridine-d_5) and cytochalasin C (400 MHz, pyridine-d_5)

Position/ DEPT	**10**		**Cytochalasin C** [7]	
	δ_H	δ_C	δ_H	δ_C
1 C		175.7		175.7
2 NH				
3 CH	3.69 (t, 7.2)	61.1	3.69 (ddt, 7.5, 7.5, 1.5)	61.1
4 CH	2.88 (brs)	50.8	2.88 (d, 1.5)	50.7
5 C		126.7		126.7
6 C		134.1		134.1
7 CH	4.48 (brd, 9.2)	69.2	4.40-4.51 (m)	69.1
8 CH	3.09 (t, 10.0)	50.8	3.09 (dd, 10.0, 10.0)	50.7
9 C		53.9		53.8
10 CH_2	3.23 (dd, 13.2, 7.6), 3.16 (dd, 13.2, 7.6)	45.5	3.22 (dd, 13.0, 7.5), 3.16 (dd, 13.0, 7.5)	45.3
11 CH_3	1.95 (s)	15.0	1.95 (s)	14.9
12 CH_3	1.40 (s)	17.4	1.41 (s)	17.3
13 CH	6.59 (dd, 15.6, 10.0)	132.8	6.59 (ddd, 15.4, 10.0, 1.0)	132.7
14 CH	5.56 (ddd, 15.4, 10.4, 5.0)	133.6	5.54 (ddd, 15.4, 10.0, 5.5)	133.5
15 CH_2	2.61-2.76 (m), 1.95-1.99 (m)	38.8	2.60-2.75 (m), 1.93-1.96 (m)	38.7
16 CH	2.61-2.76 (m)	42.6	2.60-2.75 (m)	42.5
17 C		210.9		210.9
18 C		78.7		78.6
19 CH	5.66 (dd, 15.8, 1.8)	128.6	5.66 (dd, 15.5, 2.5)	128.6
20 CH	6.82 (dd, 16.0, 1.8)	133.0	6.84 (dd, 15.5, 2.5)	132.9
21 CH	6.41 (m)	76.5	6.41 (dd, 2.5, 2.5)	76.4
23 C		170.9		170.9
24 CH_3	2.38 (s)	20.8	2.38 (s)	20.7
25 CH_3	1.06 (d, 6.4)	19.6	1.05 (d, 6.3)	19.5
26 CH_3	1.59 (s)	24.9	1.58 (s)	24.8
1′ C		139.0		139.0
2′, 6′ CH	7.25-7.34 (m)	130.0	7.22-7.34 (m)	130.0
3′, 5′ CH	7.25-7.34 (m)	129.1	7.22-7.34 (m)	129.1
4′ CH	7.25-7.34 (m)	127.1	7.22-7.34 (m)	127.0

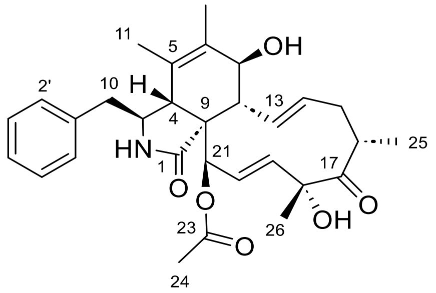

Figure S44 Structure of cytochalasin C (**10**)

Table S12 ^{1}H and ^{13}C NMR data of compound **11** (400 MHz, $CDCl_3$) and 2-chloro-5-methoxy-3-methylcyclohexa-2,5-diene-1,4-dione (400 MHz, $CDCl_3$)

Position	**11**		**2-chloro-5-methoxy-3-methylcyclohexa-2,5-diene-1,4-dione** [8]	
	δ_H	δ_C	δ_H	δ_C
1		179.0		179.1
2		140.1		140.1
3		141.6		141.5
4		179.6		179.6
5		158.8		158.8
6	6.02 (s)	106.7	5.97 (s)	106.6
3-CH_3	2.20 (s)	13.4	2.14 (s)	13.4
5-OCH_3	3.84 (s)	56.5	3.80 (s)	56.6

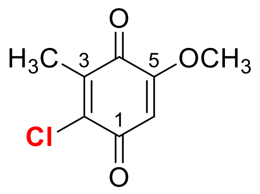

Figure S45 Structure of 2-chloro-5-methoxy-3-methylcyclohexa-2,5-diene-1,4-dione (**11**)

Table S13 ^{1}H and ^{13}C NMR data of compound **12** (500 MHz, $CDCl_3$) and 2-hydroxy-5-methoxy-3-methylcyclohexa-2,5-diene-1,4-dione (400 MHz, $CDCl_3$)

Position	**12**		**2-hydroxy-5-methoxy-3-methylcyclohexa-2,5-diene-1,4-dione** [9]	
	δ_H	δ_C	δ_H	δ_C
1		182.6		184.5
2		151.7		151.6
3		114.9		114.8
4		182.0		182.0
5		161.2		161.2
6	5.84 (s)	102.2	5.84 (s)	102.1
3-CH_3	1.95 (s)	7.8	1.94 (s)	7.8
5-OCH_3	3.86 (s)	56.8	3.86 (s)	56.8

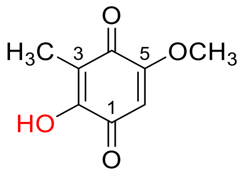

Figure S46 Structure of 2-hydroxy-5-methoxy-3-methylcyclohexa-2,5-diene-1,4-dione (**12**)

Table S14 ^{1}H and ^{13}C NMR spectral data of compound **13** (400 MHz, CD3OD) and 4-hydroxymethylbenzoate (500 MHz, DMSO-d_6)

Position	**13**		**4-hydroxymethylbenzoate** [10]	
	***δ*H**	***δ*C**	***δ*H**	***δ*C**
1		155.9		157.2
2	6.87 (d, 3.2)	114.0	7.22	122.0
3	6.33 (d, 3.6)	108.3	6.57	111.9
4		150.3		152.8
5	6.33 (d, 3.6)	108.3	6.57	111.9
6	6.87 (d, 3.2)	114.0	7.22	122.0
7		165.5		177.0
8	4.52 (brs)	56.2	4.64 (s)	64.6

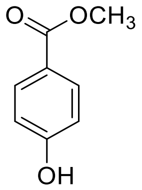

Figure S47. Structure of 4-hydroxymethylbenzoate (**13**)

Table S15 ^{1}H and ^{13}C NMR data of compound **14** (400 MHz, acetone-d_6) and (4*R*,5*S*,6*R*)-4,5,6-trihydroxy-3-methoxy-5-methyl-cyclohex-2-en-1-one (400 MHz, acetone-d_6)

Position	**14**		**(4*R*,5*S*,6*R*)-4,5,6-trihydroxy-3-methoxy-5-methyl-cyclohex-2-en-1-one** [8]	
	***δ*H**	***δ*C**	***δ*H**	***δ*C**
1		196.7		190.6
2	5.38 (d, 1.2)	99.0	5.31 (s)	100.6
3		175.4		175.8
4	4.05 (d, 1.2)	74.3	4.23 (s)	73.8
5		75.4		75.6
6	4.30 (s)	74.6	4.44 (brs)	67.6
7	1.08 (s)	18.0	1.27 (s)	21.8
8	3.80 (s)	56.2	3.75 (s)	57.5

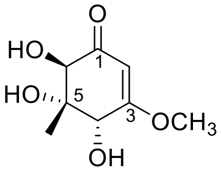

Figure S48 Structure of (4*R*,5*S*,6*R*)-4,5,6-trihydroxy-3-methoxy-5-methyl-cyclohex-2-n-1-one (**14**)

Table S16 ^{1}H, ^{13}C, 2D NMR and NOE data of compound **15** (500 MHz, $CDCl_3$)

**Position /DEPT**	δ_H	δ_C	**COSY**	**HMBC**	**NOE**
1 C		164.4			
2 O					
3 CH	4.65 (1H, br t, 10.5)	74.3	H-4, 9		-
4 CH_2	2.76 (1H, dd, 16.0, 11.4)<br>2.67 (1H, d, 16.0)	35.1	H-3	C-3, 4a, 5, 8a	
4a C		144.2			
5 CH	6.26 (1H, s)	106.8		C-4, 7, 8a	
6 C		162.9			
7 CH	6.40 (1H, s)	98.7		C-5, 6, 8a	
8 C		163.5			
8a C		105.4			
9 CH_2	1.84 (1H, m)<br>1.71 (1H, m)	41.6	H-3, 2′	C-3, 2′	
1′ O					
2′ CH	3.67 (1H, t, 10.7)	73.3	H-9, 3′		H-6′
3′ CH_2	1.55 (1H, m)<br>1.19 (1H, m)	31.7	H-2′		
4′ CH_2	1.81 (1H, m)<br>1.53 (1H, m)	23.5	H-5′	C-5′	
5′ CH_2	1.55 (1H, m)<br>1.19 (1H m)	33.2	H-4′		
6′ CH	3.43 (1H, m)	74.0	6′-CH_3		H-2′<br>6′-CH_3
6′-CH_3	1.13 (d, 6.0)	22.0	H-6′	C-5′, 6′	H-6′
8-OCH_3	3.80 (s)	55.8		C-8	

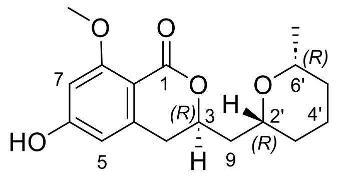

Figure S49 Structure of (3*R*,2′*R*,6′*R*)-asperentin-8-*O*-methylether (**15**)

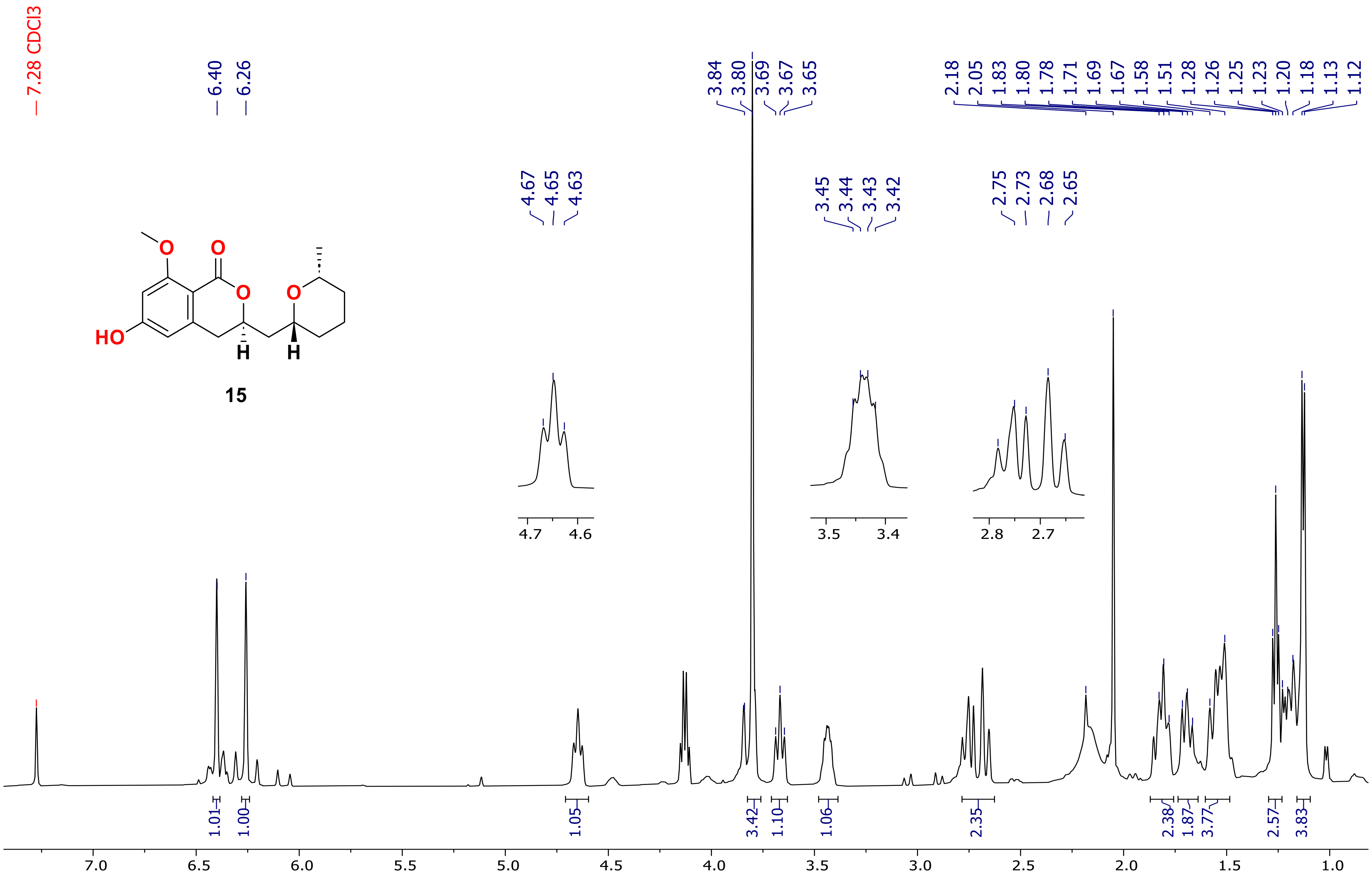

Figure S50 ^{1}H NMR spectrum of **15** (500 MHz, $CDCl_3$)

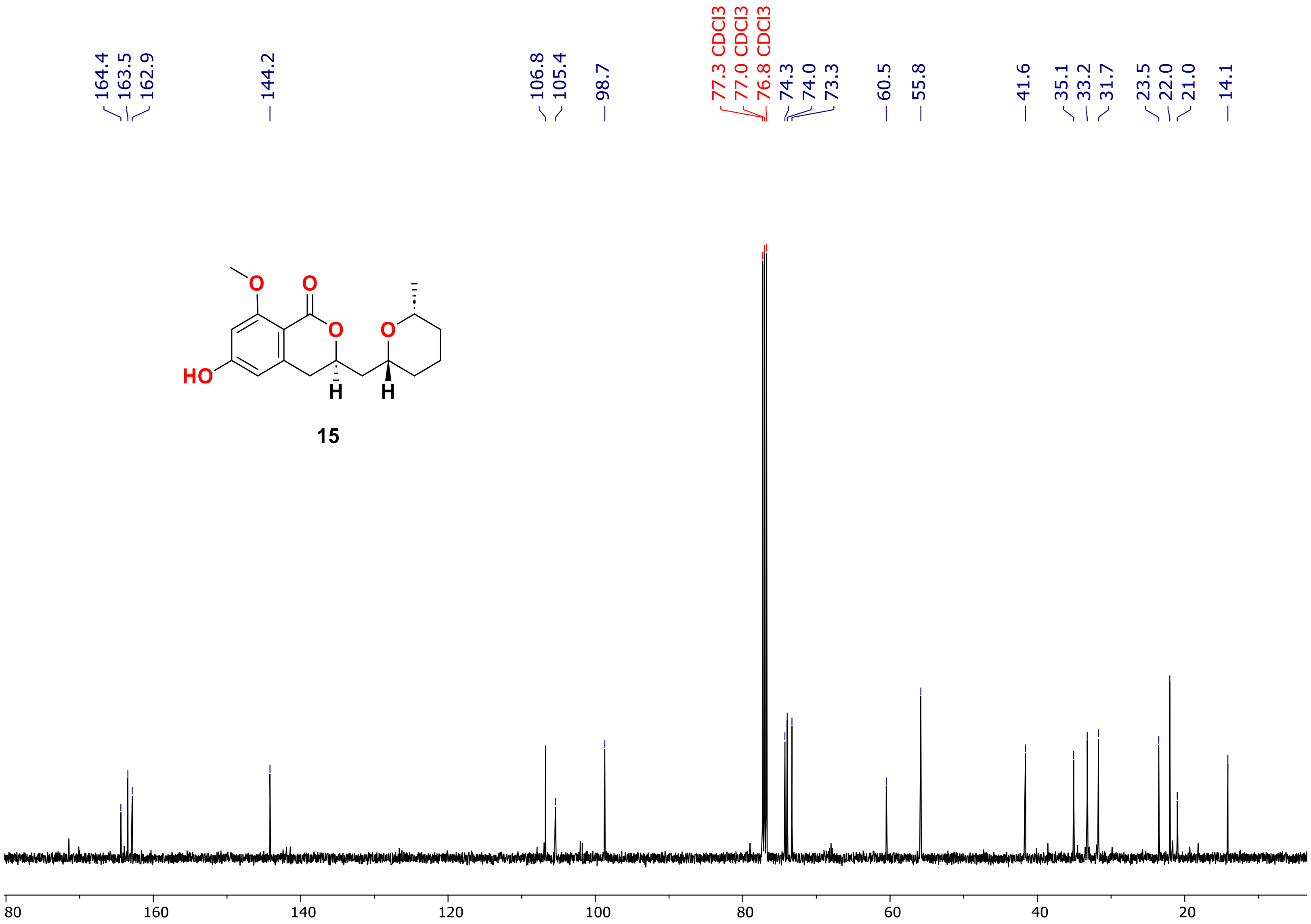

Figure S51 ^{13}C NMR spectrum of **15** (125 MHz, $CDCl_3$)

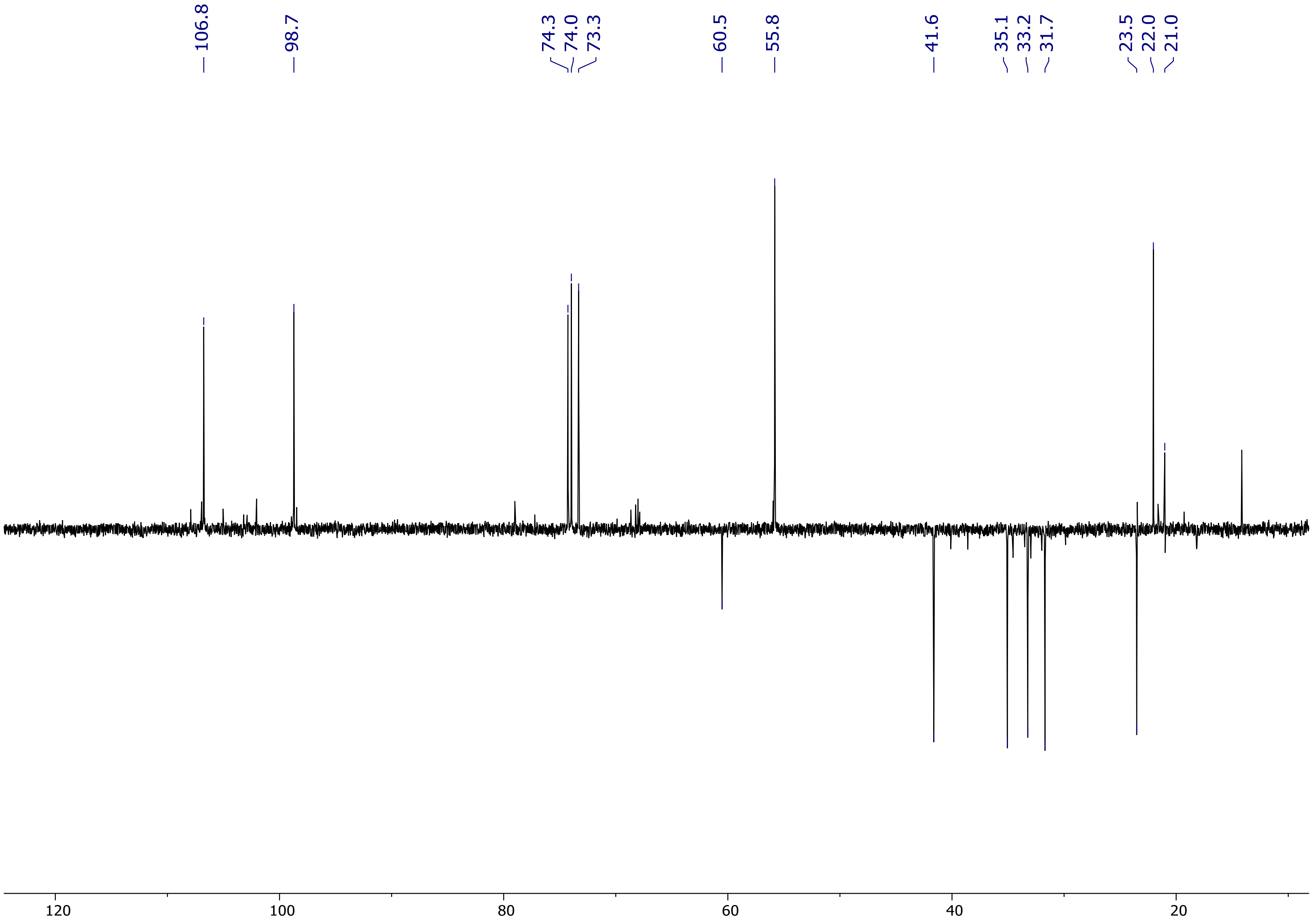

Figure S52 DEPT135 spectrum of **15**

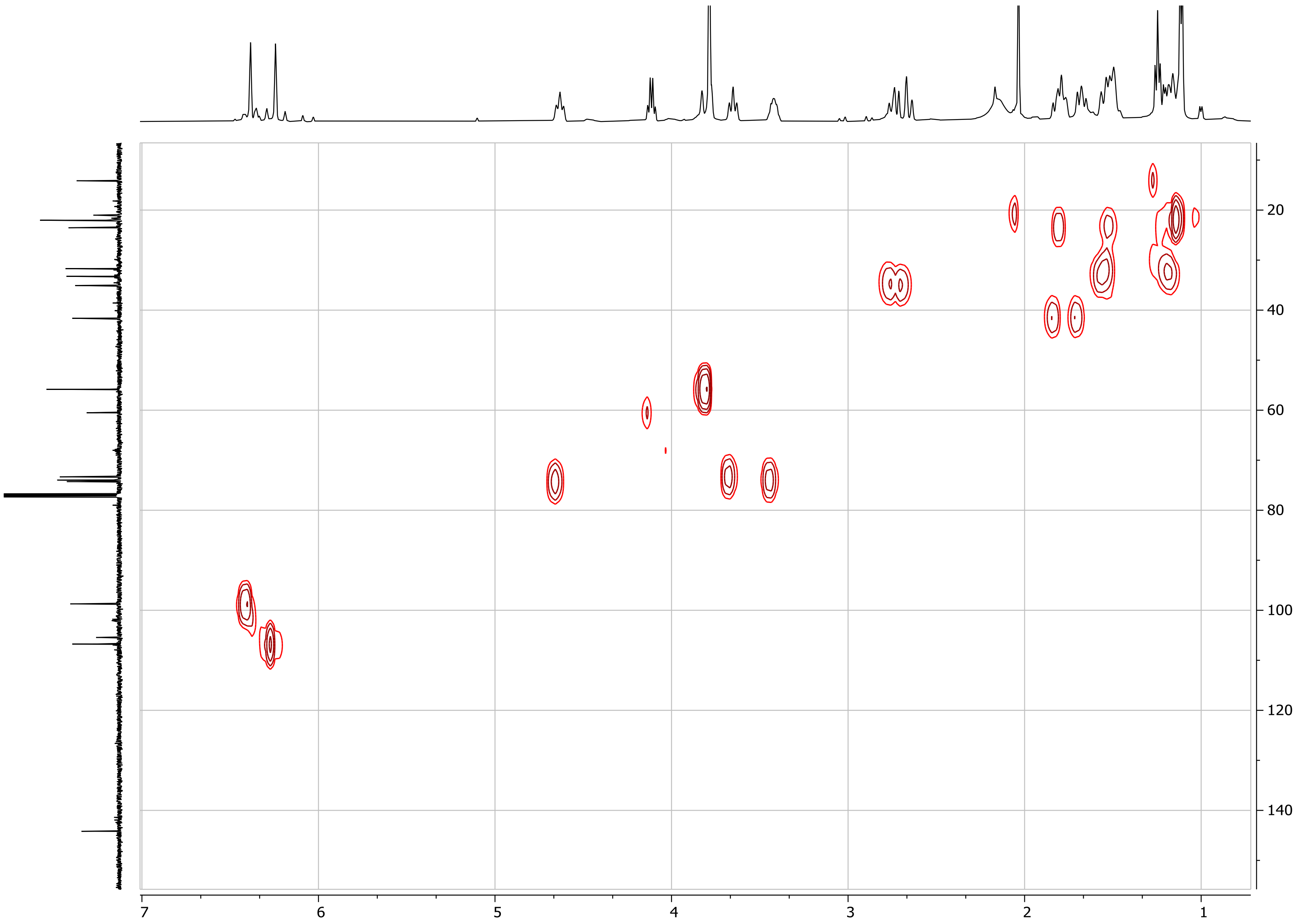

Figure S53 HSQC spectrum of **15**

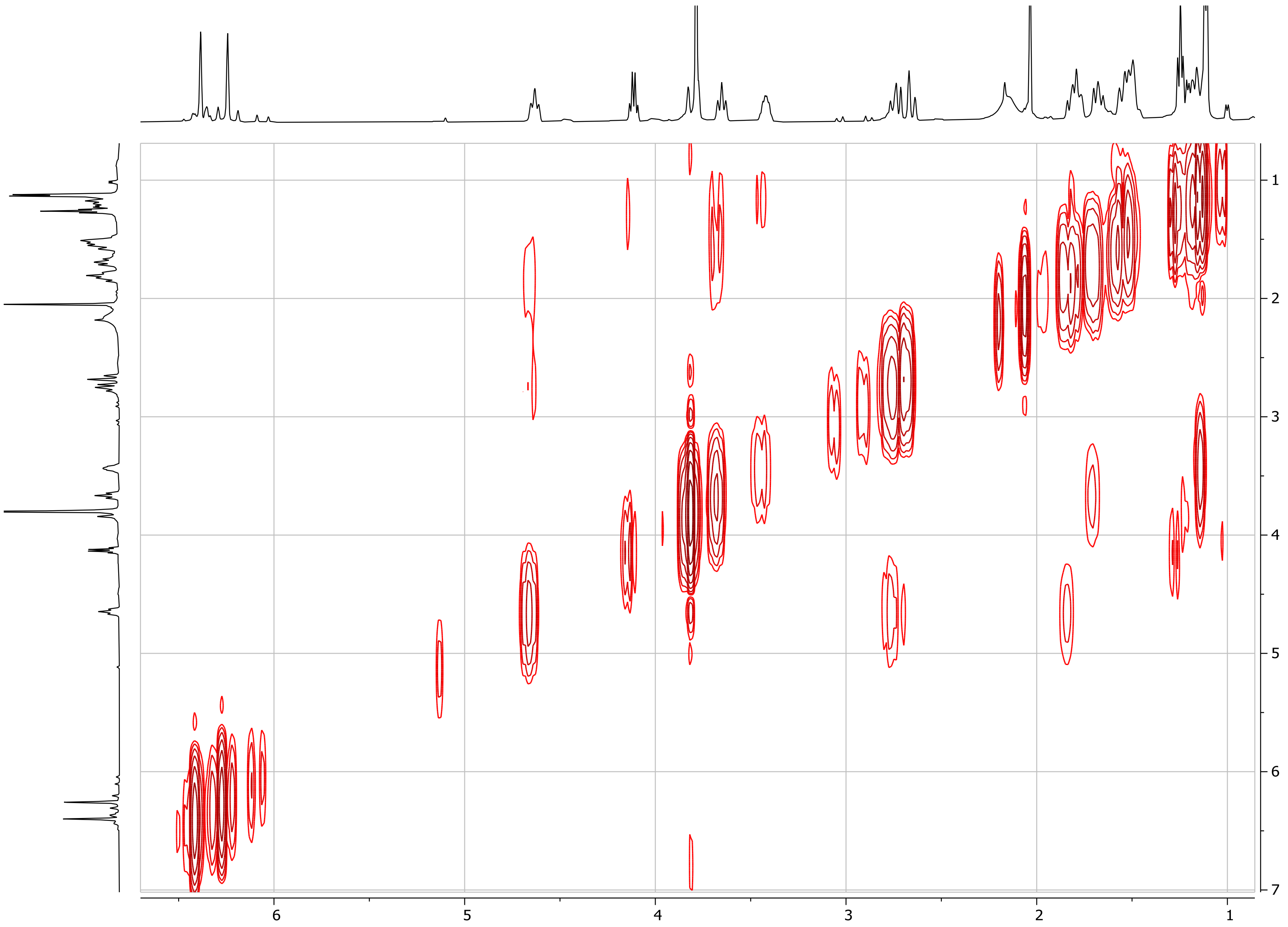

Figure S54 COSY spectrum of **15**

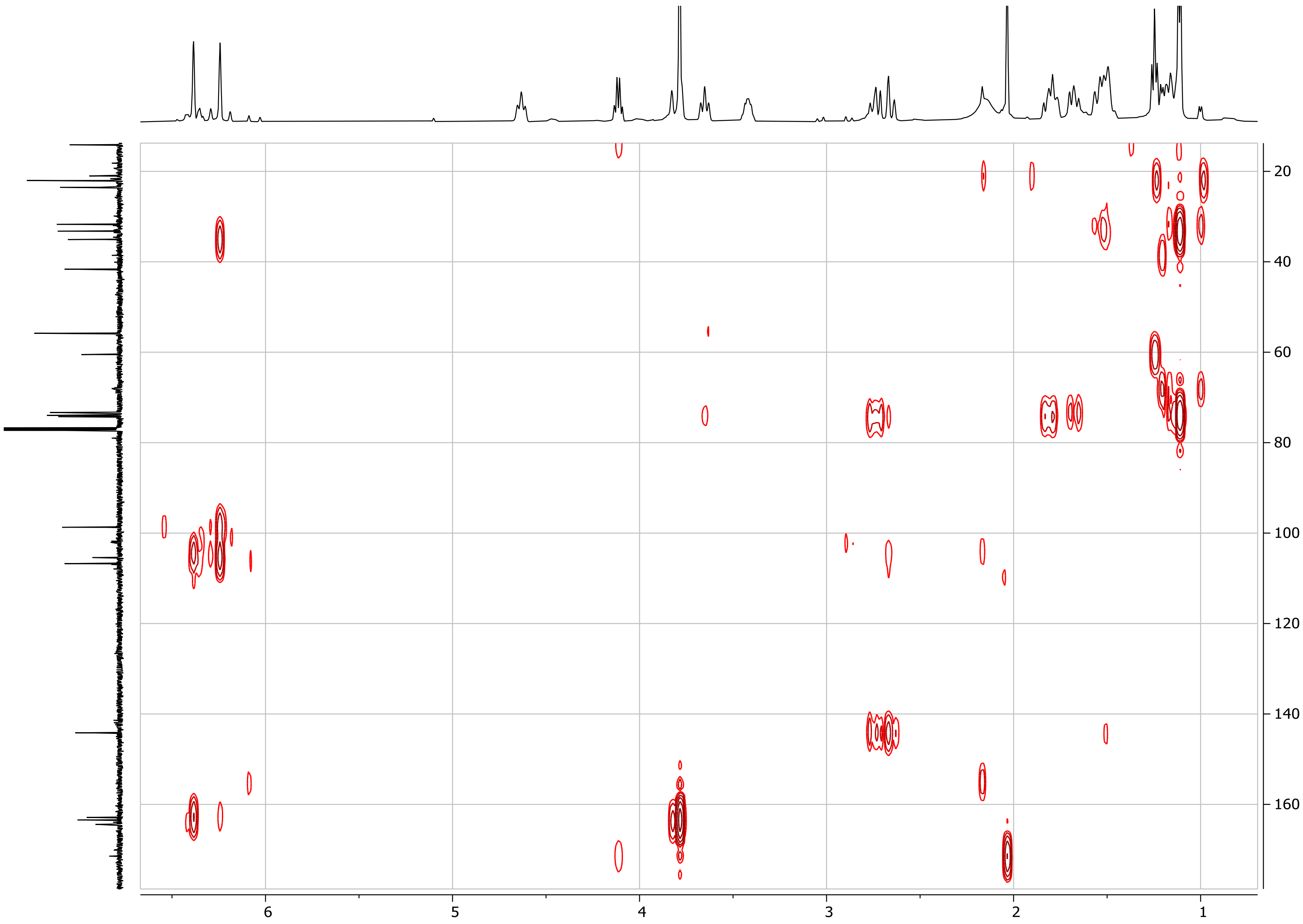

Figure S55 HMBC spectrum of **15**

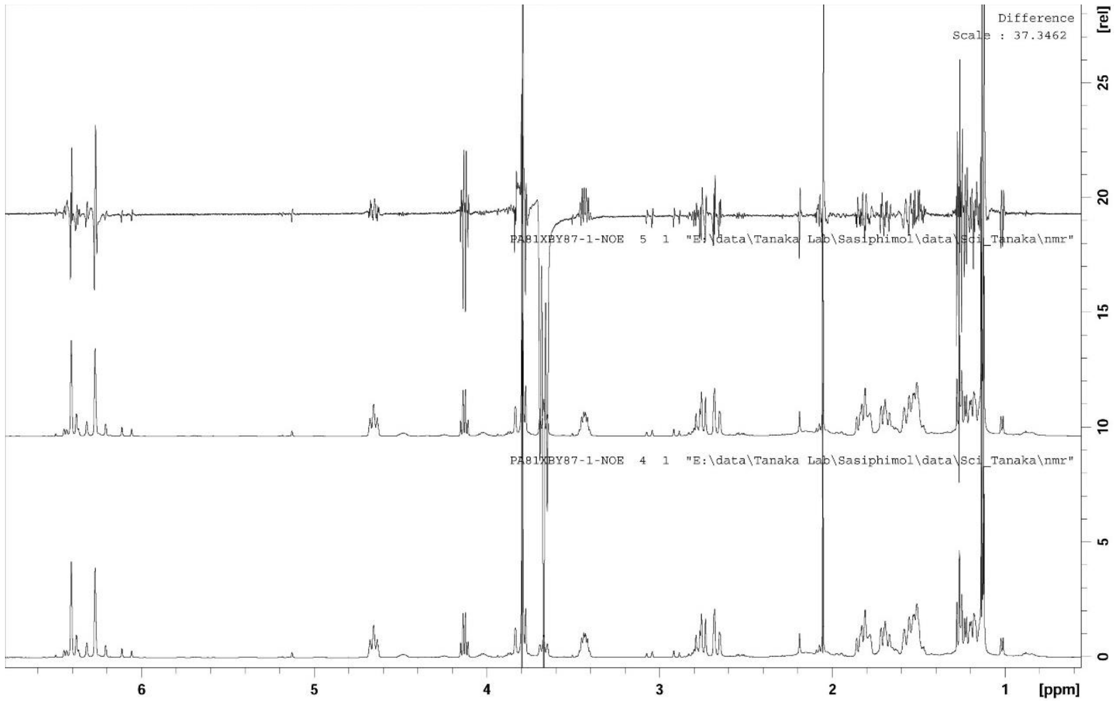

Figure S56 NOE (H-2′) spectrum of **15**

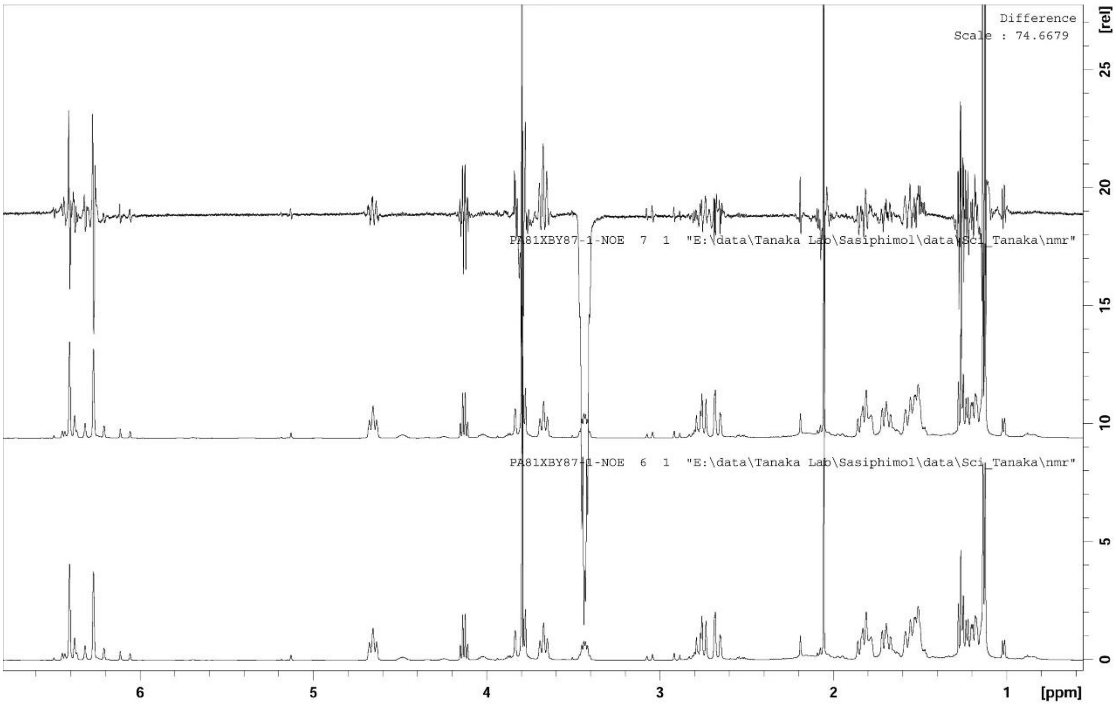

Figure S57 NOE (H-6′) spectrum of **15**

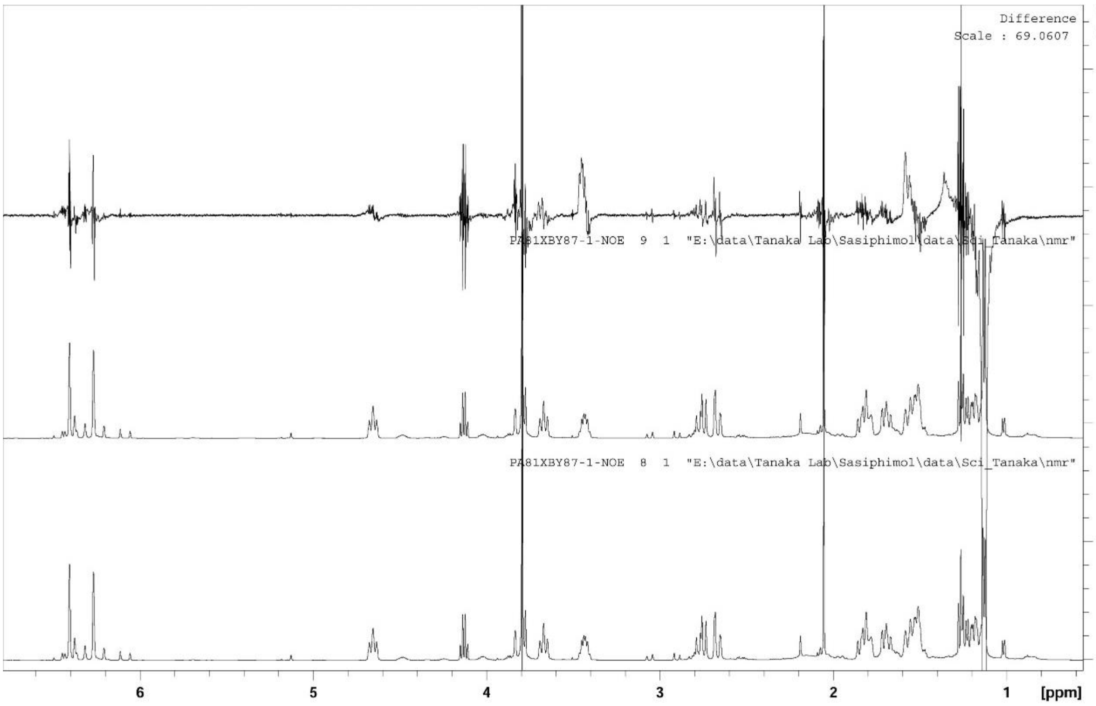

Figure S58 NOE (C-6′-CH_3) spectrum of **15**

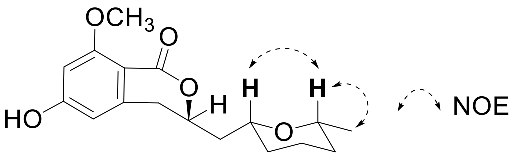

15

Figure S59 NOE correlations of compounds **15**

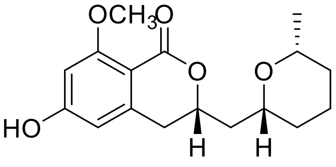

(3*S**, 2'*R**, 6'*R**)-**15a**

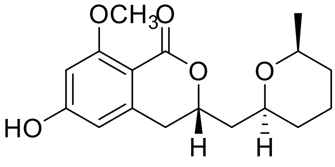

(3*S**, 2'*S**, 6'*S**)-**15c**

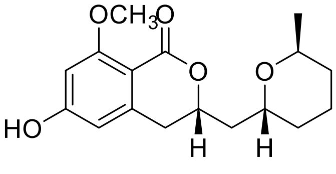

(3*S**, 2'*R**, 6'*S**)-**15e**

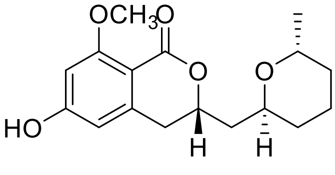

(3*S**, 2'*S**, 6'*R**)-**15g**

Figure S60 Structures of **15a**, **15c**, **15e** and **15g** diastereomers used for ^{13}C NMR chemical shift calculations

Table S17 Experimental ^{13}C NMR chemical shifts of **15** and the calculated Boltzmann averaged shielding values of **15a**, **15c**, **15e**, and **15g** diastereomers

Position	Expt. δ_C of **15**	Boltzmann averaged shielding values			
		**15a**	**15c**	**15e**	**15g**
1	164.4	37.85	37.50	37.58	38.05
3	74.3	117.91	117.73	115.48	117.83
4	35.1	159.23	157.94	154.17	157.38
4a	144.2	50.46	51.74	51.98	51.33
5	106.8	89.94	91.31	90.32	91.14
6	162.9	36.92	37.46	36.64	37.12
7	98.7	99.32	99.03	98.66	98.82
8	163.5	34.30	34.24	34.02	34.15
8a	105.4	86.66	85.10	86.24	85.46
9	41.6	147.16	147.56	155.23	146.70
2'	73.3	119.17	118.41	118.14	125.71
3'	31.7	158.46	158.46	162.20	157.49
4'	23.5	166.28	166.48	170.07	171.09
5'	33.2	158.44	158.60	158.23	160.81
6'	74.0	119.08	118.97	125.57	122.59
6'-CH_3	22.0	169.00	169.67	169.19	175.37
8-OCH_3	55.8	138.64	138.53	138.63	138.67

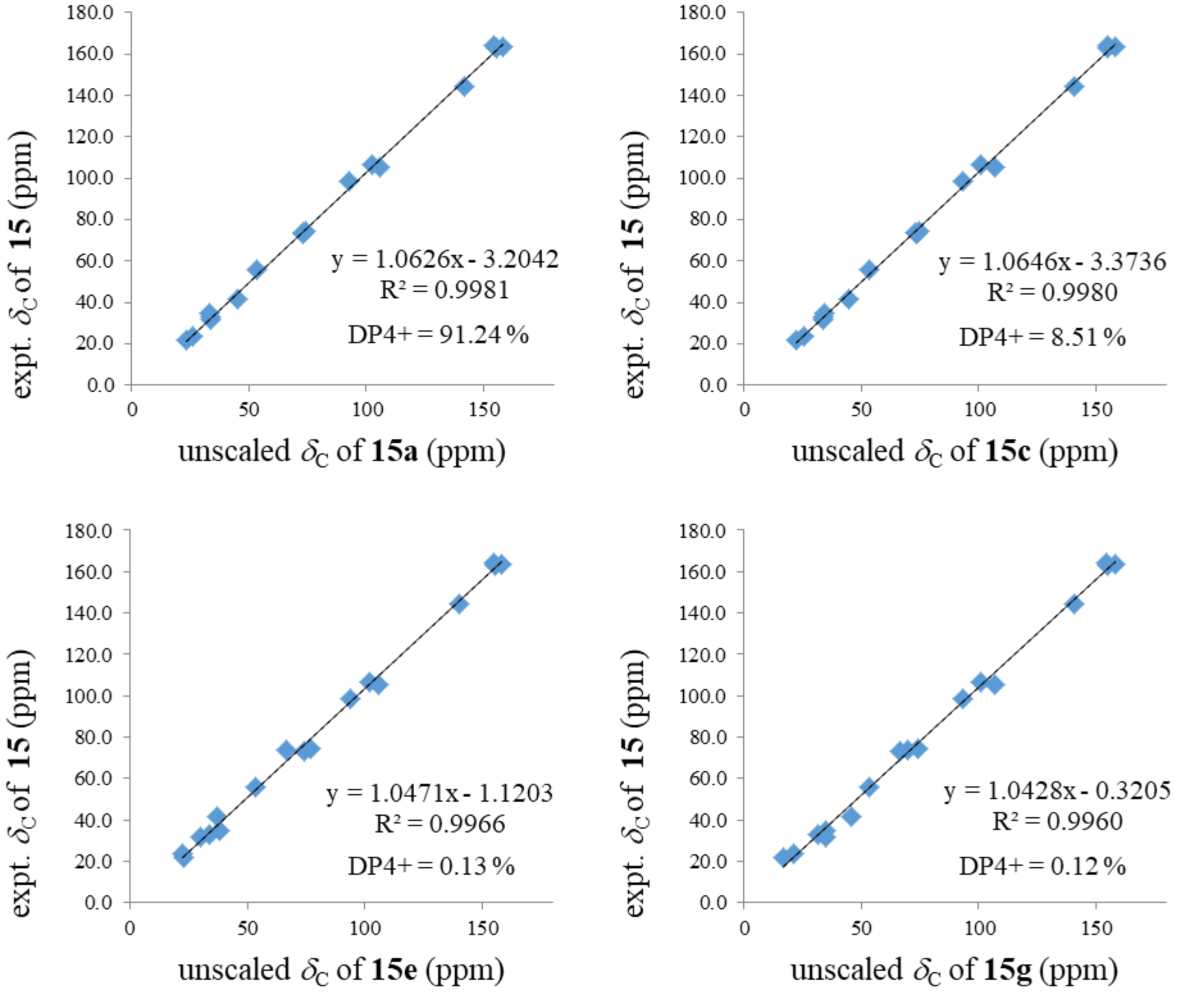

Figure S61 Linear correlations between unscaled and experimental ^{13}C NMR chemical shifts of **15a**, **15c**, **15e**, and **15g** diastereomers

Functional	Solvent?	Basis Set	Type of Data
B3LYP	PCM	6-31G(d,p)	Shielding Tensors

Nuclei	sp2?	Experimental	Isomer 1	Isomer 2	Isomer 3	Isomer 4	Isomer 5	Isomer 6	Isomer 7	Isomer 8	Isomer 9	Isomer 10	Isomer 11	Isomer 12
		DP4+	91.24%	8.51%	0.13%	0.12%	-	-	-	-	-	-	-	-
c		164.4	37.85	37.50	37.58	38.05								
c	x	74.3	117.91	117.73	115.48	117.83								
c	x	35.1	159.23	157.94	154.17	157.38								
c		144.2	50.46	51.74	51.98	51.33								
c		106.8	89.94	91.31	90.32	91.14								
c		162.9	36.92	37.46	36.64	37.12								
c		98.7	99.32	99.03	98.66	98.82								
c		163.5	34.30	34.24	34.02	34.15								
c		105.4	86.66	85.10	86.24	85.46								
c	x	41.6	147.16	147.56	155.23	146.70								
c	x	73.3	119.17	118.41	118.14	125.71								
c	x	31.7	158.46	158.46	162.20	157.49								
c	x	23.5	166.28	166.48	170.07	171.09								
c	x	33.2	158.44	158.60	158.23	160.81								
c	x	74.0	119.08	118.97	125.57	122.59								
c	x	22.0	169.00	169.67	169.19	175.37								
c	x	55.8	138.64	138.53	138.63	138.67								

Main | Detailed Results

Functional	Solvent?	Basis Set	Type of Data
B3LYP	PCM	6-31G(d,p)	Shielding Tensors

	Isomer 1	Isomer 2	Isomer 3	Isomer 4	Isomer 5	Isomer 6	Isomer 7	Isomer 8	Isomer 9	Isomer 10	Isomer 11	Isomer 12	Isomer 13
sDP4+ (H data)	-	-	-	-	-	-	-	-	-	-	-	-	-
sDP4+ (C data)	71.38%	28.60%	0.02%	0.00%	-	-	-	-	-	-	-	-	-
sDP4+ (all data)	71.38%	28.60%	0.02%	0.00%	-	-	-	-	-	-	-	-	-
uDP4+ (H data)	-	-	-	-	-	-	-	-	-	-	-	-	-
uDP4+ (C data)	0.98%	0.23%	4.94%	93.86%	-	-	-	-	-	-	-	-	-
uDP4+ (all data)	0.98%	0.23%	4.94%	93.86%	-	-	-	-	-	-	-	-	-
DP4+ (H data)	-	-	-	-	-	-	-	-	-	-	-	-	-
DP4+ (C data)	91.24%	8.51%	0.13%	0.12%	-	-	-	-	-	-	-	-	-
DP4+ (all data)	91.24%	8.51%	0.13%	0.12%	-	-	-	-	-	-	-	-	-

Main | Detailed Results

Figure S62 The results of DP4+ analysis of **15a**, **15c**, **15e**, and **15g** diastereomers

Table S 18 The results of energy analysis for conformers of **15aa-ac**

conformer	Energy (Hartree)[a]	% population[b]
**15aa**	1037.225911	57.53
**15ab**	1037.228531	3.59
**15ac**	1037.226281	38.88

[a] calculated using B3LYP/6-31G(d,p) level at 298.15 K

[b] calculated using %Boltzmann distribution = $\frac{e^{-E_i/RT}}{\sum_i e^{-E_i/RT}}$ x 100

Table S19 Cartesian coordinates for the low-energy optimized conformers of **15aa-ac**

Conformer **15aa**				
Tag	*Symbol*	*X*	*Y*	*Z*
1	C	-2.506163	1.466639	0.252971
2	C	-1.893551	2.020680	-0.875784
3	C	-1.071862	1.246187	-1.692444
4	C	-0.866824	-0.095111	-1.382885
5	C	-1.487238	-0.693287	-0.269401
6	C	-2.309673	0.116262	0.563795
7	C	-0.021269	-0.951613	-2.291077
8	C	0.605673	-2.152851	-1.578962
9	O	-0.349880	-2.809741	-0.717232
10	C	-1.324134	-2.149371	-0.016041
11	C	1.930560	-1.934106	-0.830036
12	C	2.019134	-0.828546	0.222309
13	C	3.211488	-1.032265	1.167664
14	C	3.356624	0.162082	2.120042
15	C	3.384125	1.475555	1.327198
16	C	2.189084	1.563991	0.368971
17	O	2.144035	0.411949	-0.482285
18	O	-2.002507	-2.808449	0.738881
19	O	-2.061602	3.329998	-1.216787
20	H	0.818559	-2.915640	-2.335852
21	H	1.098017	-0.803048	0.828364
22	C	2.245352	2.785095	-0.538698
23	O	-2.862456	-0.462180	1.651863
24	C	-3.696243	0.309074	2.501491
25	H	-3.129965	2.093597	0.880927
26	H	-0.601634	1.700196	-2.558233
27	H	0.765566	-0.346613	-2.750359
28	H	-0.658292	-1.328198	-3.104062
29	H	2.714761	-1.742516	-1.574767
30	H	2.170311	-2.892277	-0.353358
31	H	4.123758	-1.143988	0.564652
32	H	3.075870	-1.963795	1.731442
33	H	2.505355	0.174358	2.816276
34	H	4.260818	0.060540	2.732421
35	H	4.308056	1.534242	0.734899
36	H	3.376834	2.339504	2.004459
37	H	1.258650	1.593167	0.963762
38	H	-2.655353	3.753744	-0.577372
39	H	2.281124	3.704053	0.057125
40	H	3.139719	2.747615	-1.170814
41	H	1.364269	2.825827	-1.185148

42	H	-4.573895	0.693120	1.965352
43	H	-4.023610	-0.374632	3.286001
44	H	-3.150155	1.147636	2.954077

Conformer **15ab**

Tag	Symbol	X	Y	Z
1	C	-3.252882	0.714647	-0.777340
2	C	-3.589586	-0.602819	-1.100472
3	C	-2.872755	-1.674096	-0.565491
4	C	-1.821502	-1.420243	0.309306
5	C	-1.464428	-0.104936	0.669054
6	C	-2.194475	0.973104	0.102950
7	C	-1.023792	-2.554798	0.907221
8	C	0.427783	-2.119354	1.101586
9	O	0.455690	-0.931811	1.919274
10	C	-0.377272	0.124678	1.659003
11	C	1.198931	-1.953872	-0.223183
12	C	2.436979	-1.058306	-0.123468
13	C	3.501795	-1.348094	-1.188481
14	C	4.641278	-0.320598	-1.087503
15	C	4.088910	1.114241	-1.085034
16	C	2.973197	1.272219	-0.043007
17	O	1.965000	0.281131	-0.265461
18	O	-0.207720	1.141818	2.292219
19	O	-4.612507	-0.897432	-1.951966
20	H	0.954724	-2.852877	1.720463
21	H	2.898006	-1.169571	0.873836
22	C	2.274934	2.624106	-0.085983
23	O	-1.814838	2.226762	0.431802
24	C	-2.510743	3.331477	-0.122548
25	H	-3.815980	1.529988	-1.218798
26	H	-3.144776	-2.687179	-0.843093
27	H	-1.442225	-2.834232	1.884070
28	H	-1.077264	-3.443471	0.268036
29	H	0.553369	-1.528714	-0.999622
30	H	1.488586	-2.958615	-0.558479
31	H	3.031018	-1.293719	-2.179944
32	H	3.889691	-2.368013	-1.064982
33	H	5.197614	-0.495098	-0.154766
34	H	5.357043	-0.456800	-1.907563
35	H	3.672935	1.354703	-2.073436
36	H	4.891643	1.836153	-0.885672
37	H	3.398100	1.115234	0.964251
38	H	-5.023666	-0.072869	-2.255286
39	H	2.982467	3.429772	0.140884
40	H	1.853438	2.803447	-1.082097

41	H	1.461255	2.646264	0.644731
42	H	-2.433797	3.351239	-1.217794
43	H	-3.569313	3.328991	0.168765
44	H	-2.026095	4.217501	0.289889

Conformer **15ac**

Tag	Symbol	X	Y	Z
1	C	-2.511222	1.467016	0.276188
2	C	-1.907184	2.028149	-0.851945
3	C	-1.086923	1.260443	-1.677880
4	C	-0.874507	-0.084842	-1.379402
5	C	-1.487975	-0.688334	-0.268378
6	C	-2.308534	0.117957	0.575662
7	C	-0.030468	-0.931419	-2.298197
8	C	0.598497	-2.139095	-1.598388
9	O	-0.354386	-2.802639	-0.740329
10	C	-1.324142	-2.146223	-0.027132
11	C	1.926002	-1.927135	-0.851857
12	C	2.017272	-0.832510	0.211486
13	C	3.209203	-1.046543	1.154882
14	C	3.353310	0.137439	2.120311
15	C	3.382745	1.459398	1.341693
16	C	2.189742	1.557948	0.382085
17	O	2.144424	0.415187	-0.481310
18	O	-1.998065	-2.811370	0.725871
19	O	-2.155130	3.348243	-1.086236
20	H	0.809305	-2.894750	-2.363047
21	H	1.096472	-0.810708	0.817754
22	C	2.251649	2.787167	-0.514281
23	O	-2.852254	-0.469639	1.662294
24	C	-3.682914	0.298102	2.520575
25	H	-3.128999	2.104399	0.894659
26	H	-0.610532	1.705360	-2.548998
27	H	-0.668300	-1.300836	-3.113899
28	H	0.756257	-0.322383	-2.752921
29	H	2.708167	-1.728180	-1.596856
30	H	2.167062	-2.890036	-0.385581
31	H	4.121879	-1.151995	0.551334
32	H	3.072921	-1.983932	1.708595
33	H	2.501160	0.142657	2.815371
34	H	4.256676	0.029282	2.732661
35	H	4.308075	1.523925	0.752121
36	H	3.373911	2.316025	2.028075
37	H	1.257829	1.584315	0.974038
38	H	-1.688094	3.618044	-1.892628
39	H	1.378741	2.823212	-1.172612

40	H	2.271609	3.701992	0.088275
41	H	3.153615	2.760643	-1.135998
42	H	-3.134732	1.135663	2.971299
43	H	-4.562479	0.683694	1.989681
44	H	-4.004564	-0.389749	3.303822

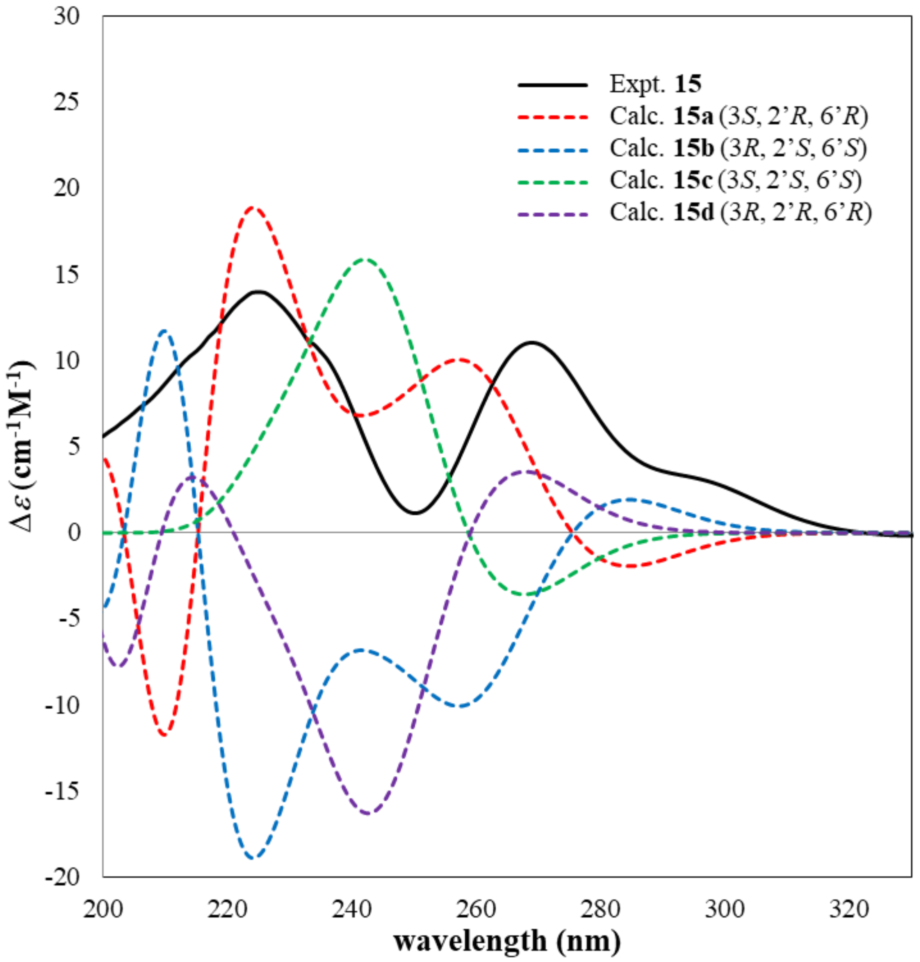

Figure S63 Comparison of experimental and calculated ECD spectra of **15**

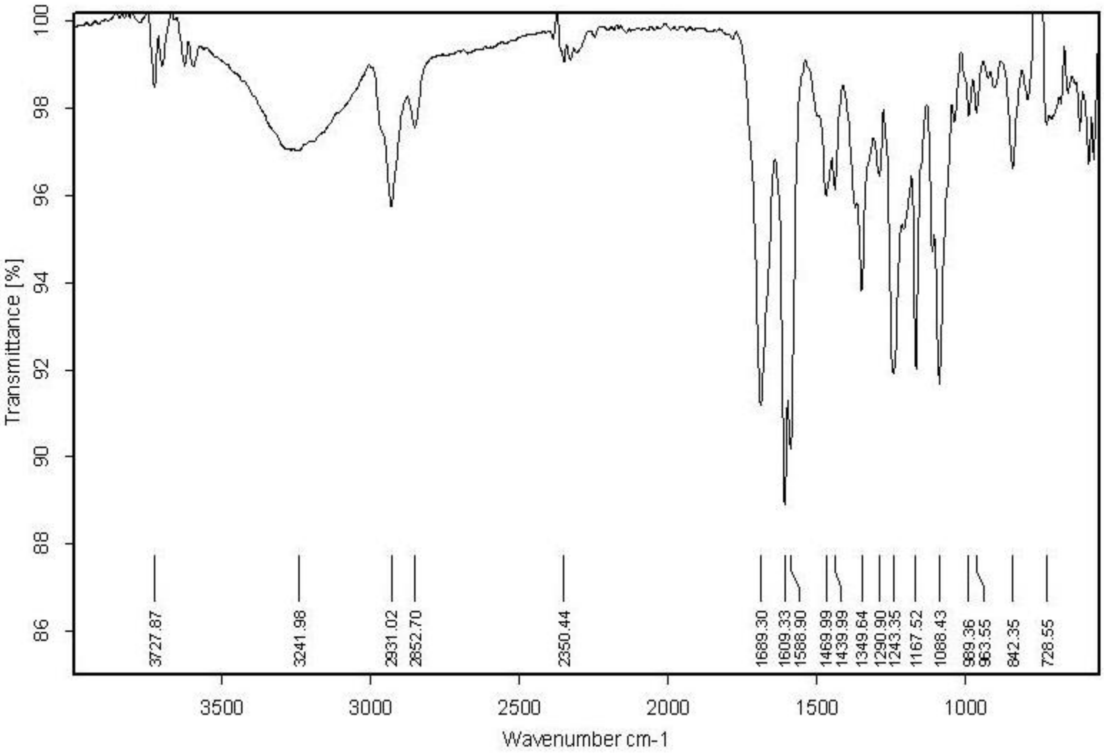

Figure S64 IR spectrum of **15**

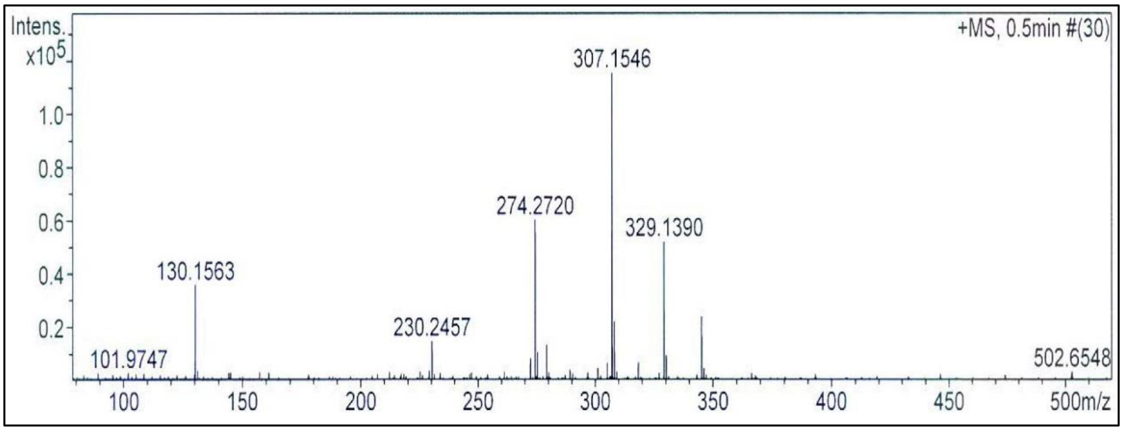

Figure S65 HRESIMS spectrum of **15**

Table S20 ^{1}H, ^{13}C, 2D NMR and NOE data of compound **16** (500 MHz, $CDCl_3$)

Position /DEPT		δ_H	δ_C	COSY	HMBC	NOE
1	O					
2	C		174.2			
3	CH	5.32 (s)	104.9		C-2, 5, 2-CH_3	
4	C		193.0			
5	CH_2	2.42 (d, 7.7),<br>2.40 (d, 1.6)	41.5	H-6	C-4, 6, 7	
6	CH	4.57 (m)	76.2	H-5, 7		H-2′
7	CH_2	1.98 (1H, m)<br>1.77 (1H, m)	38.5	H-6, 2′	C-6, 2′	
1′	O					
2′	CH	4.07 (m)	66.5	H-7, 3′		H-6, 6′-CH_3
3′	CH_2	1.69 (1H, m)<br>1.33 (1H, m)	30.4	H-4′	C-2′	
4′	CH_2	1.64 (1H, m)<br>1.69 (1H, m)	18.4	H-3′		
5′	CH_2	1.69 (1H, m)<br>1.33 (1H, m)	31.4	H-6′	C-3′, 6′	
6′	CH	3.87 (1H, pd, 6.4, 3.2)	67.0	H-5′, 6′-CH_3	6′-CH_3	6′-CH_3
2-CH_3		1.99 (s)	21.0		C-2, 3	
6′-CH_3		1.18 (d, 6.4)	19.5	H-6′	C-5′, 6′	H-2′, 6′

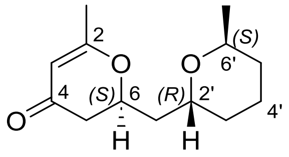

Figure S66 Structure of (6*S*,2′*R*,6′*S*)-6-methyl-2-((6-methyltetrahydro-2*H*-pyran-2-yl)methyl)-2,3-dihydro-4*H*-pyran-4-one (**16**)

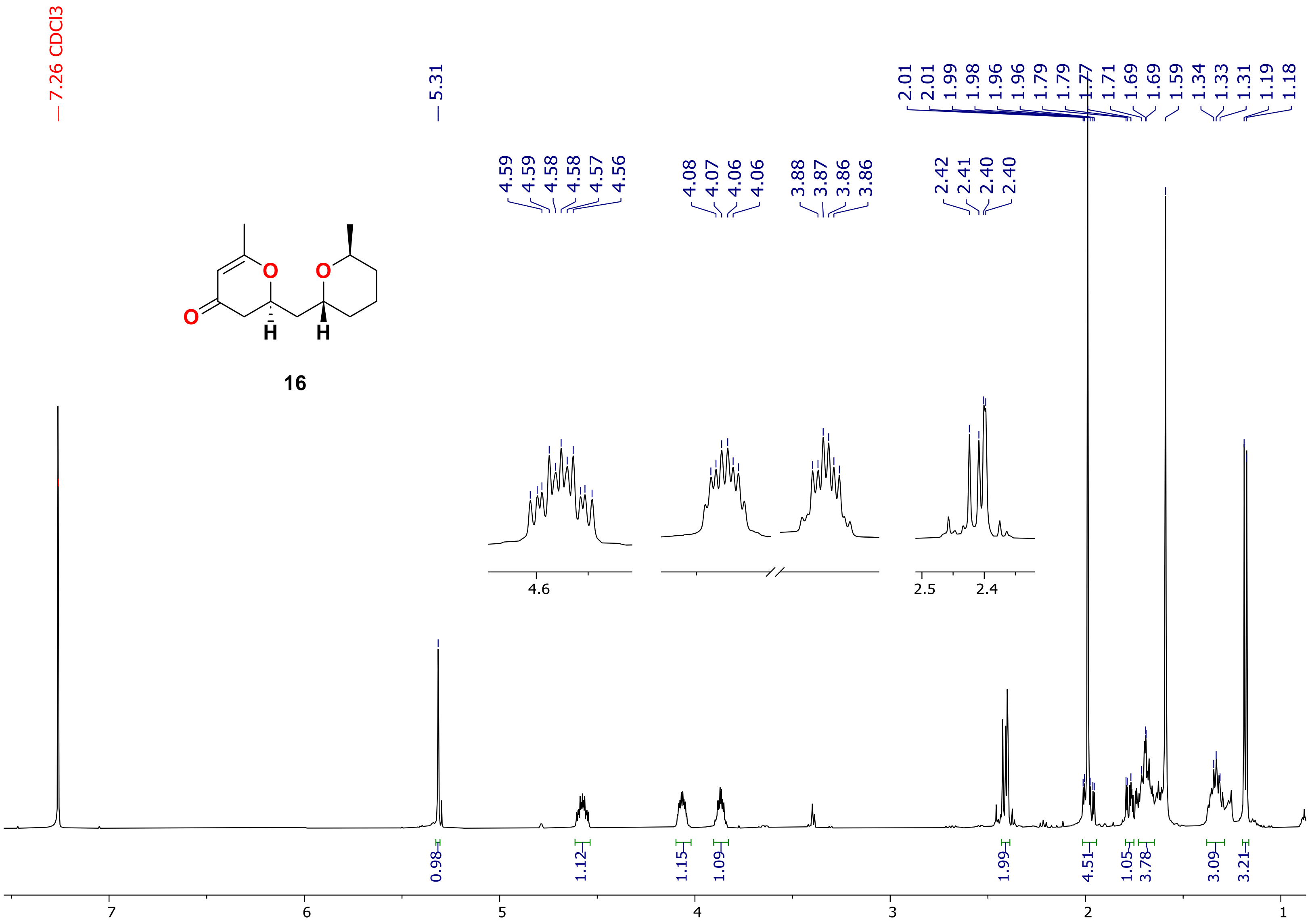

Figure S67 ¹H NMR spectrum of **16** (500 MHz, $CDCl_3$)

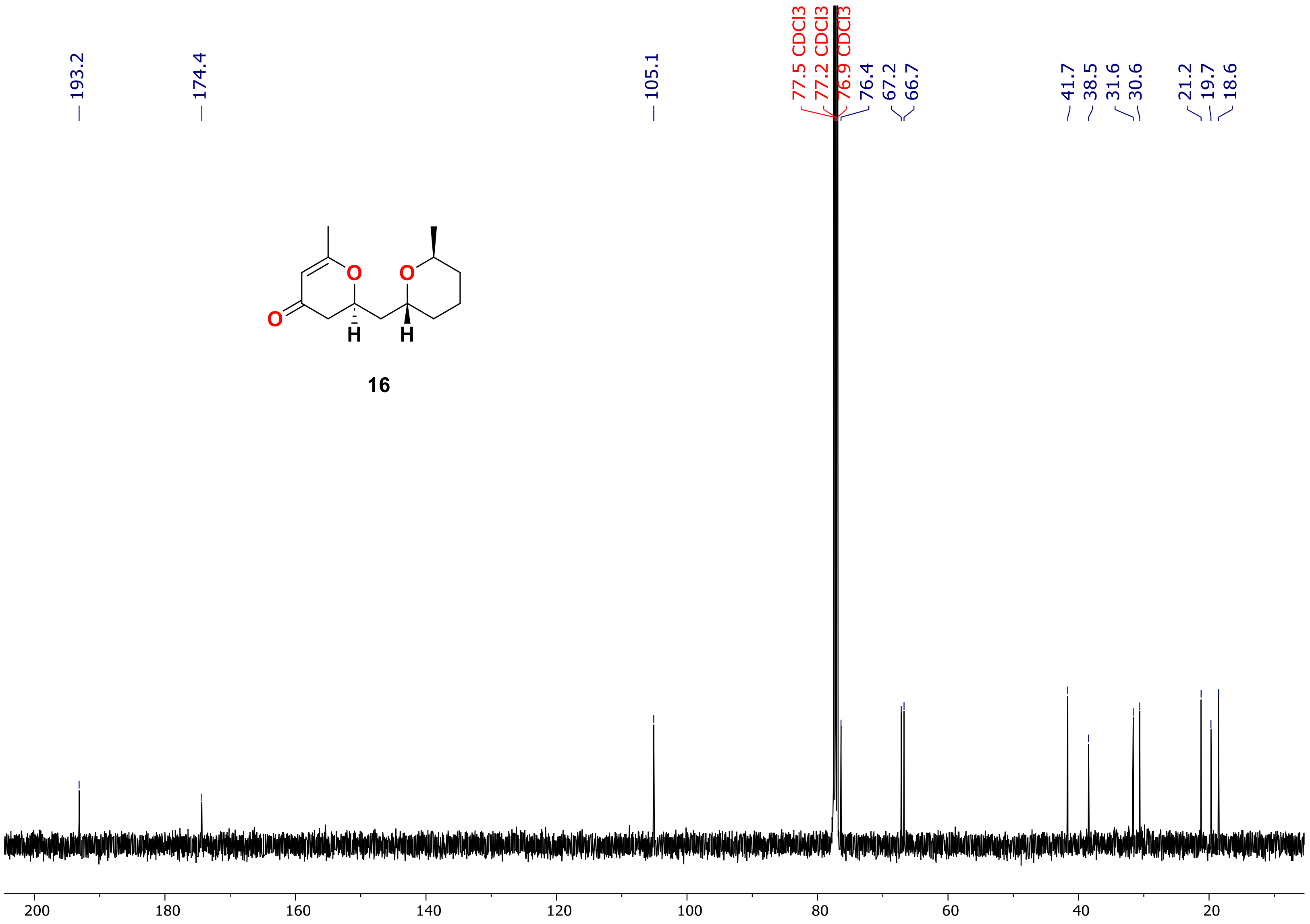

Figure S68 ^{13}C NMR spectrum of **16** (125 MHz, $CDCl_3$)

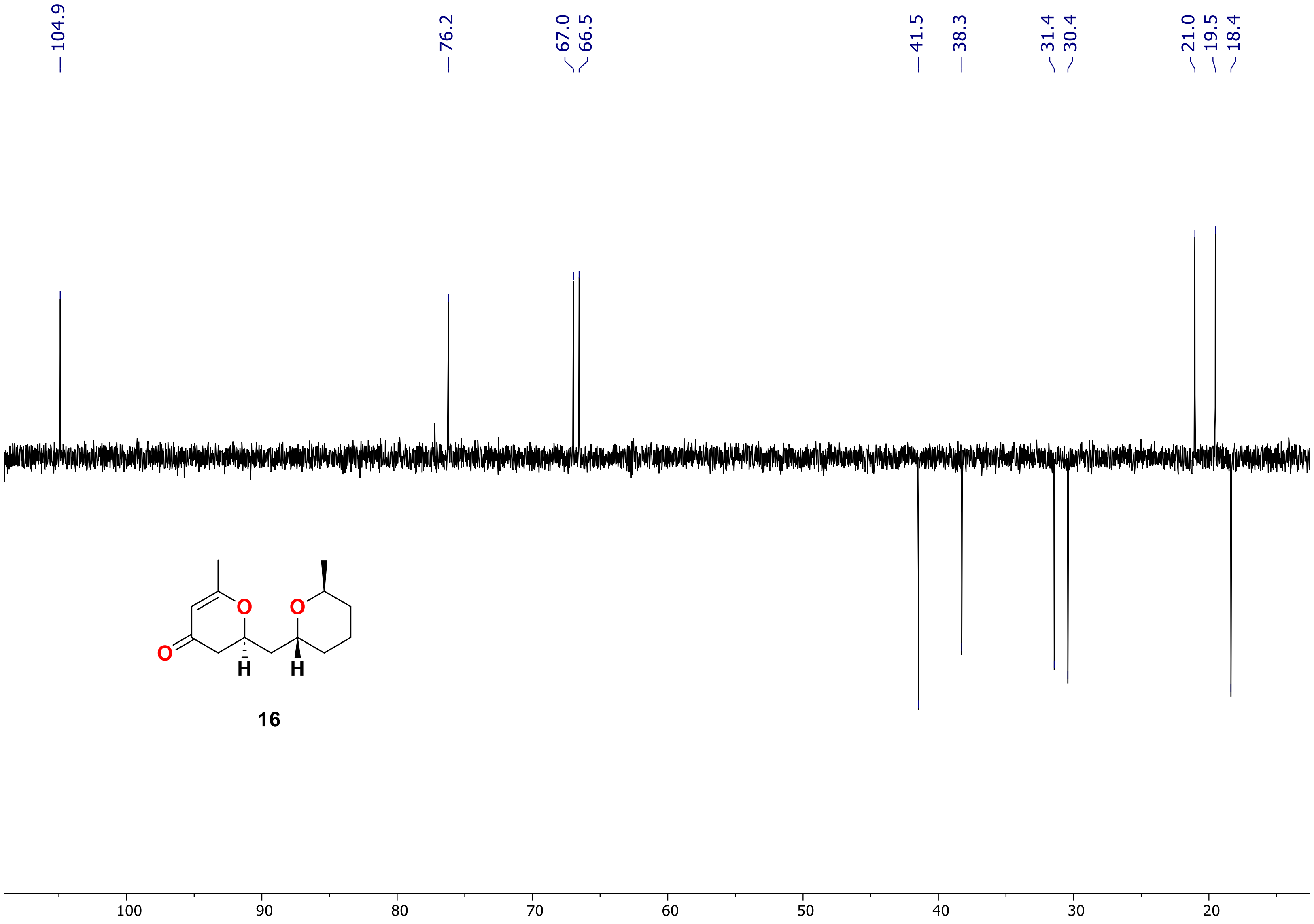

Figure S69 DEPT135 spectrum of **16**

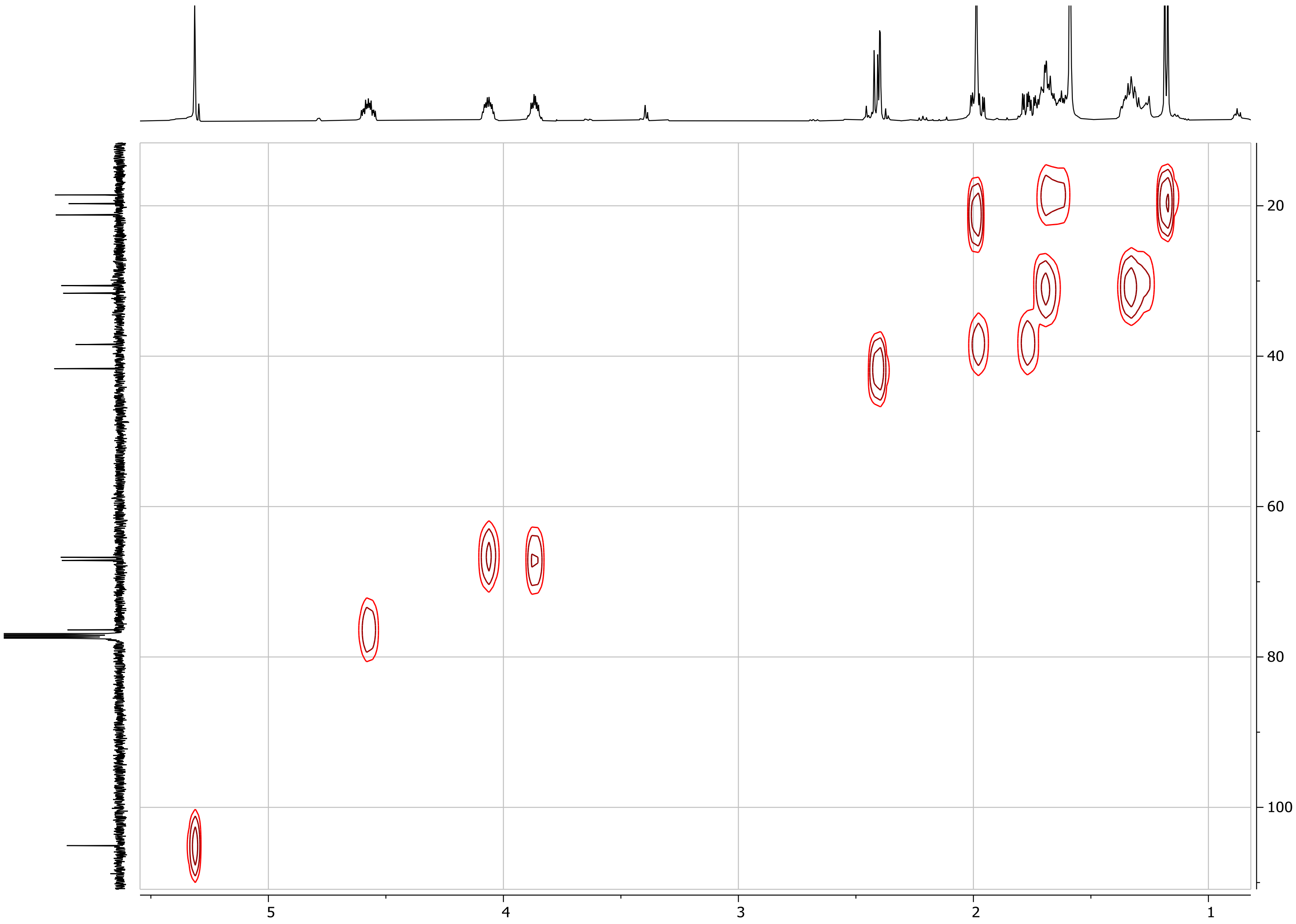

Figure S70 HSQC spectrum of **16**

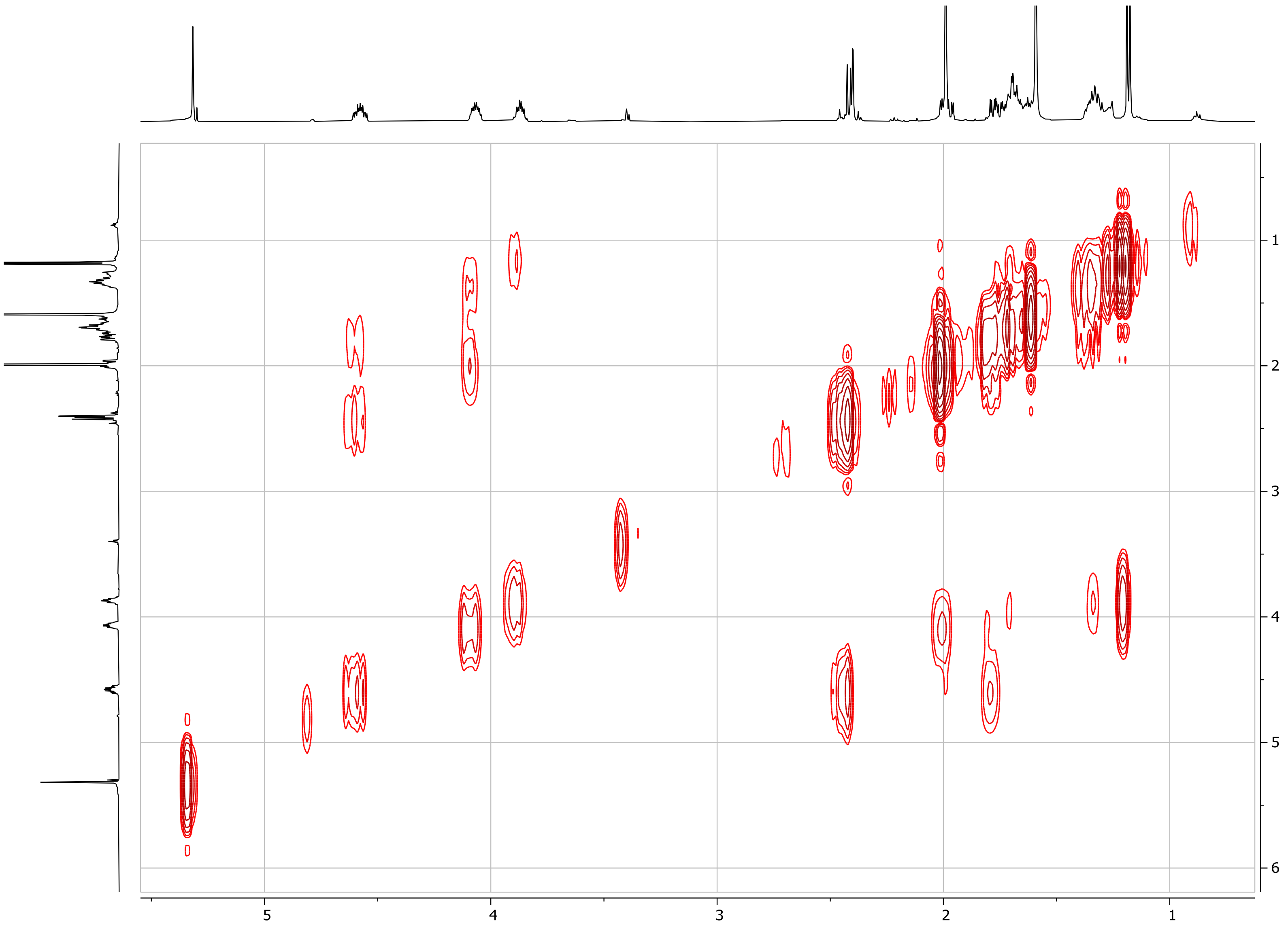

Figure S71 COSY spectrum of **16**

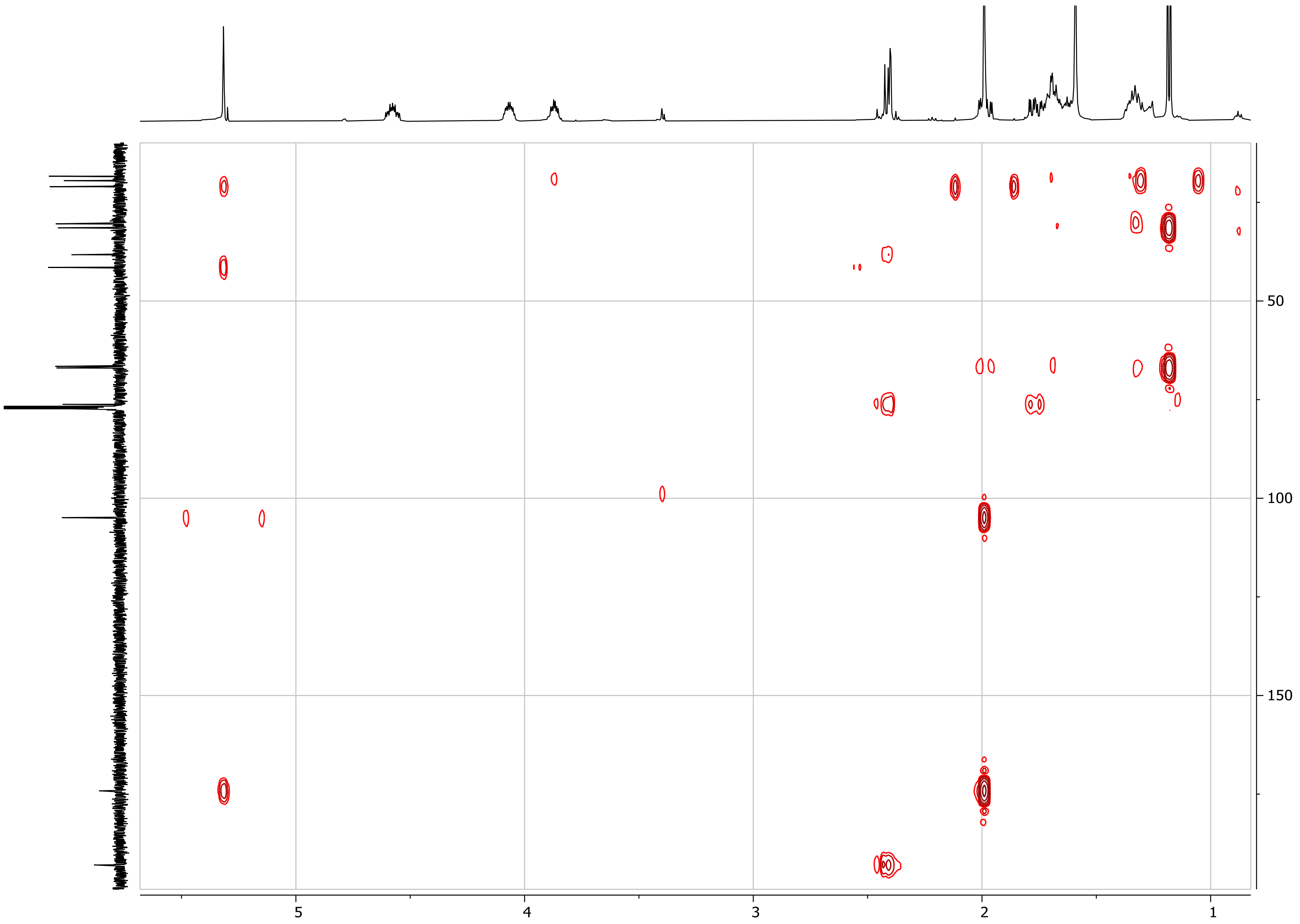

Figure S72 HMBC spectrum of **16**

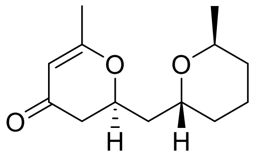

(6*S**, 2'*R**,6'*S**)-**16a**

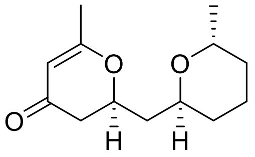

(6*S**, 2'*S**,6'*R**)-**16c**

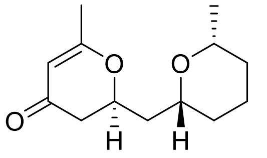

(6*S**, 2'*R**,6'*R**)-**16e**

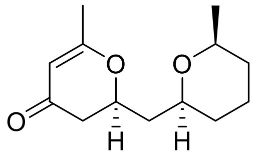

(6*S**, 2'*S**,6'*S**)-**16g**

Figure S73 Structures of **16a**, **16c**, **16e** and **16g** diastereomers used for ^{13}C NMR chemical shift calculations

Table S21 Experimental ^{13}C NMR chemical shifts of **16** and Boltzmann averaged shielding values of **16a**, **16c**, **16e**, and **16g** diastereomers

Position	Expt. δ_C of **16**	Boltzmann averaged shielding values			
		**16a**	**16c**	**16e**	**16g**
2	174.2	22.68	25.11	22.64	23.93
3	104.9	89.48	90.01	92.10	90.29
4	193.0	8.46	10.17	10.42	9.96
5	41.5	150.22	149.58	153.18	150.78
6	76.2	115.56	111.74	114.58	111.17
7	38.5	152.26	156.58	146.95	152.35
2’	66.5	124.82	117.87	117.80	113.49
3’	30.4	160.10	162.20	158.03	158.65
4’	18.4	170.44	170.12	166.19	166.07
5’	31.4	159.34	158.06	158.51	158.57
6’	67.0	124.25	125.70	118.35	118.14
1-CH_3	21.0	170.11	169.28	168.78	169.54
6’-CH_3	19.5	171.42	169.08	168.86	168.61

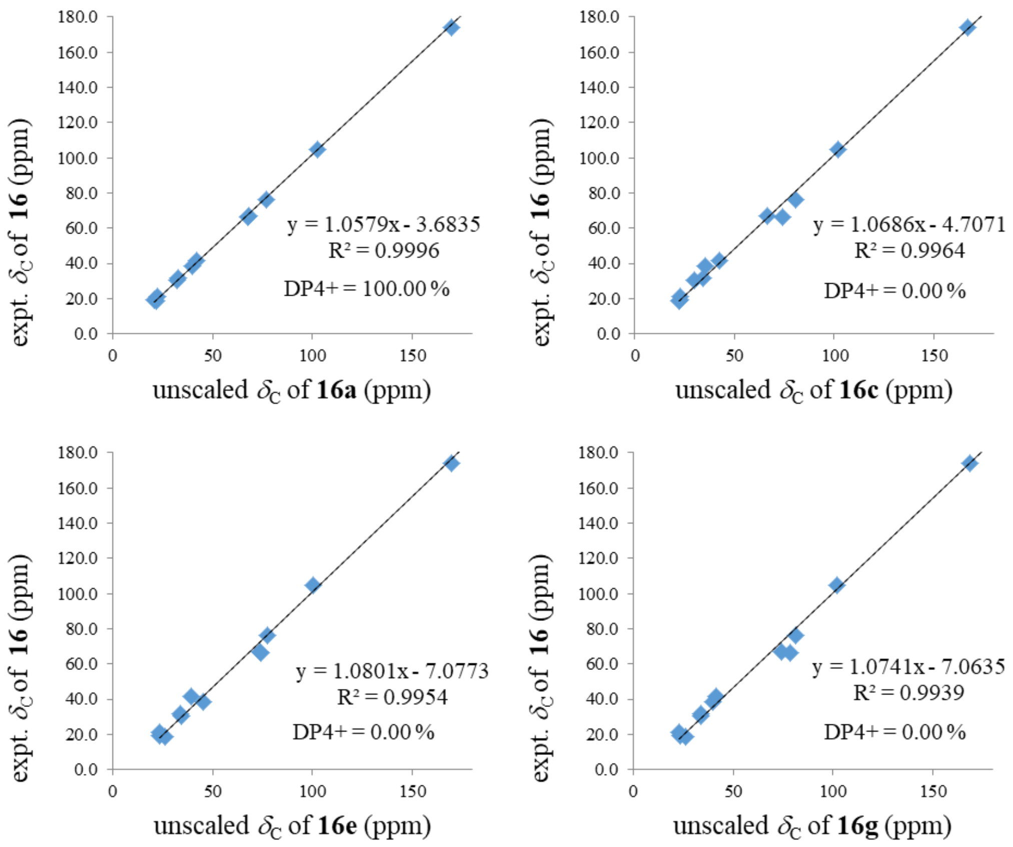

Figure S74 Linear correlations between unscaled and experimental ^{13}C NMR chemical shifts of **16a**, **16c**, **16e**, and **16g** diastereomers

Functional	Solvent?	Basis Set	Type of Data
B3LYP	PCM	6-31G(d,p)	Shielding Tensors

		DP4+	100.00%	0.00%	0.00%	0.00%	-	-	-	-	-	-	-	-
Nuclei	sp2?	Experimental	Isomer 1	Isomer 2	Isomer 3	Isomer 4	Isomer 5	Isomer 6	Isomer 7	Isomer 8	Isomer 9	Isomer 10	Isomer 11	Isomer 12
c		174.2	22.68	25.11	22.64	23.93								
c		104.9	89.48	90.01	92.10	90.29								
c		193.0	8.46	10.17	10.42	9.96								
c	x	41.5	150.22	149.58	153.18	150.78								
c	x	76.2	115.56	111.74	114.58	111.17								
c	x	38.5	152.26	156.58	146.95	152.35								
c	x	66.5	124.82	117.87	117.80	113.49								
c	x	30.4	160.10	162.20	158.03	158.65								
c	x	18.4	170.44	170.12	166.19	166.07								
c	x	31.4	159.34	158.06	158.51	158.57								
c	x	67.0	124.25	125.70	118.35	118.14								
c	x	19.5	171.42	169.08	168.86	168.61								
c	x	21.0	170.11	169.28	168.78	169.54								

Main Detailed Results

Functional	Solvent?	Basis Set	Type of Data
B3LYP	PCM	6-31G(d,p)	Shielding Tensors

	Isomer 1	Isomer 2	Isomer 3	Isomer 4	Isomer 5	Isomer 6	Isomer 7	Isomer 8	Isomer 9	Isomer 10	Isomer 11	Isomer 12	Isomer 13
sDP4+ (H data)	-	-	-	-	-	-	-	-	-	-	-	-	-
sDP4+ (C data)	100.00%	0.00%	0.00%	0.00%	-	-	-	-	-	-	-	-	-
sDP4+ (all data)	100.00%	0.00%	0.00%	0.00%	-	-	-	-	-	-	-	-	-
uDP4+ (H data)	-	-	-	-	-	-	-	-	-	-	-	-	-
uDP4+ (C data)	99.78%	0.22%	0.00%	0.00%	-	-	-	-	-	-	-	-	-
uDP4+ (all data)	99.78%	0.22%	0.00%	0.00%	-	-	-	-	-	-	-	-	-
DP4+ (H data)	-	-	-	-	-	-	-	-	-	-	-	-	-
DP4+ (C data)	100.00%	0.00%	0.00%	0.00%	-	-	-	-	-	-	-	-	-
DP4+ (all data)	100.00%	0.00%	0.00%	0.00%	-	-	-	-	-	-	-	-	-

Main Detailed Results

Figure S75 The results of DP4+ analysis of **16a**, **16c**, **16e**, and **16g** diastereomers

Table S22 The results of Energy analysis for conformers of **16aa-ab**

conformer	Energy (Hartree)[a]	% population[b]
**16aa**	733.133424	55.41
**16ab**	733.133629	44.59

[a] calculated using B3LYP/6-31G(d,p) level at 298.15 K

[b] calculated using %Boltzmann distribution = $\frac{e^{-E_i/RT}}{\sum_i e^{-E_i/RT}}$ x 100

Table S23 Cartesian coordinates for the low-energy optimized conformers of **16a**

Conformer **16aa**				
Tag	Symbol	X	Y	Z
1	C	-3.162973	-1.276455	-0.355119
2	C	-3.556914	-0.116934	0.438510
3	C	-1.853722	-1.091889	-1.126764
4	C	-0.896888	-0.180997	-0.365664
5	O	-1.548792	1.078815	-0.038572
6	C	-2.786714	0.998962	0.498570
7	C	0.378022	0.171125	-1.122974
8	C	1.388575	0.966062	-0.272951
9	C	2.627776	1.424662	-1.060223
10	C	3.611905	0.273408	-1.307500
11	C	3.939635	-0.422113	0.020352
12	C	2.655899	-0.855906	0.737447
13	O	1.764380	0.254542	0.915343
14	H	-0.636354	-0.644011	0.595714
15	H	0.866823	1.848873	0.109627
16	C	2.906543	-1.421965	2.129024
17	C	-3.190195	2.286713	1.148204
18	O	-3.835436	-2.294281	-0.456625
19	H	-4.527153	-0.134322	0.921888
20	H	-2.090883	-0.656181	-2.108669
21	H	-1.401164	-2.072604	-1.302379
22	H	0.822620	-0.760345	-1.492526
23	H	0.109845	0.764020	-2.007111
24	H	3.133255	2.201966	-0.472468
25	H	2.316749	1.889288	-2.004460
26	H	4.525510	0.652445	-1.781044
27	H	3.179945	-0.454818	-2.008344
28	H	4.581343	-1.297465	-0.142512
29	H	4.489851	0.268438	0.674877
30	H	2.161215	-1.629170	0.124208
31	H	3.390254	-0.670639	2.762789
32	H	3.554023	-2.304153	2.075993
33	H	1.962294	-1.710494	2.601594
34	H	-3.133145	3.106172	0.421665
35	H	-4.208689	2.229286	1.537989
36	H	-2.503250	2.533062	1.966521

Conformer **16ab**				
Tag	Symbol	X	Y	Z
1	C	3.5268220	-0.7745910	-0.5116090
2	C	3.5094490	0.6565200	-0.2248920
3	C	2.3097340	-1.5349710	0.0202850
4	C	1.0670560	-0.6519000	0.0099920

5	O	1.3210920	0.5927090	0.7232020
6	C	2.4695340	1.2394060	0.4236340
7	C	-0.1582090	-1.2866750	0.6566670
8	C	-1.4355000	-0.4587890	0.5086700
9	C	-2.6286330	-1.0667150	1.2545990
10	C	-3.9094560	-0.2762560	0.9500180
11	C	-4.1114720	-0.1651120	-0.5688060
12	C	-2.8560650	0.3762220	-1.2759300
13	O	-1.6998230	-0.3869570	-0.8971430
14	H	0.8276390	-0.3682640	-1.0228610
15	H	-1.2465350	0.5514890	0.9025640
16	C	-2.6386690	1.8834880	-1.0941670
17	C	2.4453100	2.6579340	0.9044670
18	O	4.4528720	-1.3457380	-1.0724850
19	H	4.3906620	1.2378350	-0.4717640
20	H	2.1534980	-2.4308760	-0.5879660
21	H	2.5320680	-1.8651870	1.0459110
22	H	-0.3232100	-2.2663100	0.1909130
23	H	0.0464010	-1.4562710	1.7214670
24	H	-2.7516580	-2.1103560	0.9322950
25	H	-2.4243410	-1.0790380	2.3330310
26	H	-3.8369190	0.7244780	1.3979990
27	H	-4.7787530	-0.7594210	1.4120350
28	H	-4.3267740	-1.1631080	-0.9723600
29	H	-4.9741880	0.4707300	-0.8047440
30	H	-2.9467360	0.1800840	-2.3505790
31	H	-1.6964710	2.1828130	-1.5647220
32	H	-2.6043650	2.1837480	-0.0419350
33	H	-3.4533930	2.4406060	-1.5709900
34	H	1.6267620	3.2095840	0.4269820
35	H	2.2632510	2.6836150	1.9856390
36	H	3.3905160	3.1623750	0.6929380

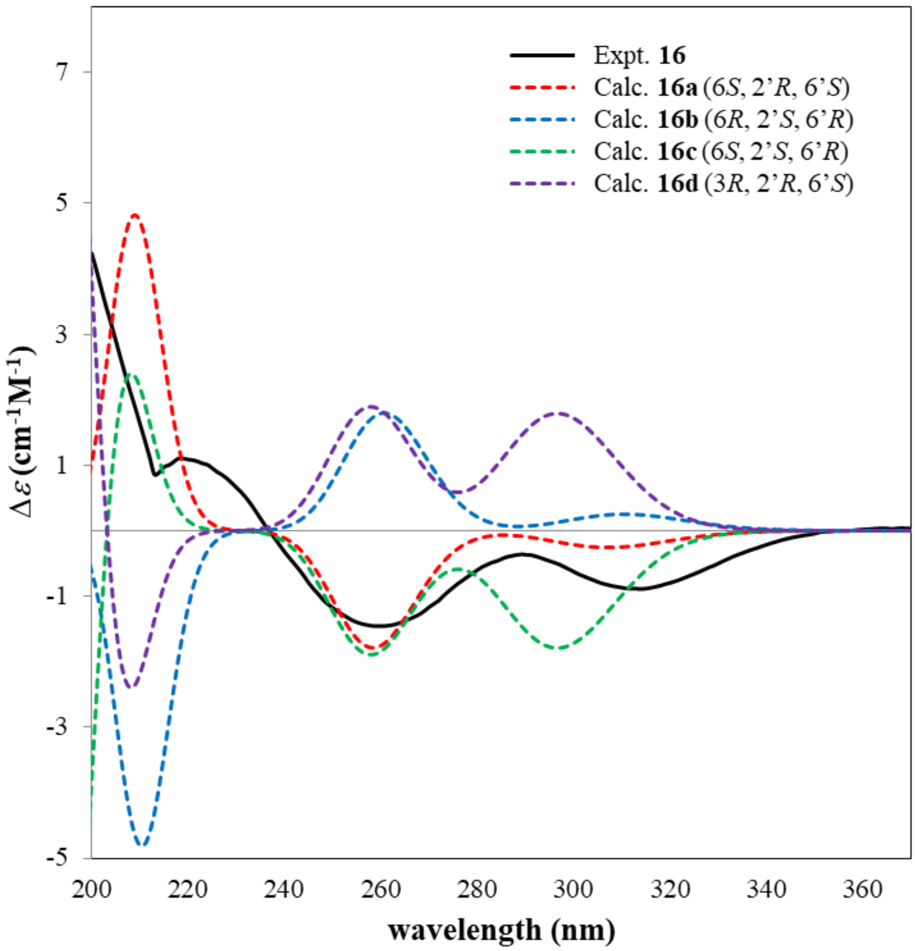

Figure S76 Comparison of experimental and calculated ECD spectra of **16**

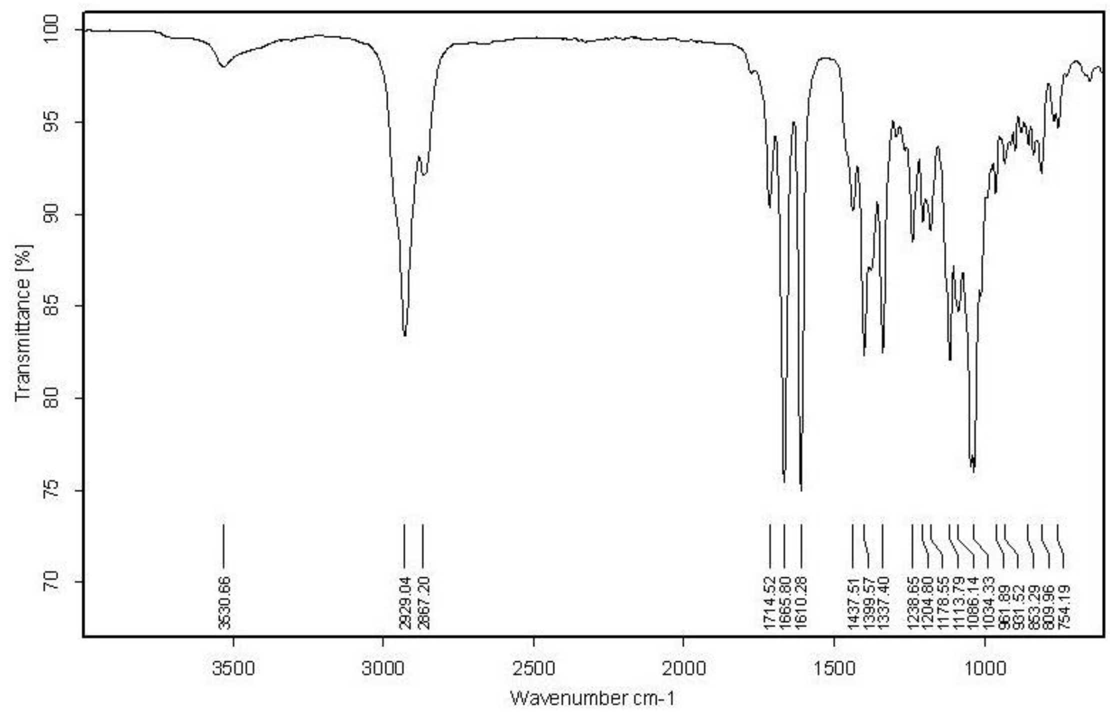

Figure S77 IR spectrum of **16**

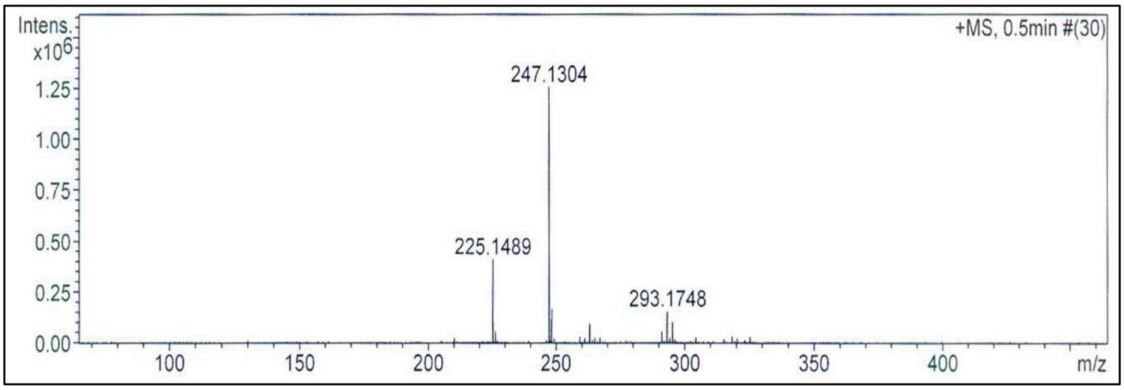

Figure S78 HRESIMS spectrum of **16**

Table S24 ^{1}H, ^{13}C, 2D NMR and NOE data of compound **17** (500 MHz, $CDCl_3$), and ^{1}H and ^{13}C NMR data of aspyran (500 MHz, $CDCl_3$)[11]

Position /DEPT	17					aspyran	
	δ_H	δ_C	COSY	HMBC	NOE	δ_H	δ_C
1 C		156.9					164.2
2 CH	6.16 (t, 2.3)	100.9		1, 3, 4, 6			102.2
3 C		156.9					160.5
4 CH	6.23 (d, 2.3)	108.7		2, 3, 6, 7		6.16 (d, 2.3)	111.2
5 C		141.9					142.1
6 CH	6.23 (d, 2.3)	108.7		1, 2, 4, 7			107.2
7 CH_2	2.87 (dd, 13.6, 7.1)<br>2.64 (dd, 13.6, 7.1)	39.2	2′	4, 5, 6, 2′, 3′		3.06 (dd, 13.7, 7.8),<br>2.82 (dd, 13.7, 4.8)	40.5
1′ O							
2′ CH	3.97-4.06 (m)	72.7	7, 3′			3.99 (m)	72.1
3′ CH_2	1.69 (1H, m)<br>1.43 (1H, m)	28.7	2′			1.42-1.73 (m)	30.5
4′ CH_2	1.69 (1H, m)	18.2	5′			1.42-1.73 (m)	17.6
5′ CH_2	1.69 (1H, m)<br>1.33 (1H, m)	31.8	4′, 6′			1.37 (m)	27.9
6′ CH	4.03 (1H, m)	67.4	5′, 6′-CH_3			4.21 (m)	69.8
6′-CH_3	1.17 (d, 6.5)	20.0	6′	5′, 6′	2′	1.13 (d, 6.6)	17.5
1-COOH						11.32 (brs)	172.5

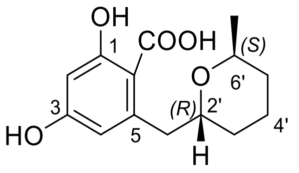

Aspyran
$[a]_D^{20.0}$ -40.0 (c = 0.1, EtOH)

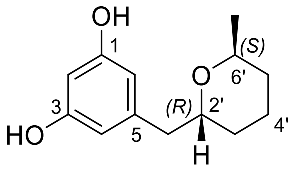

(2'*R*,6'*S*)-5-((-6-methyltetrahydro-2*H*-pyran-2-yl)methyl)benzene-1,3-diol (**17**)
$[a]_D^{27.8}$ -38.4 (c = 0.1, EtOH)

Figure S79 Structures of (2′*R*,6′*S*)-5-((-6-methyltetrahydro-2*H*-pyran-2-yl)methyl)benzene-1,3-diol (**17**) and its analog, aspyran

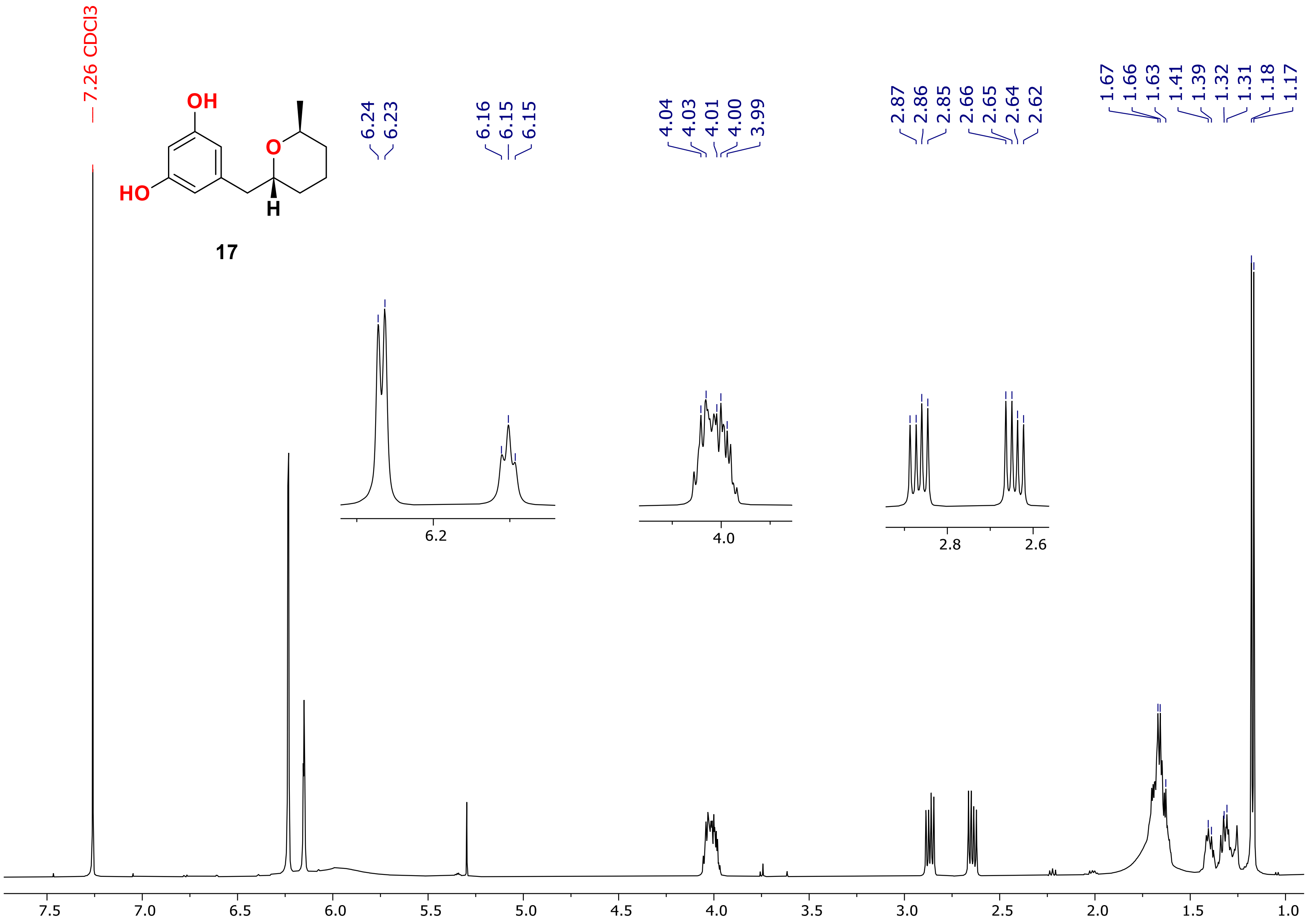

Figure S80 ^{1}H NMR spectrum of **17** (500 MHz, $CDCl_3$)

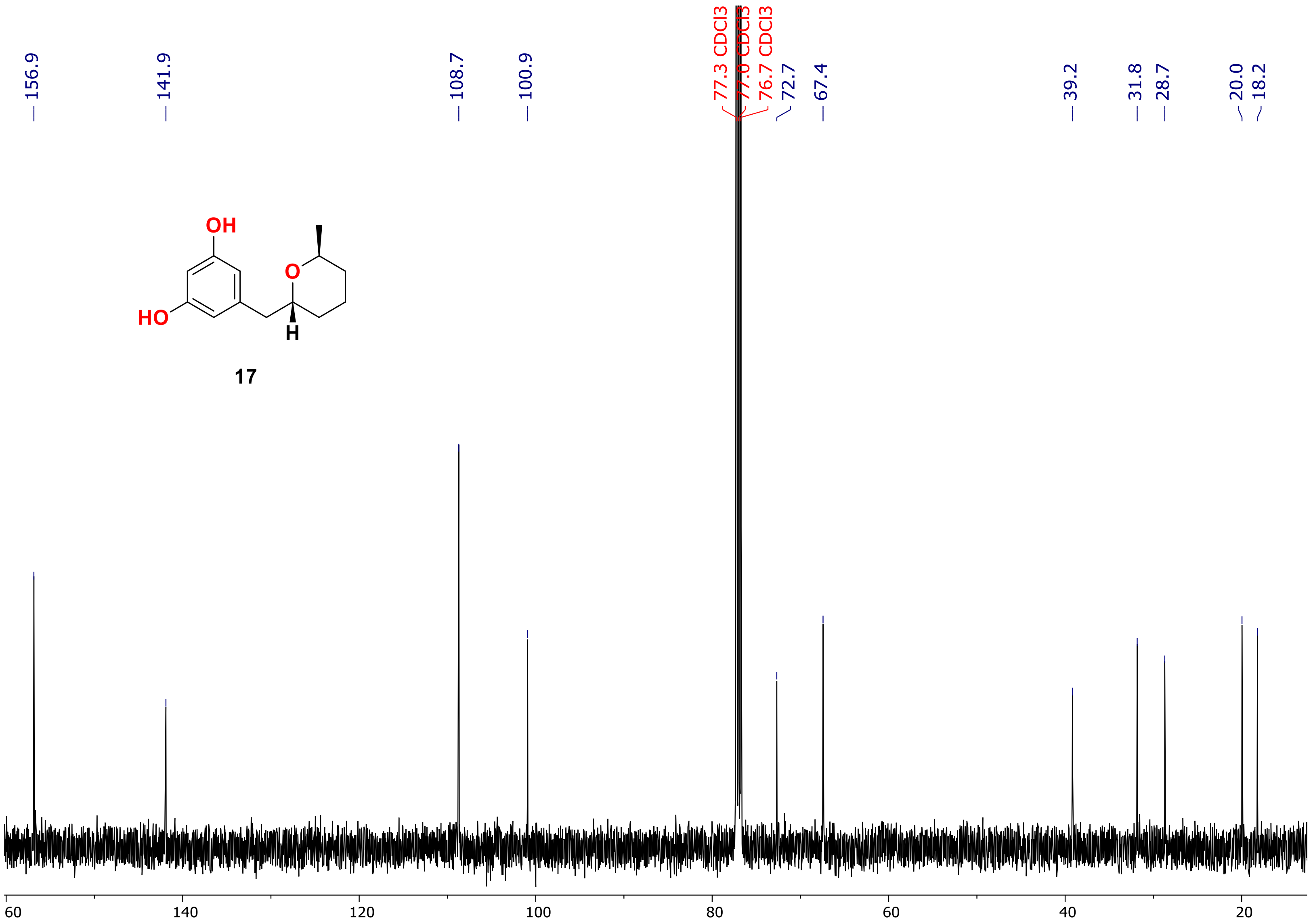

Figure S81 ^{13}C NMR spectrum of **17** (125 MHz, $CDCl_3$)

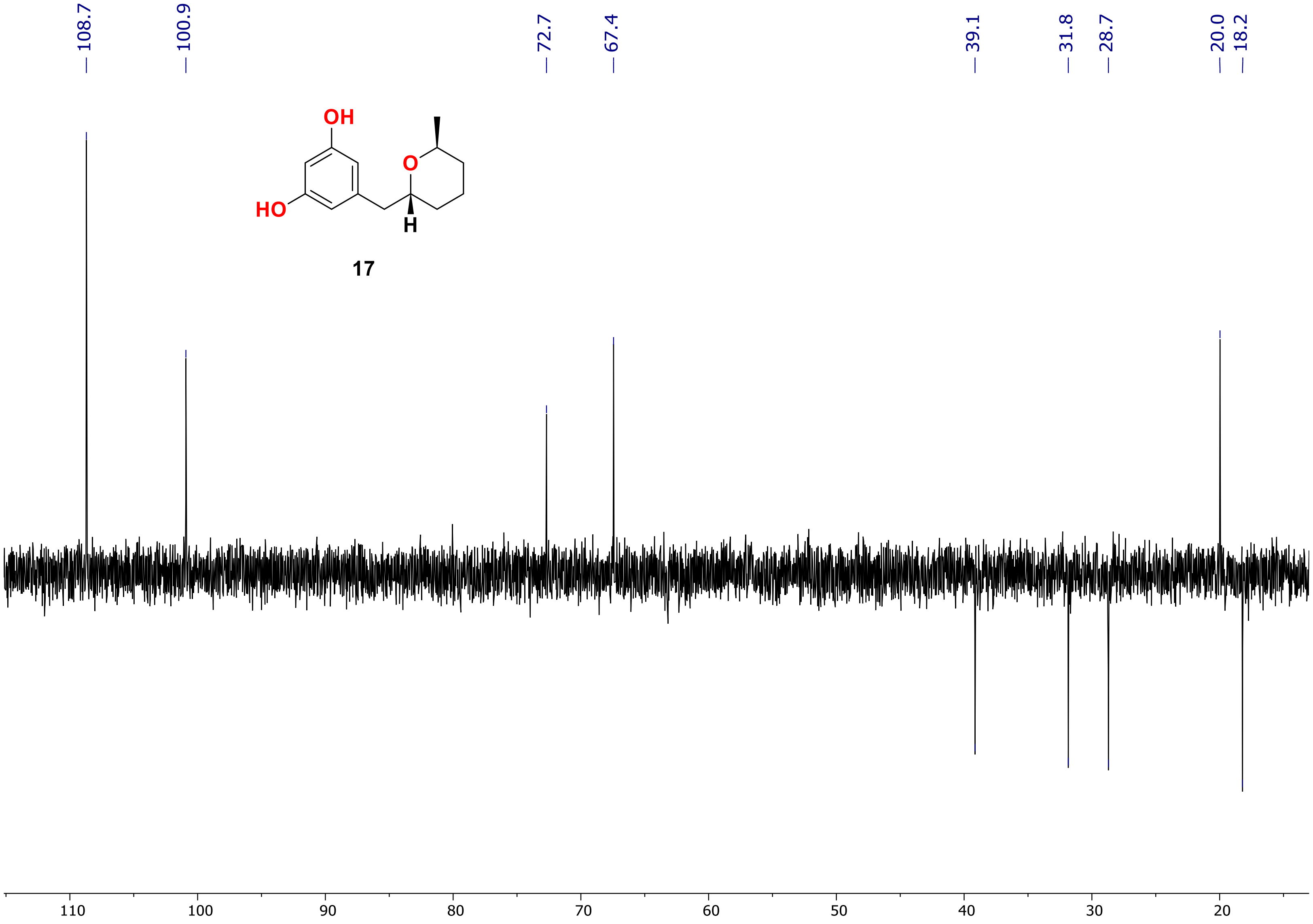

Figure S82 DEPT135 spectrum of **17**

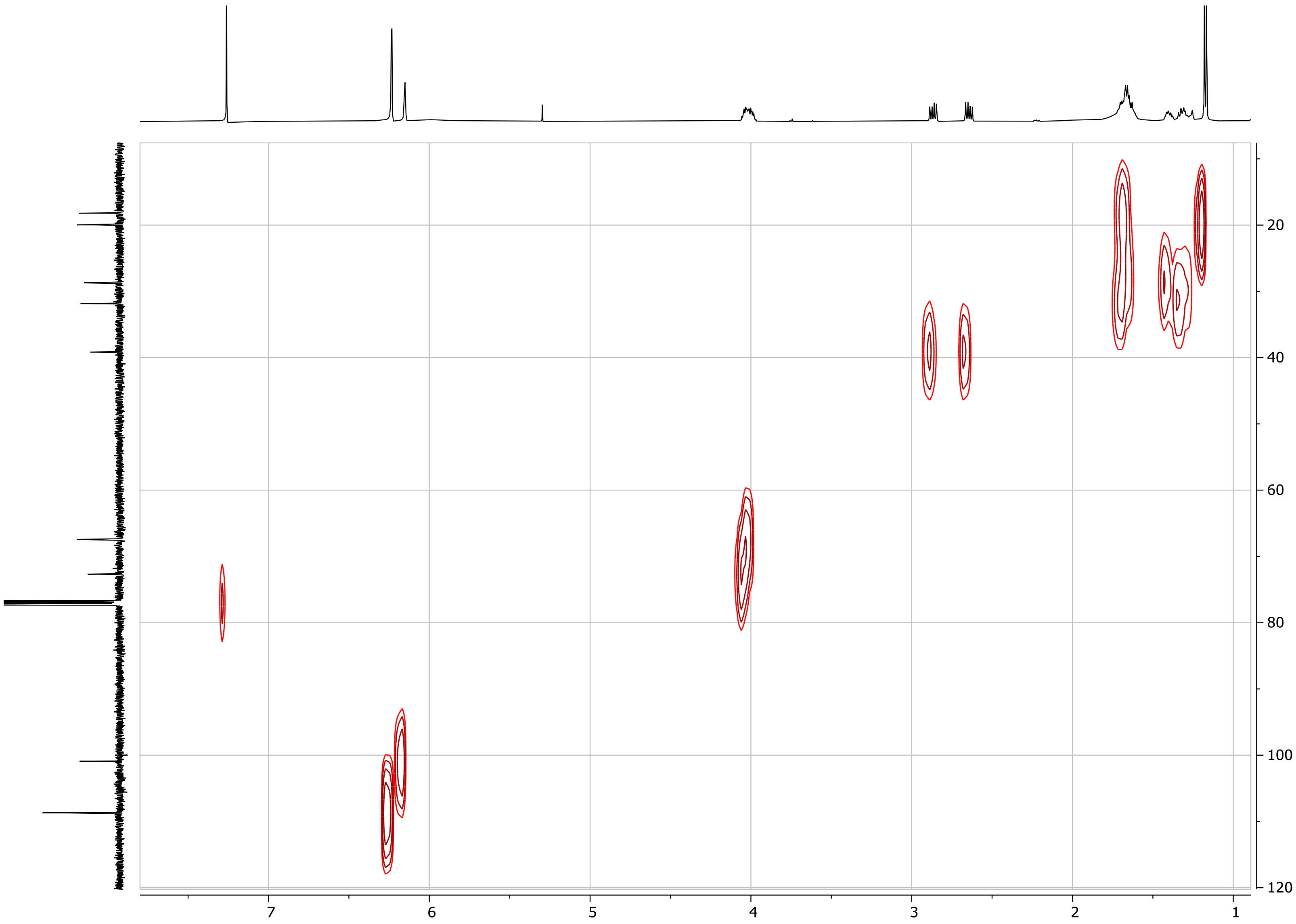

Figure S83 HSQC spectrum of **17**

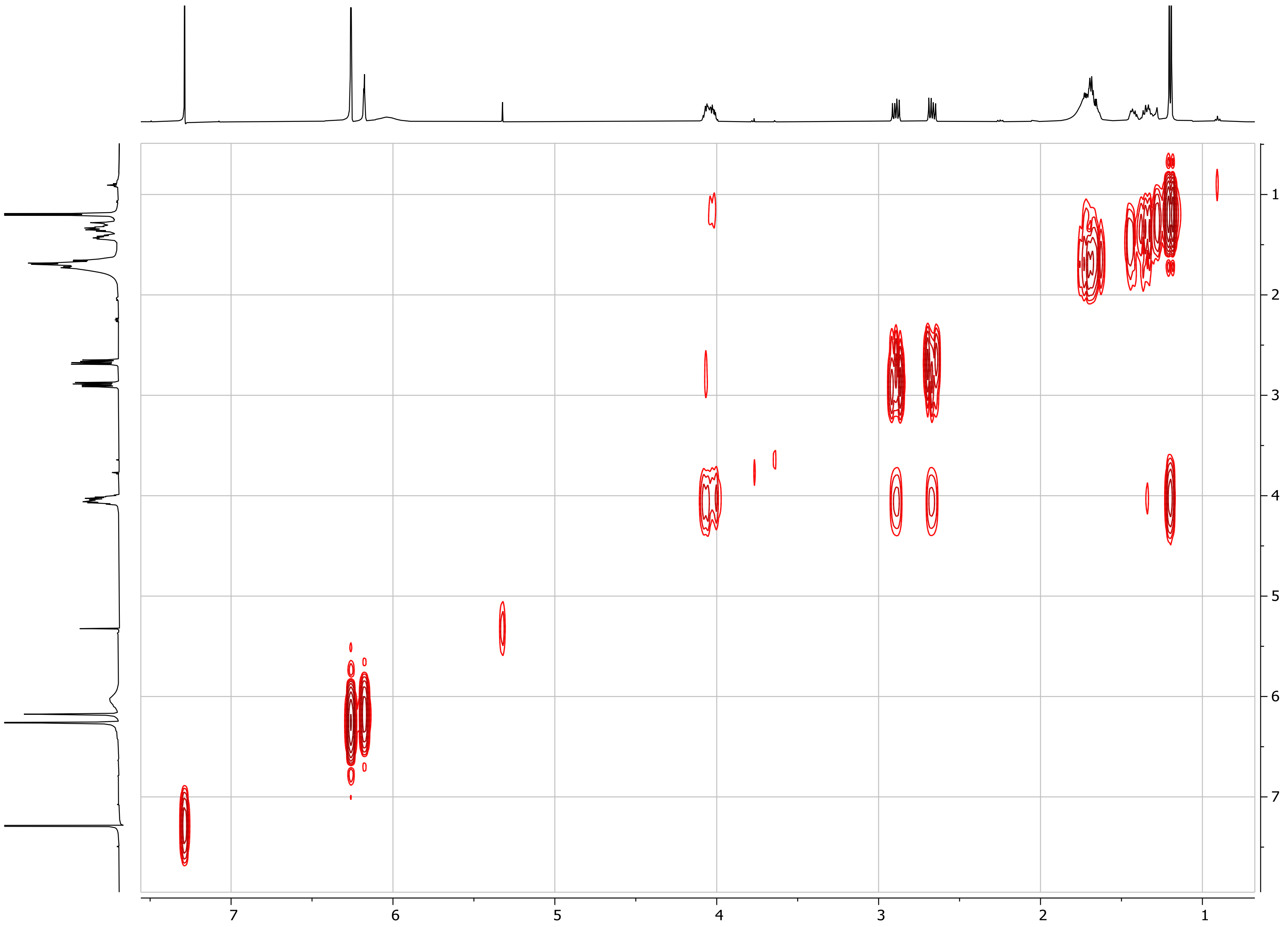

Figure S84 COSY spectrum of **17**

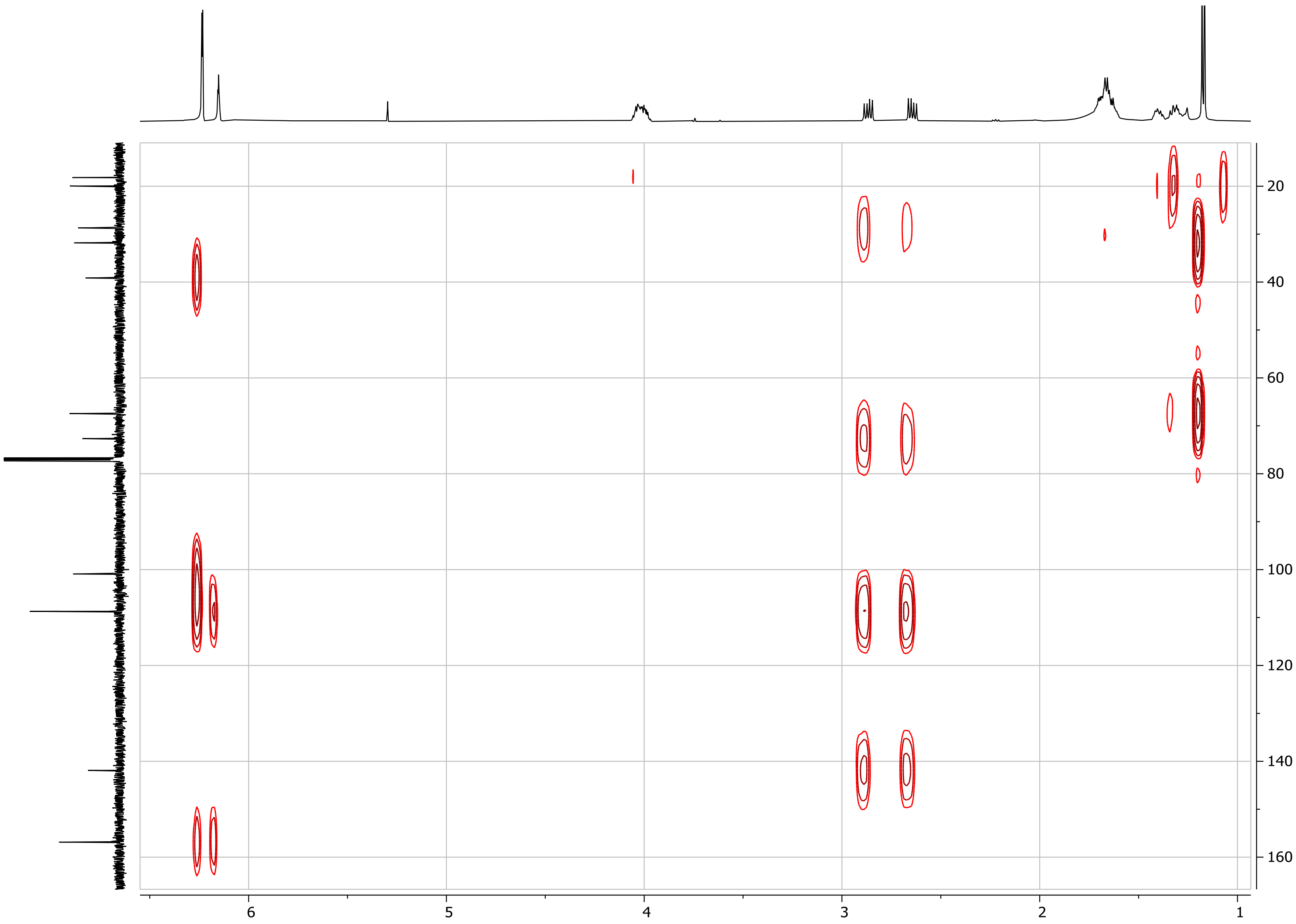

Figure S85 HMBC spectrum of **17**

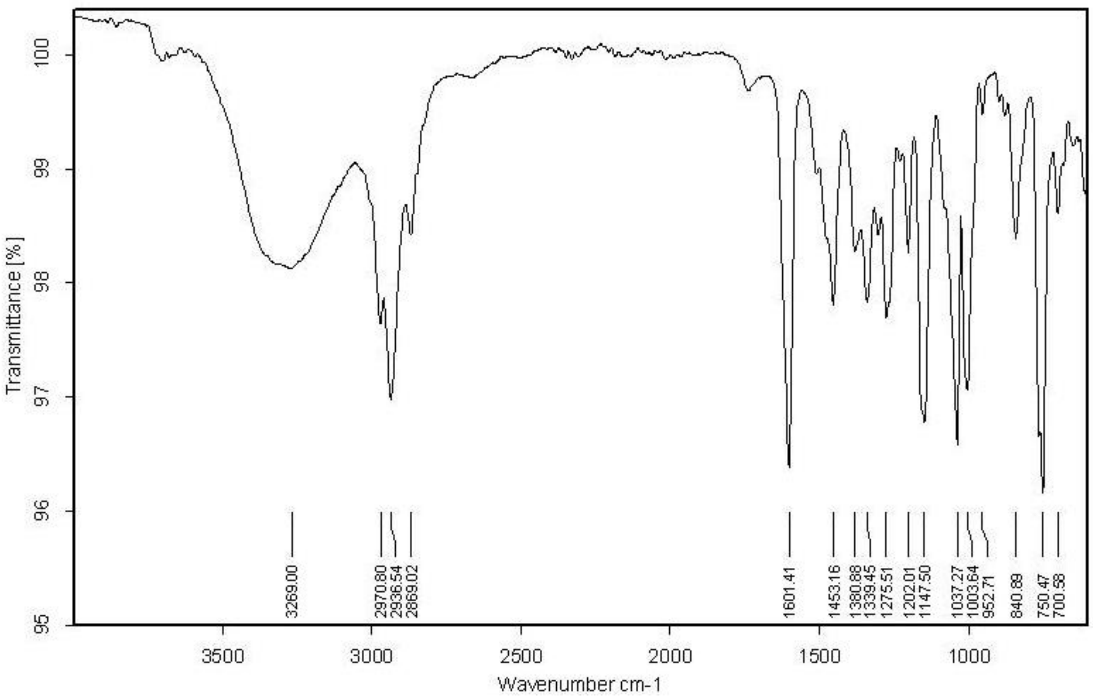

Figure S86 IR spectrum of **17**

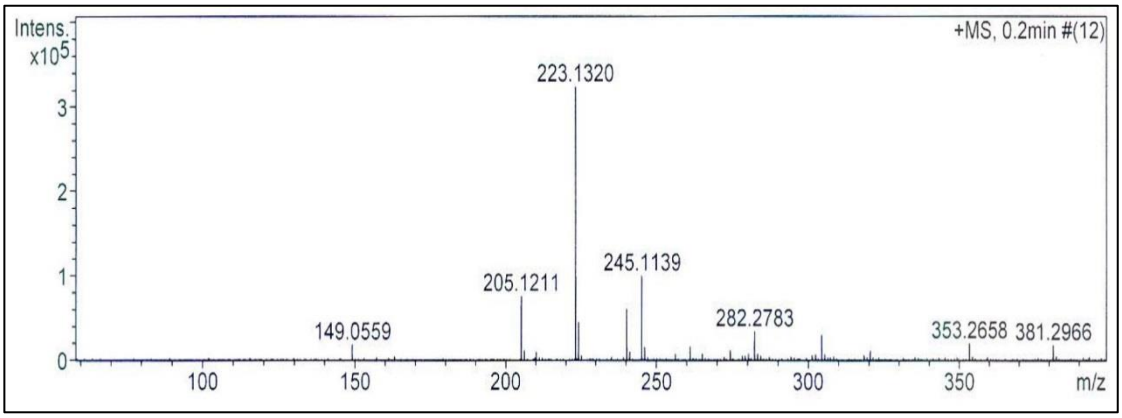

Figure S87 HRESIMS spectrum of **17**

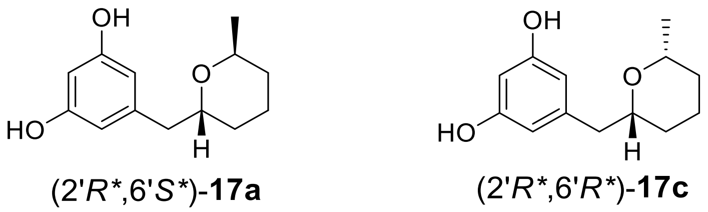

Figure S88 Structures of **17a** and **17c** diastereomers used for ^{13}C NMR chemical shift calculations

Table S 25 Experimental ^{13}C NMR chemical shifts of **17** and Boltzmann averaged shielding values of **17a** and **17c** diastereomers

Position	Expt. δ_C of **17**	Boltzmann averaged shielding values	
		**17a**	**17c**
1	156.9	40.44	40.22
2	100.9	97.94	97.86
3	156.9	40.03	40.36
4	108.7	89.16	88.68
5	141.9	53.59	54.14
6	108.7	87.96	88.22
7	39.2	149.74	147.93
2’	72.7	119.32	113.07
3’	28.7	161.48	161.21
4’	18.2	170.60	166.14
5’	31.8	160.38	158.29
6’	67.4	122.88	117.80
6’-CH_3	20.0	172.92	168.97

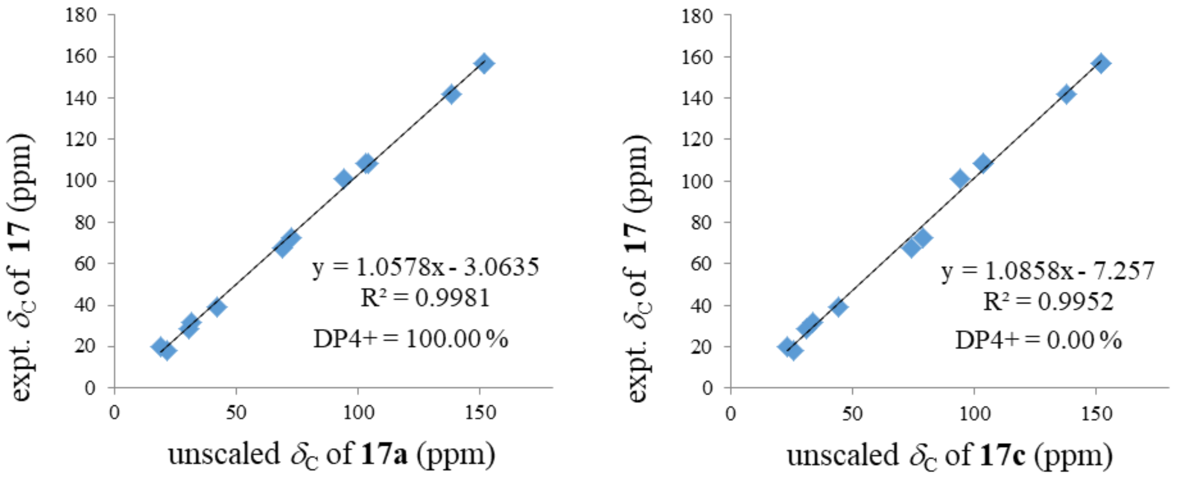

Figure S89 Linear correlations between unscaled and experimental ^{13}C NMR chemical shifts of **17a** and **17c** diastereomers

Functional	Solvent?	Basis Set	Type of Data
B3LYP	PCM	6-31G(d,p)	Shielding Tensors

Nuclei	sp2?	Experimental	Isomer 1	Isomer 2	Isomer 3	Isomer 4	Isomer 5	Isomer 6	Isomer 7	Isomer 8	Isomer 9	Isomer 10	Isomer 11
		DP4+	100.00%	0.00%	-	-	-	-	-	-	-	-	-
c		156.9	40.44	40.22									
c		100.9	97.94	97.86									
c		156.9	40.03	40.36									
c		108.7	89.16	88.68									
c		141.9	53.59	54.14									
c		108.7	87.96	88.22									
c	x	39.2	149.74	147.93									
c	x	72.7	119.32	113.07									
c	x	28.7	161.48	161.21									
c	x	18.2	170.60	166.14									
c	x	31.8	160.38	158.29									
c	x	67.4	122.88	117.80									
c	x	20	172.92	168.97									

Functional	Solvent?	Basis Set	Type of Data
B3LYP	PCM	6-31G(d,p)	Shielding Tensors

	Isomer 1	Isomer 2	Isomer 3	Isomer 4	Isomer 5	Isomer 6	Isomer 7	Isomer 8	Isomer 9	Isomer 10	Isomer 11	Isomer 12	Isomer 13
sDP4+ (H data)	-	-	-	-	-	-	-	-	-	-	-	-	-
sDP4+ (C data)	100.00%	0.00%	-	-	-	-	-	-	-	-	-	-	-
sDP4+ (all data)	100.00%	0.00%	-	-	-	-	-	-	-	-	-	-	-
uDP4+ (H data)	-	-	-	-	-	-	-	-	-	-	-	-	-
uDP4+ (C data)	100.00%	0.00%	-	-	-	-	-	-	-	-	-	-	-
uDP4+ (all data)	100.00%	0.00%	-	-	-	-	-	-	-	-	-	-	-
DP4+ (H data)	-	-	-	-	-	-	-	-	-	-	-	-	-
DP4+ (C data)	100.00%	0.00%	-	-	-	-	-	-	-	-	-	-	-
DP4+ (all data)	100.00%	0.00%	-	-	-	-	-	-	-	-	-	-	-

Figure S90 The results of DP4+ analysis of **17a** and **17c** diastereomers

Table S26 The results of energy analysis for conformers of **17aa-ae**

conformer	Energy (Hartree)[a]	% population[b]
**17aa**	731.929888	19.25
**17ab**	731.930224	13.48
**17ac**	731.930267	12.88
**17ad**	731.929269	37.07
**17ae**	731.929988	17.31

[a] calculated using B3LYP/6-31G(d,p) level at 298.15 K

[b] calculated using %Boltzmann distribution = $\frac{e^{-E_i/RT}}{\sum_i e^{-E_i/RT}}$ x 100

Table S27 Cartesian coordinates for the low-energy optimized conformers of **17a**

Conformer **17aa**				
Tag	Symbol	X	Y	Z
1	C	-2.536577	1.298157	-0.421921
2	C	-3.484697	0.450519	0.148955
3	C	-1.270377	0.821586	-0.783527
4	C	-0.938504	-0.518953	-0.571249
5	C	-1.887994	-1.375461	-0.002181
6	C	-3.149594	-0.887734	0.356138
7	C	0.433808	-1.044546	-0.932839
8	C	1.390910	-1.156622	0.281257
9	C	2.737167	-1.819198	-0.065314
10	C	3.675877	-0.861351	-0.813089
11	C	3.804960	0.454084	-0.032759
12	C	2.421009	1.044154	0.263001
13	O	1.592555	0.094476	0.942181
14	H	0.882254	-1.767300	1.036451
15	C	2.468256	2.276487	1.156635
16	O	-4.109270	-1.688808	0.912717
17	O	-2.903800	2.602390	-0.613144
18	H	-4.464494	0.823322	0.424271
19	H	-0.545641	1.498009	-1.233017
20	H	-1.647779	-2.425674	0.158554
21	H	0.339865	-2.049645	-1.364750
22	H	0.879129	-0.415708	-1.711865
23	H	3.216222	-2.119551	0.875759
24	H	2.564874	-2.735283	-0.644484
25	H	3.287230	-0.655595	-1.820354
26	H	4.659107	-1.327339	-0.951050
27	H	4.317932	0.270392	0.921792
28	H	4.405598	1.184151	-0.590657
29	H	1.951810	1.320152	-0.697454
30	H	1.455946	2.647282	1.345440
31	H	3.054376	3.074048	0.686350
32	H	2.925408	2.028861	2.120833
33	H	-3.747950	-2.583088	1.011871
34	H	-2.155223	3.081772	-1.000352

Conformer **17ab**				
Tag	Symbol	X	Y	Z
1	C	-2.605455	1.285831	-0.358062
2	C	-3.546731	0.477258	0.280778
3	C	-1.385052	0.764180	-0.799427
4	C	-1.091044	-0.589055	-0.598957
5	C	-2.030018	-1.405607	0.040477

6	C	-3.248578	-0.870000	0.476172
7	C	0.231387	-1.169355	-1.057224
8	C	1.442890	-0.749698	-0.209824
9	C	2.695828	-1.577773	-0.522372
10	C	3.908107	-1.034553	0.248232
11	C	4.070089	0.471004	-0.009953
12	C	2.760454	1.238006	0.244973
13	O	1.679686	0.634432	-0.483562
14	H	1.187284	-0.879481	0.854441
15	C	2.423730	1.426392	1.729452
16	O	-4.196057	-1.634084	1.103703
17	O	-2.934974	2.603136	-0.531204
18	H	-4.492353	0.888175	0.615324
19	H	-0.653699	1.407414	-1.280536
20	H	-1.817100	-2.462366	0.195762
21	H	0.171988	-2.264464	-1.041962
22	H	0.439385	-0.874408	-2.093748
23	H	2.888371	-1.527978	-1.603415
24	H	2.516083	-2.631038	-0.270673
25	H	4.820543	-1.568054	-0.044482
26	H	3.771601	-1.221636	1.322214
27	H	4.874008	0.890711	0.608098
28	H	4.356833	0.623293	-1.058739
29	H	2.843518	2.233691	-0.206391
30	H	3.172970	2.069589	2.205467
31	H	2.399280	0.482249	2.283040
32	H	1.443715	1.903660	1.830254
33	H	-3.865828	-2.543239	1.170237
34	H	-2.199489	3.046231	-0.981972

Conformer **17ac**

Tag	Symbol	X	Y	Z
1	C	3.011677	1.185527	-0.295362
2	C	3.785416	0.095362	0.099153
3	C	1.652476	1.030122	-0.598566
4	C	1.051545	-0.229744	-0.508272
5	C	1.826033	-1.327974	-0.115176
6	C	3.182347	-1.160179	0.185478
7	C	-0.421043	-0.411969	-0.807619
8	C	-1.291965	-0.575958	0.466462
9	C	-1.424563	0.696466	1.319499
10	C	-2.394668	1.710098	0.695174
11	C	-3.728669	1.025919	0.367016
12	C	-3.498715	-0.223237	-0.491324
13	O	-2.577979	-1.117524	0.146108
14	H	-0.825384	-1.358428	1.075655

15	C	-4.768992	-1.026995	-0.736204
16	O	3.977675	-2.204026	0.569581
17	O	3.639496	2.398141	-0.373547
18	H	4.837365	0.219043	0.329105
19	H	1.065774	1.891512	-0.913632
20	H	1.374667	-2.316997	-0.052276
21	H	-0.778359	0.428806	-1.412176
22	H	-0.571325	-1.319242	-1.405049
23	H	-0.435030	1.140197	1.478208
24	H	-1.807940	0.402599	2.305283
25	H	-1.965472	2.135540	-0.223376
26	H	-2.550389	2.552455	1.380111
27	H	-4.227600	0.721795	1.298010
28	H	-4.405344	1.713774	-0.156539
29	H	-3.086003	0.093065	-1.464871
30	H	-4.550883	-1.910528	-1.344441
31	H	-5.516375	-0.418886	-1.258023
32	H	-5.195032	-1.363791	0.215323
33	H	3.442800	-3.012654	0.589660
34	H	2.995707	3.064381	-0.659644

Conformer **17ad**

Tag	Symbol	X	Y	Z
1	C	3.2546870	-0.7560290	0.1407470
2	C	3.4006290	0.5971210	0.4391140
3	C	2.0918720	-1.2329390	-0.4779970
4	C	1.0562060	-0.3518690	-0.8075380
5	C	1.1982770	1.0095570	-0.5124630
6	C	2.3633170	1.4708340	0.1088180
7	C	-0.1917380	-0.8729280	-1.4936800
8	C	-1.5036540	-0.7762220	-0.6933370
9	C	-1.4386900	-1.3916130	0.7088220
10	C	-2.7567710	-1.1598450	1.4609840
11	C	-3.1046370	0.3356780	1.4537040
12	C	-3.0931970	0.9166810	0.0294700
13	O	-1.8571500	0.6106240	-0.6357770
14	H	-2.2691160	-1.3172020	-1.2754630
15	C	-4.3103700	0.5188570	-0.8154980
16	O	2.5433240	2.7917770	0.4189110
17	O	4.2913730	-1.5850190	0.4768030
18	H	4.3022880	0.9641680	0.9158050
19	H	1.9980970	-2.2932850	-0.7077150
20	H	0.3921540	1.6970250	-0.7517790
21	H	-0.3535580	-0.3299410	-2.4334910
22	H	-0.0433250	-1.9267200	-1.7595500
23	H	-0.6103800	-0.9309350	1.2606190

24	H	-1.2185360	-2.4641590	0.6267390
25	H	-3.5617060	-1.7391450	0.9876530
26	H	-2.6791970	-1.5242140	2.4924440
27	H	-2.3585960	0.8762870	2.0505880
28	H	-4.0822100	0.5157610	1.9188810
29	H	-3.0801210	2.0109070	0.0974870
30	H	-5.2256880	0.9288700	-0.3730090
31	H	-4.4380960	-0.5658590	-0.8923750
32	H	-4.2081490	0.9217260	-1.8284470
33	H	1.7566370	3.2828540	0.1352480
34	H	4.0635570	-2.4888950	0.2100020

Conformer **17ae**

Tag	Symbol	X	Y	Z
1	C	3.1892980	-1.0524650	0.0095350
2	C	3.6979970	0.1627300	-0.4459980
3	C	1.9232260	-1.1237120	0.6054380
4	C	1.1490770	0.0316400	0.7505260
5	C	1.6580480	1.2552700	0.2984460
6	C	2.9236070	1.3137730	-0.2956220
7	C	-0.2298040	-0.0319150	1.3719130
8	C	-1.3785420	0.0430050	0.3514740
9	C	-1.4672560	-1.1727470	-0.5775450
10	C	-2.7257510	-1.0925520	-1.4530200
11	C	-3.9664430	-0.8912790	-0.5708190
12	C	-3.7937100	0.2952750	0.3937910
13	O	-2.5693200	0.1749790	1.1334540
14	H	-1.2367280	0.9470420	-0.2641850
15	C	-3.9265360	1.6675000	-0.2795470
16	O	3.4621720	2.4865530	-0.7489450
17	O	3.9803450	-2.1567600	-0.1504980
18	H	4.6800670	0.2142600	-0.9017480
19	H	1.5435200	-2.0798790	0.9610900
20	H	1.0715220	2.1650770	0.4173800
21	H	-0.3465770	-0.9538560	1.9537410
22	H	-0.3669420	0.8021490	2.0704840
23	H	-1.5077470	-2.0804310	0.0419620
24	H	-0.5627120	-1.2368340	-1.1931580
25	H	-2.6261870	-0.2620070	-2.1655920
26	H	-2.8333990	-2.0031460	-2.0548040
27	H	-4.1276890	-1.7979120	0.0271670
28	H	-4.8661210	-0.7448920	-1.1820900
29	H	-4.5650920	0.2305140	1.1701870
30	H	-4.9424450	1.7986520	-0.6699950
31	H	-3.2320770	1.7992890	-1.1158740
32	H	-3.7343140	2.4601090	0.4510280

33	H	2.8328760	3.2043100	-0.5783970
34	H	3.5151970	-2.9277640	0.2092950

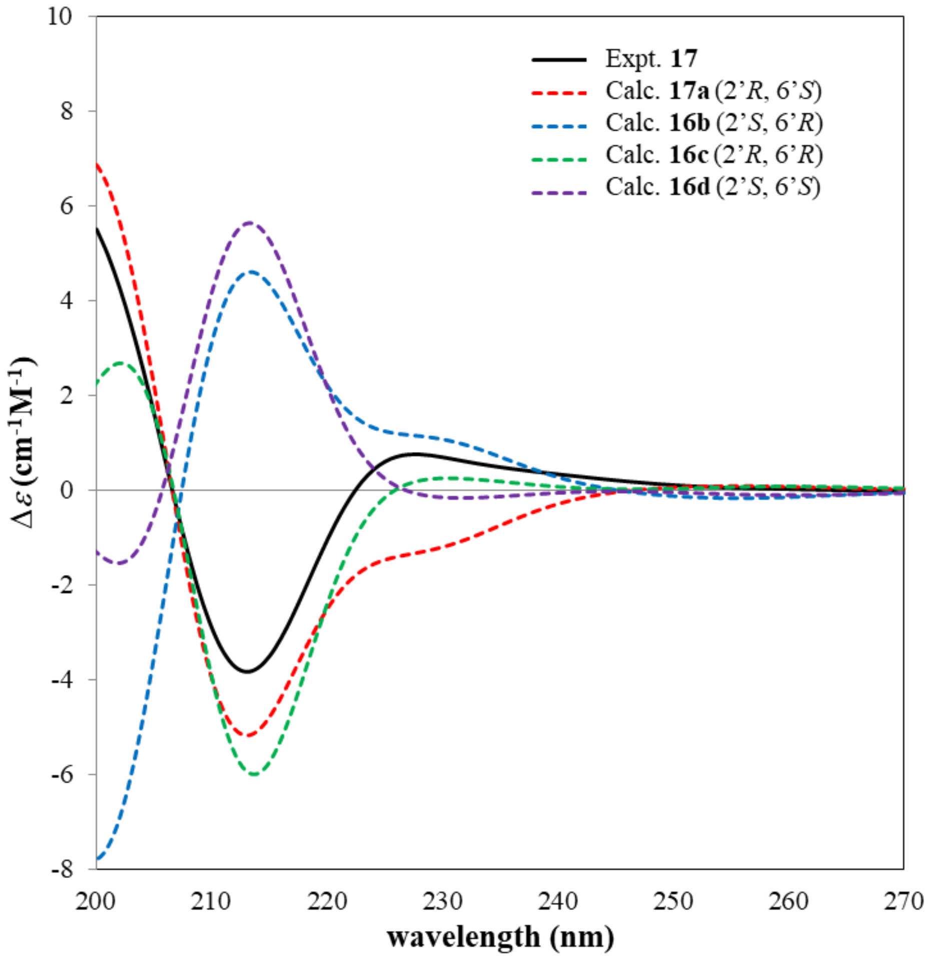

Figure S91 Comparison of experimental and calculated ECD spectra of **17**

Table S28 ^{1}H and ^{13}C NMR data of compound **18** (500 MHz, CD_3OD) and asperentin (500 MHz, CD_3OD), isocladosporin (400 MHz, $CDCl_3$) and 3-*epi*-isocladosporin (400 MHz, CD_3OD)

Position /DEPT	18		Asperentin[11]		Isocladosporin[12]		3-*epi*-isocladosporin[12]	
	δ_H	δ_C	δ_H	δ_C	δ_H	δ_C	δ_H	δ_C
1 C		171.6		171.4		171.7		170.3
2 O								
3 CH	4.56 (dddd, 9.4, 6.9, 7.0, 3.5)	78.0	4.56 (m)	77.9	4.66-4.73 (m)	78.0	4.78-4.83 (m)	76.0
4 CH_2	2.81 (m)	34.5	2.81 (m)	34.5	2.86-2.98 (m)	34.5	2.71-2.84 (m)	33.6
4a C		143.6		143.6		143.6		141.9
5 CH	6.12 (d, 2.0)	108.0	6.12 (d, 2.3)	108.0	6.22 (s)	108.1	6.34 (d, 2.0)	106.8
6 C		166.4		166.4		166.4		164.2
7 CH	6.10 (d, 2.5)	102.3	6.10 (d, 2.3)	102.3	6.20 (s)	102.3	6.17 (s)	101.9
8 C		165.7		165.5		165.8		163.3
8a C		101.8		101.6		101.7		101.3
9 CH_2	2.02 (ddd, 14.5, 10.5, 3.5), 1.56-1.70 (m)	39.4	2.02 (m), 1.58-1.63 (m)	39.3	2.00-2.07 (m), 1.56-1.66 (m)	42.2	1.71-1.92 (m), 1.50-1.60 (m)	41.7
1′ O								
2′ CH	4.03 (ddd, 13.2, 6.0, 3.5)	68.5	4.05 (m)	68.5	3.57-3.63 (m)	75.5	3.66-3.72 (m)	74.1
3′ CH_2	1.56-1.70 (m)	32.8	1.58-1.63 (m)	32.8	1.77-1.85 (m)	33.8	1.71-1.92 (m)	33.1
4′ CH_2	1.56-1.70 (m)	19.4	1.58-1.63 (m)	19.3	1.56-1.66 (m)	24.7	1.50-1.60 (m)	23.4
5′ CH_2	1.26 (m)	31.5	1.21 (m)	31.5	1.28-1.53 (m)	32.5	1.20-1.27 (m)	31.6
6′ CH	3.82 (ddt, 9.9, 6.5, 3.5)	68.4	3.81 (m)	68.4	3.44-3.51 (m)	75.2	3.45-3.49 (m)	73.2
6′-CH_3	1.08 (d, 6.5)	20.1	1.08 (d, 6.4)	20.1	1.13 (d, 6.0)	22.6	1.65 (d, 6.4)	22.0
8-OH							11.07 (d, 2.0)	

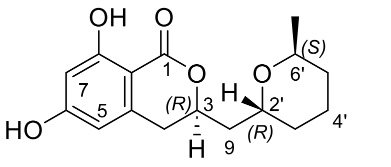

Asperentin (18)
m.p. = 183-184 °C (lit. 184-186 °C)
$[a]_D^{23.7}$ -50.2 (*c* = 1.0, EtOH)
(lit. $[a]_D^{20.0}$ -70.0 (*c* = 1.0, EtOH))

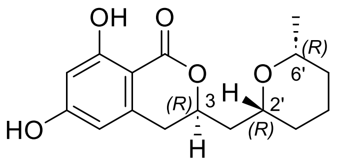

Isocladosporin
m.p. = 158-159 °C
$[a]_D^{20}$ -5.0 (*c* = 1.0, EtOH)

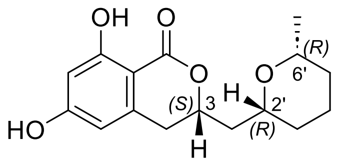

3-*epi*-isocladosporin
m.p. = 196-202 °C
$[a]_D^{20}$ -46.0 (*c* = 1.0, EtOH)

Figure S92 Structures of asperentin (**18**) and its diastereomers, isocladosporin and 3-*epi*-isocladosporin

Table S29 ^{1}H and ^{13}C NMR data of compound **19** (500 MHz, DMSO-d_6) and asperentin-8-*O*-methylether (DMSO-d_6)

Position /DEPT	19 δ_H	19 δ_C	Asperentin-8-*O*-methylether[13] δ_H	Asperentin-8-*O*-methylether[13] δ_C
1 C		161.1		161.2
2 O				
3 CH	4.35 (dquintet, 5.9, 3.0)	73.9	4.35 (m)	73.8
4 CH_2	2.85 (dd, 16.5, 3.0),<br>2.75 (dd, 16.2, 11.0)	34.4	2.79 (m)	34.4
4a C		144.0		143.9
5 CH	6.26 (d, 2.0)	106.3	6.25 (d, 1.3)	106.5
6 C		162.9		163.6
7 CH	6.37 (d, 2.0)	98.5	6.36 (d, 1.3)	98.6
8 C		162.8		162.8
8a C		104.8		104.4
9 CH_2	2.00 (ddd, 14.2, 10.2, 4.0),<br>1.60 (dd, 9.0, 3.5)	37.5	2.00 (ddd, 14.3, 10.3, 3.9),<br>1.60 (m)	37.5
1′ O				
2′ CH	3.94 (dd, 9.9, 4.8)	66.2	3.94 (m)	66.3
3′ CH_2	1.28 (dd, 13.5, 7.0),<br>1.63 (dd, 9.0, 3.5)	29.9	1.28 (m),<br>1.60 (m)	29.9
4′ CH_2	1.58 (dd, 12.5, 3.5)	18.0	1.60 (m)	18.0
5′ CH_2	1.21 (ddd, 13.1, 7.0, 1.5),<br>1.60 (dd, 9.0, 3.5)	31.4	1.21 (m),<br>1.60 (m)	31.4
6′ CH	3.80 (dt, 6.5, 3.0)	65.8	3.80 (m)	65.8
8- OCH_3	3.75 (s)	55.6	3.74 (s)	55.5
6′-CH_3	1.08 (d, 6.5)	19.8	1.08 (d, 6.3)	19.8

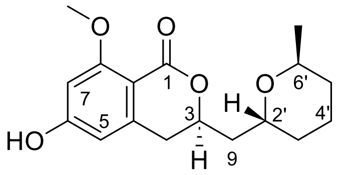

Figure S93 Structure of asperentin-8-*O*-methylether (**19**)

Table S30 ^{1}H, ^{13}C and NOE NMR data of compound **20** (500 MHz, $CDCl_3$) and asperentin-6-*O*-methylether (500 MHz, $CDCl_3$)

Position /DEPT	**20**			**Asperentin-6-*O*-methylether**[11]	
	δ_H	δ_C	**NOE**	δ_H	δ_C
1 C		169.7			169.7
2 O					
3 CH	4.73 (ddt, 8.5, 6.8, 3.5)	76.4	2′	4.72 (m)	76.4
4 CH_2	2.88 (d, 7.0)	33.8		2.88 (d, 6.9)	33.8
4a C		141.1			141.1
5 CH	6.24 (d, 1.5)	106.2		6.24 (d, 2.3)	106.1
6 C		165.8			165.7
7 CH	6.36 (d, 2.0)	99.4		6.31 (d, 2.3)	99.4
8 C		164.5			164.5
8a C		101.9			101.8
9 CH_2	1.92 (dt, 13.2, 3.2), 1.77 (dt, 13.4, 2.0)	39.5		1.83-1.96 (m)	39.5
1′ O					
2′ CH	4.04 (ddd, 13.6, 7.0, 3.5)	66.3	3, 6′-CH_3	4.08 (m)	66.2
3′ CH_2	1.65 (m)	31.0		1.34 (m)	30.9
4′ CH_2	1.63 (m)	18.3		1.66-1.73 (m)	18.3
5′ CH_2	1.30 (m)	31.0		1.66-1.73 (m)	30.9
6′ CH	3.89 (ddt, 7.9, 6.5, 3.5)	67.6	6′-CH_3	3.95 (m)	67.5
6-OCH_3	3.75 (s)	55.5		3.82 (s)	55.5
8-OH	11.22 (s)			11.22 (s)	
6′-CH_3	1.15 (d, 6.4)	19.0	2′, 6′	1.21 (d, 6.4)	19.0

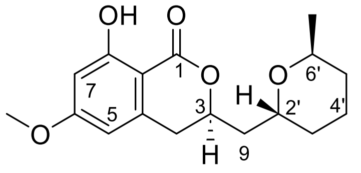

Figure S94 Structure of asperentin-6-*O*-methylether (**20**)

Table S31 ^{1}H and ^{13}C NMR data of compound **21** (500 MHz, CD_3OD) and 5′-hydroxyasperentin (500 MHz, CD_3OD)

Position /DEPT	21		5′-hydroxyasperentin[11]	
	δ_H	δ_C	δ_H	δ_C
1 C		171.8		171.7
2 O				
3 CH	4.66 (t, 10.0)	66.7	4.56 (m)	66.7
4 CH_2	2.92 (dd, 16.2, 3.5), 2.87 (d, 10.5)	34.7	2.73-2.83 (m)	34.6
4a C		143.8		143.8
5 CH	6.22 (s)	102.5	6.10 (d, 2.3)	102.5
6 C		166.6		166.6
7 CH	6.20 (s)	108.2	6.11 (d, 2.3)	108.2
8 C		165.9		165.9
8a C		101.9		101.8
9 CH_2	1.79-1.93 (m)	40.6	1.68-1.85 (m)	40.6
1′ O				
2′ CH	3.94-4.01 (m)	72.7	3.84-3.92 (m)	72.6
3′ CH_2	1.73 (dt, 9.5, 4.0)	27.5	1.59-1.85 (m)	27.4
4′ CH_2	1.29-1.37 (m)	30.3	1.20-1.25 (m)	30.3
5′ CH	3.71 (ddd, 9.0, 4.5, 4.5)	69.2	3.61 (m)	69.2
6′ CH	3.94-4.01 (m)	78.1	3.84-3.92 (m)	78.1
6′-CH_3	1.20 (d, 7.0)	13.4	1.10 (d, 6.4)	13.4

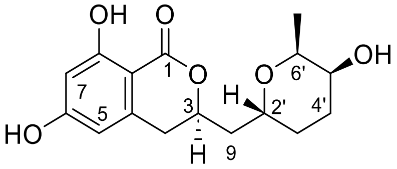

Figure S95 Structure of 5′-hydroxyasperentin (**21**)

Table S32 ^{1}H and ^{13}C NMR data of compound **22** (500 MHz, CD_3OD) and 4′-hydroxyasperentin (500 MHz, CD_3OD)

**Position /DEPT**	**22**		**4′-hydroxyasperentin**[11]	
	***δ*H**	***δ*C**	***δ*H**	***δ*C**
1 C		171.2		171.2
2 O				
3 CH	4.63 (dtt, 9.8, 3.8, 1.0)	77.8	4.62 (m)	77.8
4 CH_2	2.92 (m)	34.0	2.92 (m)	34.0
4a C		143.3		143.3
5 CH	6.20 (d, 2.0)	102.1	6.20 (d, 2.3)	102.0
6 C		166.2		166.2
7 CH	6.22 (d, 2.5)	107.8	6.21 (d, 2.3)	107.8
8 C		165.5		165.5
8a C		101.5		101.4
9 CH_2	2.29 (ddd, 14.8, 11.0, 3.8),<br>1.78 (ddd, 14.8, 9.2, 3.5)	37.2	2.29 (m),<br>1.75-1.84 (m)	37.1
1′ O				
2′ CH	4.37 (ddd, 7.9, 5.8, 2.5)	69.8	4.38 (m)	69.8
3′ CH_2	1.80-1.85 (m),<br>1.63 (ddd, 12.8, 11.0, 5.8)	39.0	1.75-1.84 (m),<br>1.63 (m)	39.0
4′ CH	3.94 (ddd, 15.0, 10.5, 4.5)	64.5	3.94 (m)	64.5
5′ CH_2	1.92 (quintet d, 12.5, 2.0),<br>1.14-1.19 (m)	43.5	1.92 (m),<br>1.30 (m)	43.4
6′ CH	3.78 (ddt, 11.4, 6.2, 2.5)	66.2	3.77 (m)	66.2
6′-CH_3	1.17 (d, 6.0)	21.9	1.17 (d, 6.4)	21.9

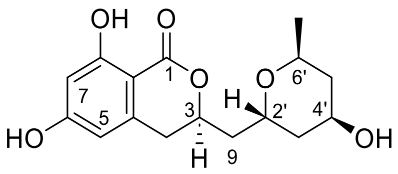

Figure S96 Structure of 4′-hydroxyasperentin (**22**)

Table S33 ^{1}H and ^{13}C NMR data of compound **23** (400 MHz, $CDCl_3$) and tetrahydroauroglaucin (500 MHz, $CDCl_3$)

Position /DEPT	**23** δ_H	**23** δ_C	**Tetrahydroauroglaucin**[14] δ_H	**Tetrahydroauroglaucin**[14] δ_C
1 C		117.1		117.1
2 C		155.1		155.1
3 C		130.3		130.4
4 CH	7.01 (s)	125.0	7.02 (s)	125.0
5 C		144.8		144.8
6 C		124.0		123.9
1′ CH	6.48 (d, 16.0)	120.1	6.48 (d, 16.2)	120.1
2′ CH	5.98 (td, 16.0, 7.2)	142.7	5.98 (td, 16.5, 7.3)	142.7
3′ CH_2	2.32 (q, 7.2)	33.4	2.32 (q, 7.3)	33.4
4′ CH_2	1.52 (quintet, 7.2)	28.7	1.52 (quin, 7.3)	28.7
5′ CH_2	1.35 (m)	31.4	1.35 (m)	31.4
6′ CH_2	1.35 (m)	22.4	1.35 (m)	22.4
7′ CH_3	0.92 (t, 7.2)	14.0	0.92 (t, 7.3)	14.0
1″ CH_2	3.31 (d, 7.6)	27.2	3.32 (d, 7.3)	27.2
2″ CH	5.29 (m)	121.0	5.29 (m)	121.0
3″ C		133.9		133.9
4″ CH_3	1.70 (s)	17.7	1.70 (s)	17.8
5″ CH_3	1.76 (s)	25.8	1.75 (s)	25.8
1-CHO	10.09 (s)	196.3	10.10 (s)	196.3
2-OH	11.73 (s)		11.73 (s)	
5-OH	5.02 (brs)		4.98 (brs)	

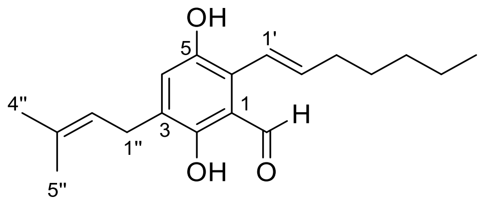

Figure S97 Structure of tetrahydroauroglaucin (**23**)

Table S34 ^{1}H and ^{13}C NMR data of compound **24** (400 MHz, $CDCl_3$) and flavoglaucin (500 MHz, $CDCl_3$)

Position /DEPT		**24**		**Flavoglaucin**[14]	
		δ_H	δ_C	δ_H	δ_C
1	C		117.3		117.3
2	C		155.8		155.8
3	C		128.5		128.6
4	CH	6.89 (s)	125.7	6.89 (s)	125.7
5	C		145.0		145.0
6	C		128.6		128.6
1′	CH_2	2.88 (t, 7.6)	24.0	2.88 (t, 7.6)	23.9
2′	CH_2	1.58 (quin, 8.0)	32.0	1.58 (quin, 7.6)	32.0
3′	CH_2	1.40 (quin, 8.4)	29.6	1.40 (quin, 7.6)	29.6
4′	CH_2	1.30 (m)	29.1	1.30 (m)	29.1
5′	CH_2	1.28 (m)	31.8	1.28 (m)	31.8
6′	CH_2	1.28 (m)	22.6	1.28 (m)	22.6
7′	CH_3	0.88 (t, 7.6)	14.0	0.88 (t, 7.0)	14.0
1″	CH_2	3.29 (d, 7.2)	27.0	3.29 (d, 7.3)	27.0
2″	CH	5.28 (t, 7.2)	121.2	5.28 (m)	121.2
3″	C		133.8		133.8
4″	CH_3	1.70 (s)	17.8	1.70 (s)	17.8
5″	CH_3	1.76 (s)	25.8	1.75 (s)	25.8
1-CHO		10.25 (s)	195.5	10.25 (s)	195.5
2-OH		11.92 (s)		11.92 (s)	
5-OH		4.38 (brs)		4.41 (brs)	

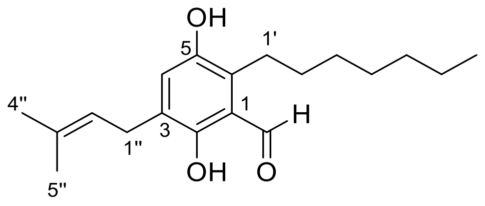

Figure S98 Structure of flavoglaucin (**24**)

Table S35 ^{1}H and ^{13}C NMR data of compound **25** (400 MHz, $CDCl_3$) and auroglaucin (500 MHz, Acetone-d_6)

Position /DEPT	**25** δ_H	**25** δ_C	**Auroglaucin**[14] δ_H	**Auroglaucin**[14] δ_C
1 C		117.1		118.1
2 C		154.7		155.3
3 C		129.5		130.0
4 CH	6.89 (s)	125.0	7.05 (s)	125.8
5 C		146.3		148.1
6 C		125.1		126.1
1′ CH	6.69 (d, 15.6)	121.9	6.91 (d, 15.6)	123.4
2′ CH	6.35 (ddd, 15.0, 7.0, 3.2)	139.0	6.62 (m)	139.2
3′ CH	6.25 (dd, 7.2, 3.2)	129.9	6.39 (m)	131.6
4′ CH	6.25 (dd, 7.2, 3.2)	135.2	6.39 (m)	135.6
5′ CH	6.08 (m)	131.4	6.19 (m)	132.7
6′ CH	5.74 (dq, 15.0, 7.0)	131.7	5.80 (dq, 15.0, 6.7)	131.6
7′ CH_3	1.74 (d, 6.4)	18.3	1.77 (d, 6.7)	18.4
1″ CH_2	3.25 (d, 7.6)	27.2	3.27 (d, 7.6)	27.8
2″ CH	5.22 (dt, 7.4, 1.2)	121.2	5.28 (m)	122.3
3″ C		133.6		133.8
4″ CH_3	1.64 (s)	17.6	1.69 (s)	17.8
5″ CH_3	1.65 (s)	25.6	1.71 (s)	25.8
1-CHO	10.01 (s)	196.5	10.16 (s)	197.6
2-OH	11.70 (s)		11.90 (s)	

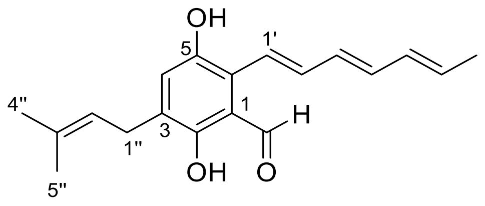

Figure S99 Structure of auroglaucin (**25**)

Table S36 ^{1}H and ^{13}C NMR data of compound **26** (400 MHz, $CDCl_3$) and isodihydroauroglaucin (500 MHz, $CDCl_3$)

Position /DEPT		26		Isodihydroauroglaucin[14]	
		δ_H	δ_C	δ_H	δ_C
1	C		117.3		117.3
2	C		155.8		155.8
3	C		128.9		128.9
4	CH	6.90 (s)	125.9	6.90 (s)	125.8
5	C		145.2		145.1
6	C		127.5		127.4
1′	CH_2	2.98 (t, 7.6)	24.1	2.98 (t, 7.5)	24.1
2′	CH_2	2.34 (q, 7.6)	34.2	2.34 (q, 7.5)	34.2
3′	CH	6.02 (m)	132.0	6.02 (m)	132.0
4′	CH	6.02 (m)	131.1	6.00 (m)	131.1
5′	CH	5.59 (m)	128.3	5.59 (m)	128.3
6′	CH	5.59 (m)	129.4	5.58 (m)	129.4
7′	CH_3	1.73 (d, 7.2)	18.0	1.73 (d, 6.7)	18.0
1″	CH_2	3.29 (d, 6.8)	27.0	3.29 (d, 7.3)	27.0
2″	CH	5.28 (m)	121.1	5.28 (m)	121.1
3″	C		133.8		133.9
4″	CH_3	1.69 (s)	17.7	1.70 (s)	17.8
5″	CH_3	1.75 (s)	25.8	1.76 (d, 1.0)	25.8
1-CHO		10.22 (s)	195.4	10.23 (s)	195.4
2-OH		11.93 (s)		11.94 (s)	
5-OH		4.78 (brs)		4.53 (brs)	

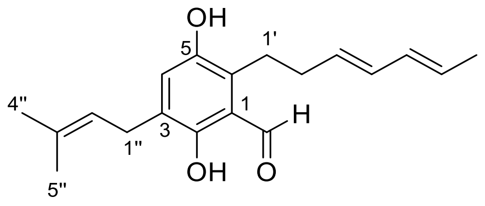

Figure S100 Structure of isodihydroauroglaucin (**26**)

Table S37 ^{1}H and ^{13}C NMR data of compound **27** (400 MHz, $CDCl_3$ and CD_3OD, 4/1 v/v), and ^{13}C NMR data of echinulin (500 MHz, $CDCl_3$)

Position /DEPT		27		Echinulin[15]
		δ_H	δ_C	δ_C
1	NH			
2	C		141.2	141.4
3	C		104.0	104.1
3a	C		128.9	128.9
4	CH	7.05 (s)	114.9	115.0
5	C		133.7	133.9
6	CH	6.72 (s)	122.7	122.9
7	C		123.4	123.4
7a	C		132.0	132.2
8	CH_2	3.53-3.56 (m),<br>3.08 (dd, 14.6, 11.4)	29.9	29.4
9	CH	4.28 (dd, 11.6, 2.4)	54.8	54.5
10	C		168.4	168.3
11	NH			
12	CH	3.96 (q, 6.6)	50.6	50.8
13	C		168.3	167.7
14	NH			
15	C		38.9	39.9
16	CH	6.02 (dd, 17.6, 10.8)	145.7	145.7
17	CH_2	5.08 (d, 17.6),<br>5.06 (d, 10.4)	112.1	112.3
18	CH_3	1.42 (s)	27.7	27.9
19	CH_3	1.42 (s)	27.6	27.8
20	CH_2	3.30 (d, 7.6)	34.5	34.6
21	CH	5.27 (t, 7.4)	124.4	124.5
22	C		131.5	131.6
23	CH_3	1.66 (s)	25.5	25.7
24	CH_3	1.65 (s)	17.7	17.9
25	CH_2	3.45 (d, 6.8)	31.1	31.4
26	CH	5.32 (t, 7.6)	122.7	122.9
27	C		132.9	132.9
28	CH_3	1.72 (s)	25.6	25.8
29	CH_3	1.78 (s)	17.7	17.9
30	CH_3	1.44 (s)	19.9	19.9

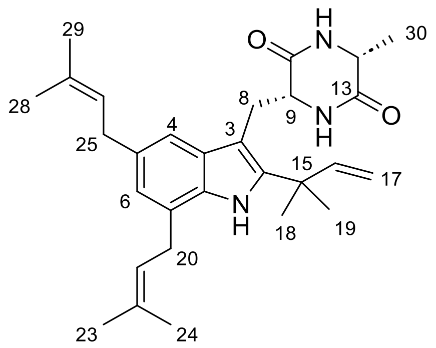

Figure S101 Structure of echinulin (**27**)

Table S38 ^{1}H and ^{13}C NMR data of compound **28** (500 MHz, $CDCl_3$ and CD_3OD, 4/1 v/v) and physcion (400 MHz, $CDCl_3$)

**Position /DEPT**	**28**		**Physcion**[16]	
	δ_H	δ_C	δ_H	δ_C
1 C		163.4		165.2
2 CH	6.76 (d, 1.5)	104.9	6.68	106.8
3 C		163.7		166.6
4 CH	7.29 (d, 1.5)	107.4	7.36	108.2
5 CH	7.53 (s)	119.9	7.62	121.3
6 C		146.7		148.4
7 CH	7.07 (s)	124.7	7.07	124.5
8 C		162.2		162.5
9 C		187.3		190.8
10 C		183.7		182.0
11 C		132.4		133.2
12 C		114.9		113.7
13 C		113.9		110.3
14 C		137.4		135.3
3-OCH_3	4.01 (s)	56.4	3.93	56.1
6-CH_3	2.42 (s)	21.8	2.44	22.2
OH	13.18 (s)		12.30, 12.11	

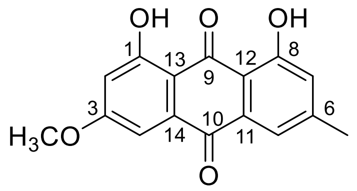

Figure S102 Structure of physcion (**28**)

Table S39 The melting point and specific rotation of all isolated compounds compared with their literatures

NO.	Melting point (°C)		Specific rotation value ($[\alpha]_D^{℃}$, degree)	
	Compound	Literature	Compound	Literature
**1**	244-245	-	-	-
**2**	135-136	-	$[\alpha]_D^{25.6}$ -123.6 (c 1.0, $CHCl_3$)	-
**3**	53-55	58[17]	$[\alpha]_D^{24.6}$ -44.8 (*c* = 1.0, $CHCl_3$)	$[\alpha]_D^{22.0}$ -100.0 (*c* = 1.0, $CHCl_3$)[17]
**4**	63-65	65-66[17]	$[\alpha]_D^{24.8}$ -57.4 (*c* = 1.0, $CHCl_3$)	$[\alpha]_D^{27.0}$ -144.3 (*c* = 1.0, $CHCl_3$)[3]
**5**	242-244	245-246[17]	$[\alpha]_D^{28.9}$ -76.9 (*c* = 1.0, EtOH)	$[\alpha]_D^{26.0}$ -51.2 (*c* = 0.02, EtOH)[3]
**6**	258-260	252-255[3]	$[\alpha]_D^{26.5}$ -104.4 (*c* = 1.0, $CHCl_3$)	$[\alpha]_D^{28.0}$ -17.7 (*c* = 0.55, $CHCl_3$)[3]
**7**	176-180	180-190[18]	$[\alpha]_D^{27.0}$ +80.0 (*c* = 1.0, EtOH)	$[\alpha]_D^{25.0}$ +20.6 (*c* = 0.025, EtOH)[4]
**8**	240-243	-	$[\alpha]_D^{27.0}$ -180.0 (*c* = 0.5, $CHCl_3$)	$[\alpha]_D^{20.0}$ -228.0 (*c* = 0.035, $CHCl_3$)[5]
**9**	251-253	258-265[7]	$[\alpha]_D^{27.4}$ -57.0 (*c* = 1.0, $CHCl_3$)	$[\alpha]_D^{23.0}$ -39.3 (*c* = 1.0, MeOH)[7]
**10**	270-273	260-265[7]	$[\alpha]_D^{29.2}$ +14.6 (*c* = 1.0, MeOH)	-
**11**	133-134	133-135[8]	$[\alpha]_D^{25.0}$ -7.0 (*c* = 0.1, $CHCl_3$)	-
**12**	149-150	149-151[8]	$[\alpha]_D^{25.4}$ -2.8 (*c* = 0.1, $CHCl_3$)	-
**13**	132-134	129-131[19]	$[\alpha]_D^{20.0}$ +8.8 (*c* = 0.1, MeOH)	-
**14**	-	-	-	$[\alpha]_D^{20.0}$ +110.0 (*c* = 0.39, EtOH)[9]
**15**	-	-	$[\alpha]_D^{27.5}$ +11.4 (c = 1.0, CH_2Cl_2)	-
**16**	-	-	$[\alpha]_D^{27.6}$ -25.6 (c = 1.0, CH_2Cl_2)	-
**17**	-	-	$[\alpha]_D^{27.8}$ -38.4 (c = 0.1, EtOH)	-
**18**	183-184	184-186[11]	$[\alpha]_D^{23.7}$ -50.2 (*c* = 0.1, EtOH)	$[\alpha]_D^{20.0}$ -70.0 (*c* = 0.1, EtOH)[11]
**19**	229-230	230-235[11]	$[\alpha]_D^{23.6}$ +17.8 (*c* = 0.1, EtOH)	$[\alpha]_D^{20.0}$ +20.0 (*c* = 0.1, EtOH)[11]
**20**	93-94	93-98[11]	$[\alpha]_D^{23.8}$ +53.5 (c = 0.1, EtOH)	$[\alpha]_D^{20.0}$ +70.0 (*c* = 0.1, EtOH)[11]
**21**	219-220	220-223[11]	$[\alpha]_D^{23.9}$ -22.4 (*c* = 0.1, EtOH)	$[\alpha]_D^{20.0}$ -36.1 (*c* = 0.08, EtOH)[11]
**22**	195-196	197-199[11]	$[\alpha]_D^{24.0}$ -20.1 (*c* = 0.1, EtOH)	$[\alpha]_D^{20.0}$ -36.1 (*c* = 0.08, EtOH)[11]
**23**	74-75	74-75[20]	$[\alpha]_D^{27.5}$ -30.4 (*c* = 1.0, CH_2Cl_2)	-
**24**	110-111	111-112[20]	$[\alpha]_D^{27.8}$ -7.0 (*c* = 1.0, CH_2Cl_2)	-
**25**	146-147	151-152[21]	$[\alpha]_D^{27.9}$ -21.8 (*c* = 1.0, CH_2Cl_2)	-
**26**	111-112	114-115[20]	$[\alpha]_D^{28.0}$ -9.2 (*c* = 1.0, CH_2Cl_2)	-
**27**	241-242	241-244[21]	$[\alpha]_D^{23.6}$ -116.8 (*c* = 1.0, MeOH)	-
**28**	207-208	208.5-210[21]	$[\alpha]_D^{25.4}$ -11.4 (*c* = 1.0, MeOH)	-

HPLC identification and quantification of asperentin (18) and (3*R*,2′*R*,6′*S*)-asperentin-6-*O*-methylether (20)

1. Identification of compounds 18 and 20

The chromatograms were identified by comparing their retention times and UV absorption patterns with compounds **18** and **20**. The examination of crude EtOAc of broth and mycelium extracts from PDB and YM media showed very similar compounds profiles with the intensity of compounds **18** and **20** being notably different in both media. Compound **18** showed an absorption band at a retention time of 8.84 minutes (Figure S103) and **20** displayed at a retention time of 13.13 minutes (Figure S104).

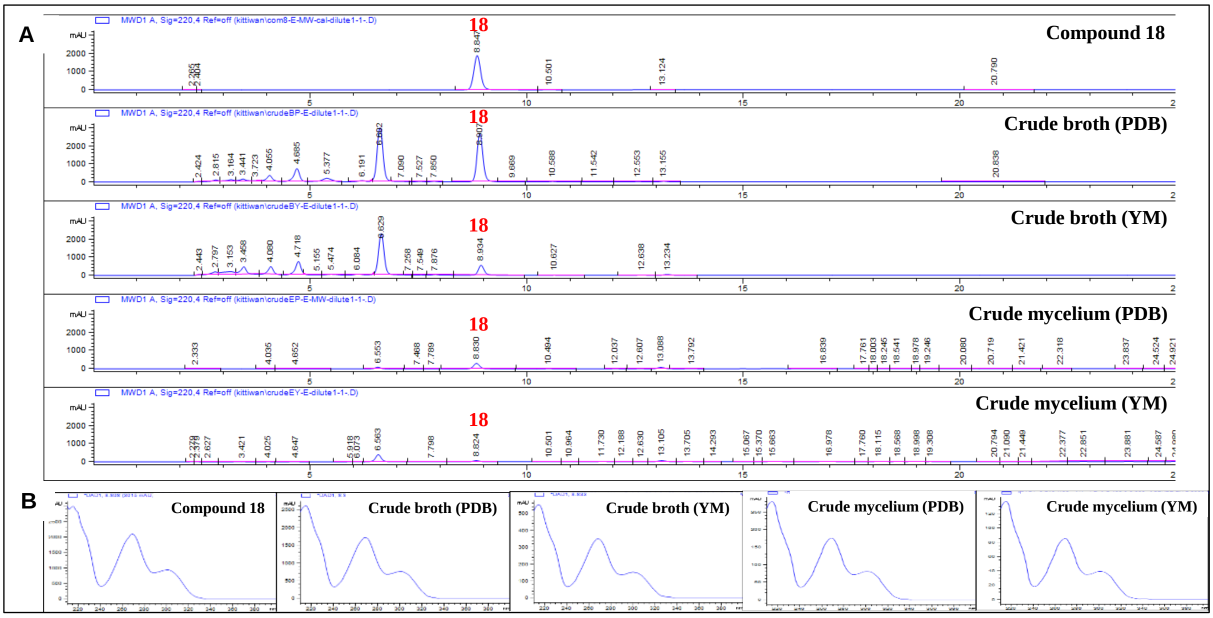

Figure S 103 (A) HPLC chromatogram and (**B**) UV spectrum of all crude extracts and compound **18**

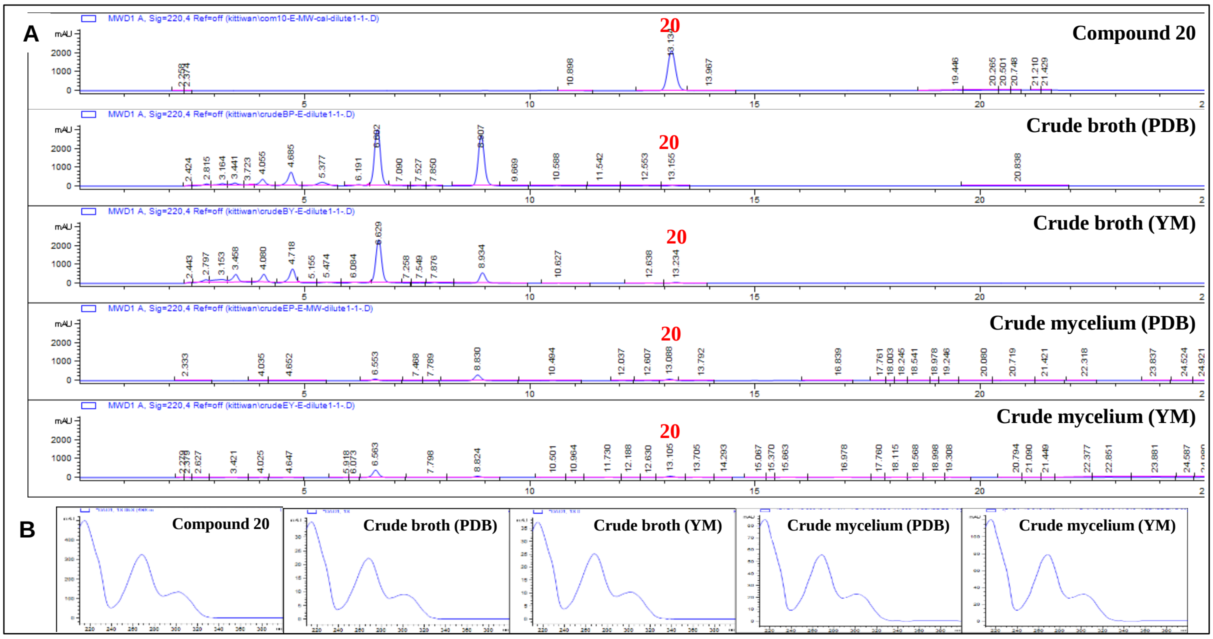

Figure S104 (**A**) HPLC chromatogram and (**B**) UV spectrum of all crude extracts and compound **20**

2. Method validation

2.1. Calibration curve and linearity

The calibration curves of compounds **18** and **20** (Figure S105) were constructed in the range of 15.6-1000 and 7.8-500 μg/mL, respectively. Regression equations for **18** and **20** were obtained as y = 21.377x - 25.498 and y = 27.245x + 6.4168; the correlation coefficients (*R*2) were 0.9999 and 0.9999, which showed a good linearity. The correlation coefficient is commonly used to evaluate the degree of linear relation between peak area (y-axis) and concentration (x-axis) in the range of the analytical procedure.

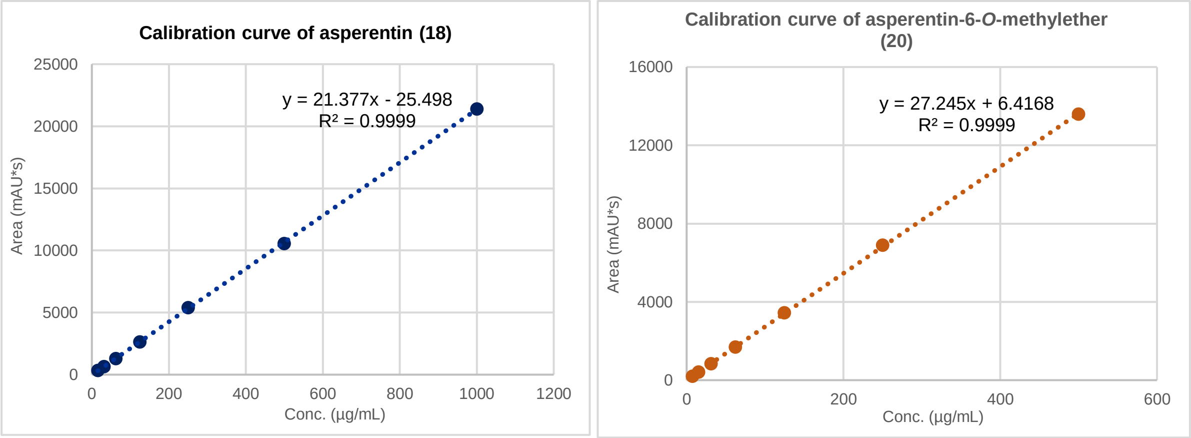

Figure S105 Calibration curves of compounds **18** and **20**

2.2. LOD and LOQ

The limit of detection (LOD) of **18** and **20** was calculated as $3\sigma/S$ (σ is standard deviation and S is slope of calibration curve) and the limit of quantification (LOQ) was calculated as $10\sigma/S$. Compound **18** showed an LOD of 0.4416 μg/mL and an LOQ of 1.4721 μg/mL. Compound **20** showed an LOD of 0.1795 μg/mL and an LOQ of 0.5984 μg/mL.

2.3. Intra and inter-day assay precisions and accuracy

Results of intra- and inter-day precisions and accuracy are shown in Table S31. All the values of relative standard deviation (RSD) in the intra and inter day estimates were below 1%. The precision results were accepted with limits recommended in the literature.[22] Accuracy was indicated as the percentage of measured value and the reference value. %Recovery of compound **18** was in the range of 105.74-106.19% and compound **20** was in the range of 92.16-95.44%. Based on ICH guideline 2005, the acceptable of % recovery must in the range of 85-115%.[22]

Table S40 Repeatability and intermediate precision data of compounds **18** and **20**

NO.	Conc. (µg/mL)	Repeatability (n = 3) measured values (µg/mL)	Mean (µg/mL)	RSD (%)	Recovery (%)	Intermediate precision (n = 3) measured values (µg/mL)	Mean (µg/mL)	RSD (%)
**18**	125	132.1092	132.1809	0.18	105.74	132.1809	131.9039	0.51
		132.4460				132.3899		
		131.9876				131.1409		
	250	264.3401	265.4675	0.37	106.19	264.3401	264.5048	0.06
		265.9586				264.5412		
		266.1037				264.6332		
	500	530.0182	530.2832	0.04	106.06	530.0182	530.4231	0.07
		530.3877				530.5904		
		530.4438				530.6606		
**20**	125	119.0042	119.2983	0.26	95.44	119.0042	119.6651	0.48
		119.2631				120.0192		
		119.6278				119.9718		
	250	234.0177	232.0059	0.79	92.16	234.0177	233.0870	0.37
		230.4074				232.3025		
		231.5926				232.9407		
		464.0082				464.0082		

	500	463.3518	464.4276	0.29	92.88	465.2092	464.7724	0.14
		465.9227				465.0998		

Table S41 Weight of crude extracts from *X. longipes* SWUF08-81

conditions	Volume of broth (L)	Weight of broth crude extracts (g)	Weight of dried mycelia (g)	Weight of mycelia crude extracts (g)
YM	14	9.86	53.06	7.09
PDB	14	5.15	52.03	3.92

References

1. Kalinova, B.; Kindl, J.; Jiros, P.; Zacek, P.; Vasickova, S.; Budesinsky, M.; Valterova, I., Composition and electrophysiological activity of constituents identified in male wing gland secretion of the bumblebee parasite *Aphomia sociella*. *J. Nat. Prod.* **2009,** *72* (1), 8-13.
2. Chacón-Morales, P.; Amaro-Luis, J. M.; Bahsas, A., Isolation and characterization of (+)-mellein, the first isocoumarin reported in *Stevia* genus. *Avances en Quimica* **2013,** *8* (3), 145-151.
3. Klaiklay, S.; Rukachaisirikul, V.; Sukpondma, Y.; Phongpaichit, S.; Buatong, J.; Bussaban, B., Metabolites from the mangrove-derived fungus *Xylaria cubensis* PSU-MA34. *Arch. Pharm. Res.* **2012,** *35* (7), 1127-31.
4. Fujii, Y.; Tani, H.; Ichinoe, M.; Nakajima, H., Zygosporin D and two new cytochalasins produced by the fungus *Metarrhizium anisopliae*. *J. Nat. Prod.* **2000,** *63* (1), 132-5.
5. Espada, A.; Rivera-Sagredo, A.; de la Fuente, J. M.; Hueso-Rodríguez, J. A.; Elson, S. W., New cytochalasins from the fungus *Xylaria hypoxylon*. *Tetrahedron* **1997,** *53* (18), 6485-6492.
6. Merifield, E.; Thomas, E. J., Total synthesis of cytochalasin D: total synthesis and full structural assignment of cytochalasin O. *J. Chem. Soc., Perkin Trans. 1* **1999,** (22), 3269-3283.
7. Edwards, R. L.; Maitland, D. J.; Whalley, A. J. S., Metabolites of the higher fungi. Part 24. Cytochalasin N, O, P, Q, and R. New cytochalasins from the fungus *Hypoxylon terricola Mill*. *Journal of the Chemical Society, Perkin Transactions 1* **1989,** (1).
8. Tansuwan, S.; Pornpakakul, S.; Roengsumran, S.; Petsom, A.; Muangsin, N.; Sihanonta, P.; Chaichit, N., Antimalarial benzoquinones from an endophytic fungus, *Xylaria* sp. *J. Nat. Prod.* **2007,** *70* (10), 1620-3.
9. Shiono, Y.; Murayama, T.; Takahashi, K.; Okada, K.; Katohda, S.; Ikeda, M., Three oxygenated cyclohexenone derivatives produced by an endophytic fungus. *Biosci. Biotechnol. Biochem.* **2005,** *69* (2), 287-92.
10. Tian, J. K.; Sun, F.; Cheng, Y. Y., Chemical constituents from the roots of *Ranunculus ternatus*. *J. Asian Nat. Prod. Res.* **2006,** *8* (1-2), 35-9.
11. Kimura, Y.; Shimomura, N.; Tanigawa, F.; Fujioka, S.; Shimada, A., Plant growth activities of aspyran, asperentin, and its analogues produced by the fungus *Aspergillus* sp. *Z Naturforsch C J Biosci* **2012,** *67* (11-12), 587-93.

12. Zheng, H.; Zhao, C.; Fang, B.; Jing, P.; Yang, J.; Xie, X.; She, X., Asymmetric total synthesis of cladosporin and isocladosporin. *J Org Chem* **2012,** *77* (13), 5656-63.
13. Fujimoto, H.; Sumino, M.; Okuyama, E.; Ishibashi, M., Immunomodulatory constituents from an Ascomycete, *Chaetomium seminudum*. *J Nat Prod* **2004,** *67* (1), 98-102.
14. Miyake, Y.; Ito, C.; Itoigawa, M.; Osawa, T., Antioxidants produced by *Eurotium herbariorum* of filamentous fungi used for the manufacture of karebushi, dried bonito (Katsuobushi). *Biosci Biotechnol Biochem* **2009,** *73* (6), 1323-7.
15. Li, D.-L.; Li, X.-M.; Li, T.-G.; Dang, H.-Y.; Wang, B.-G., Dioxopiperazine alkaloids produced by the marine mangrove derived endophytic fungus *Eurotium rubrum*. *Helv. Chim. Acta* **2008,** *91* (10), 1888-1893.
16. Danielsen, K.; Aksnes, D. W.; Francis, G. W., NMR study of some anthraquinones from rhubarb. *Magnetic Resonance in Chemistry* **1992,** *30* (4), 359-360.
17. Anderson, J. R.; Edwards, R. L.; Whalley, A. J. S., Metabolites of the higher fungi. Part 21. 3-Methyl-3,4-dihydroisocoumarins and related compounds from the ascomycete family xylariaceae. *J. Chem. Soc., Perkin Trans. 1* **1983**.
18. Minato, H.; Katayama, T., Studies on the metabolites of *Zygosporium masonii*. Part II. Structures of zygosporins D, E, F, and G. *J. Chem. Soc. Perkin 1* **1970,** *1*, 45-7.
19. Deng, K. Z.; Xiong, Y.; Zhou, B.; Guan, Y. M.; Luo, Y. M., Chemical constituents from the roots of *Ranunculus ternatus* and their inhibitory effects on *Mycobacterium tuberculosis*. *Molecules* **2013,** *18* (10), 11859-65.
20. Hamasaki, T.; Kimura, Y., Isolation and structures of four new metabolites from *Aspergillus wentii*. *Agric. Biol. Chem.* **1980,** *47* (1), 163-165.
21. Arai, K.; Aoki, Y.; Yamamoto, Y., Asperinines A and B, dimeric tetrahydroanthracene derivatives from Aspergillus ruber. *Chemical and Pharmaceutical Bulletin* **1989,** *37* (3), 621-625.
22. Borman, P.; Elder, D., *Chapter 5 : Q2 (R1) validation of analytical procedures*. Text and Methodology: 2017.
